# Supplementary material for: Sequence-controlled supramolecular terpolymerization directed by specific molecular recognitions
Source: Nat Commun. 2017 Sep 21;8:634. doi: 10.1038/s41467-017-00683-5 (PMC5608752; doi:10.1038/s41467-017-00683-5)
Supplement: Supplementary file 1 — Supplementary Information [file 41467_2017_683_MOESM1_ESM.pdf]

Description of Supplementary Files

File Name: Supplementary Information
Description: Supplementary Figures, Supplementary Tables, Supplementary Methods and Supplementary References

File Name: Peer Review File

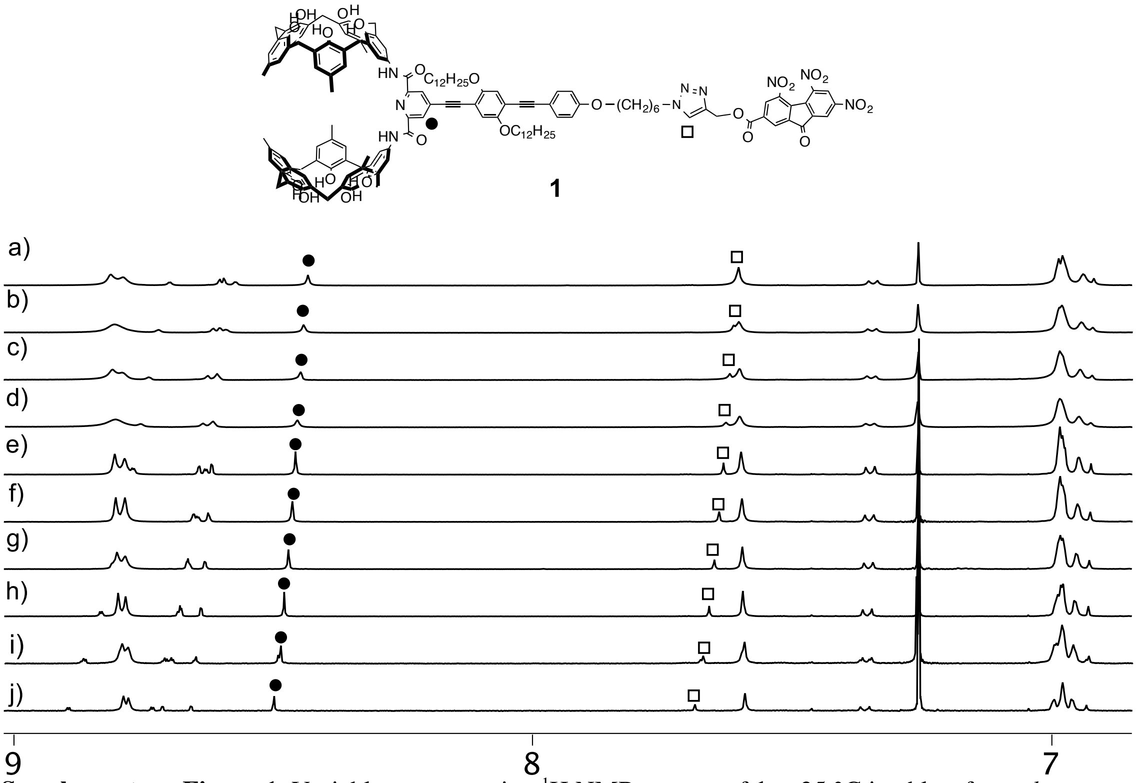

Supplementary Figure 1. Variable concentration ^{1}H NMR spectra of **1** at 25 °C in chloroform-d_1. Concentrations of **1** are (a-j) 10.2, 8.98, 8.01, 6.95, 6.04, 5.04, 4.00, 3.00, 2.05, 1.03 × 10^{-3} mol L^{-1}.

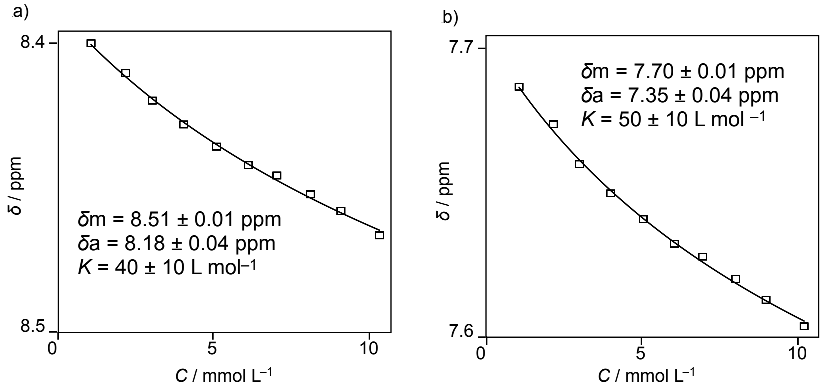

Supplementary Figure 2. The plots and fitting curves of the chemical shift changes of the protons marked (a) with filled circles and (b) with open squares in Supplementary Figure 1 upon concentrating the solution at 25 °C in chloroform-d_1. K denotes the self-association constants for **1**. δ_m and δ_a denote the chemical shifts for the monomeric form and for the polymeric form, respectively.

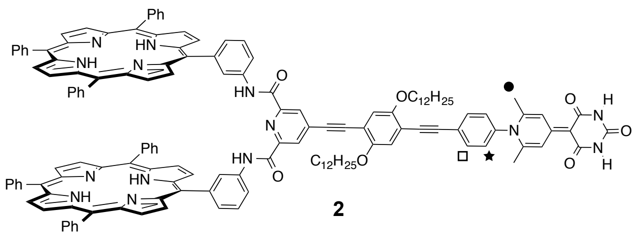

2

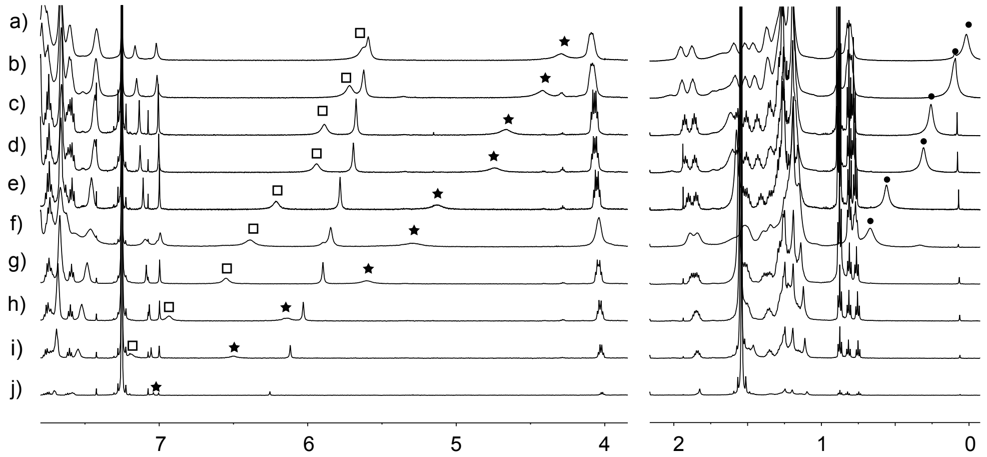

Supplementary Figure 3. Variable concentration ^{1}H NMR spectra of **2** at 25 °C in chloroform-d_1. Concentrations of **2** are (a-j) 25.0, 18.0, 12.5, 10.0, 6.25, 5.00, 3.12, 1.56, 0.78, 0.10 × 10^{-3} mol L^{-1}.

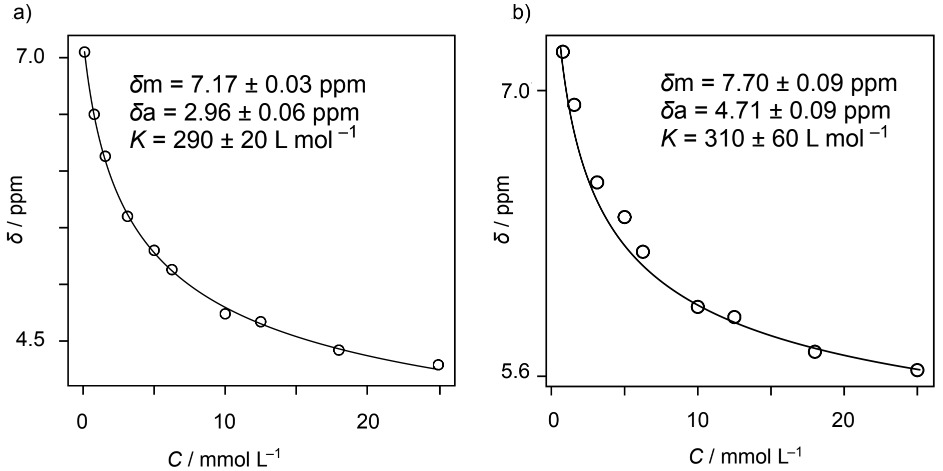

Supplementary Figure 4. The plots and fitting curves of the chemical shift changes of the protons marked (a) with filled stars and (b) with open squares in Supplementary Figure 3 upon concentrating the solution at 25 °C in chloroform-d_1. *K* denotes the self-association constants for **2**. δ_m and δ_a denote the chemical shifts for the monomeric form and for the polymeric form, respectively.

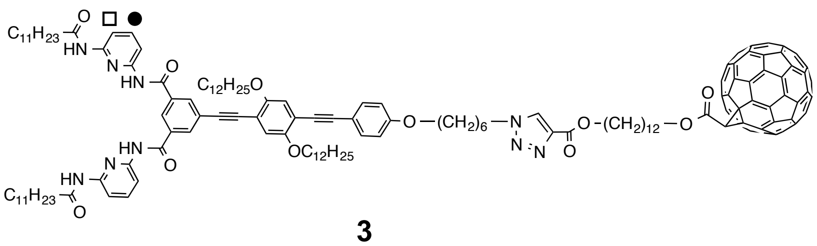

3

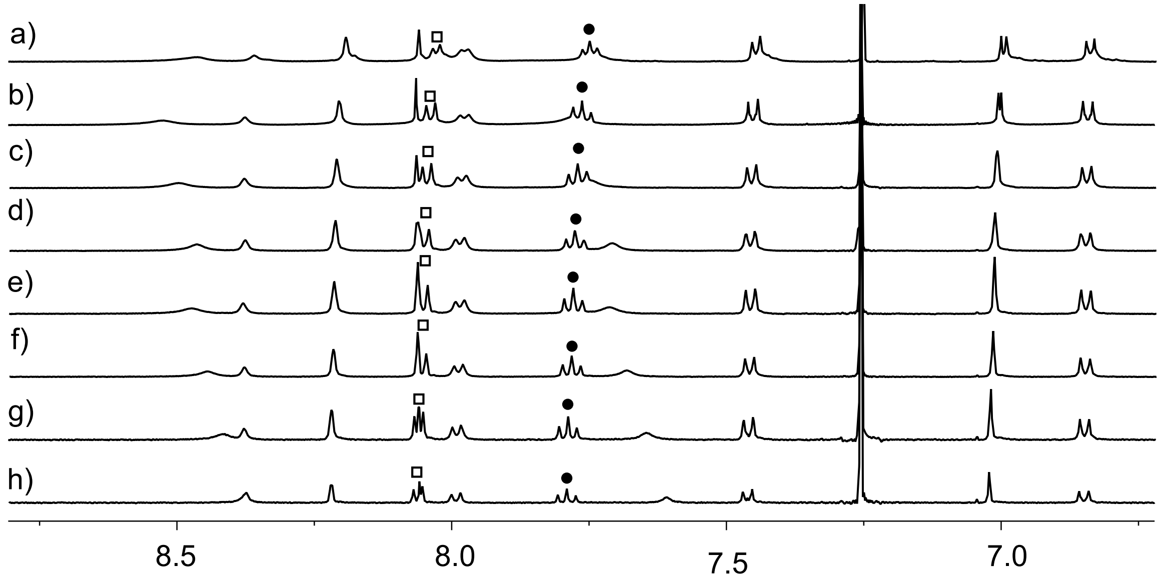

Supplementary Figure 5. Variable concentration ^{1}H NMR spectra of **3** at 25 °C in chloroform-d_1. Concentrations of **3** are (a-h) 15.0, 11.0, 8.02, 6.01, 5.50, 4.01, 2.01, 1.00 × 10^{-3} mol L^{-1}.

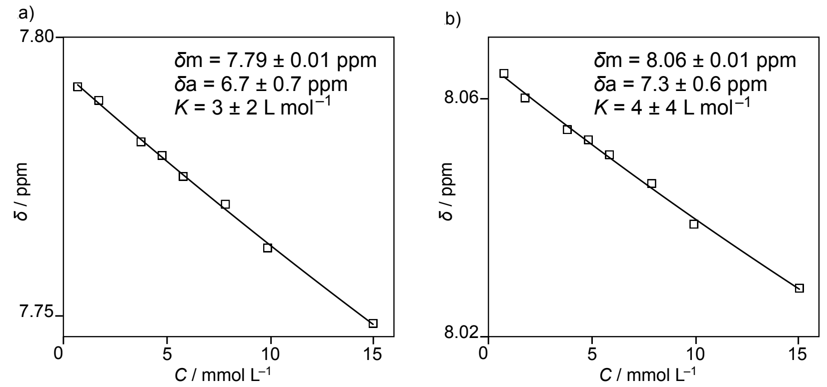

Supplementary Figure 6. The plots and fitting curves of the chemical shift changes of the protons marked (a) with filled circles and (b) with open squares in Supplementary Figure 5 upon concentrating the solution at 25 °C in chloroform-d_1. K denotes the self-association constants for **3**. δ_m and δ_a denote the chemical shifts for the monomeric form and for the polymeric form, respectively.

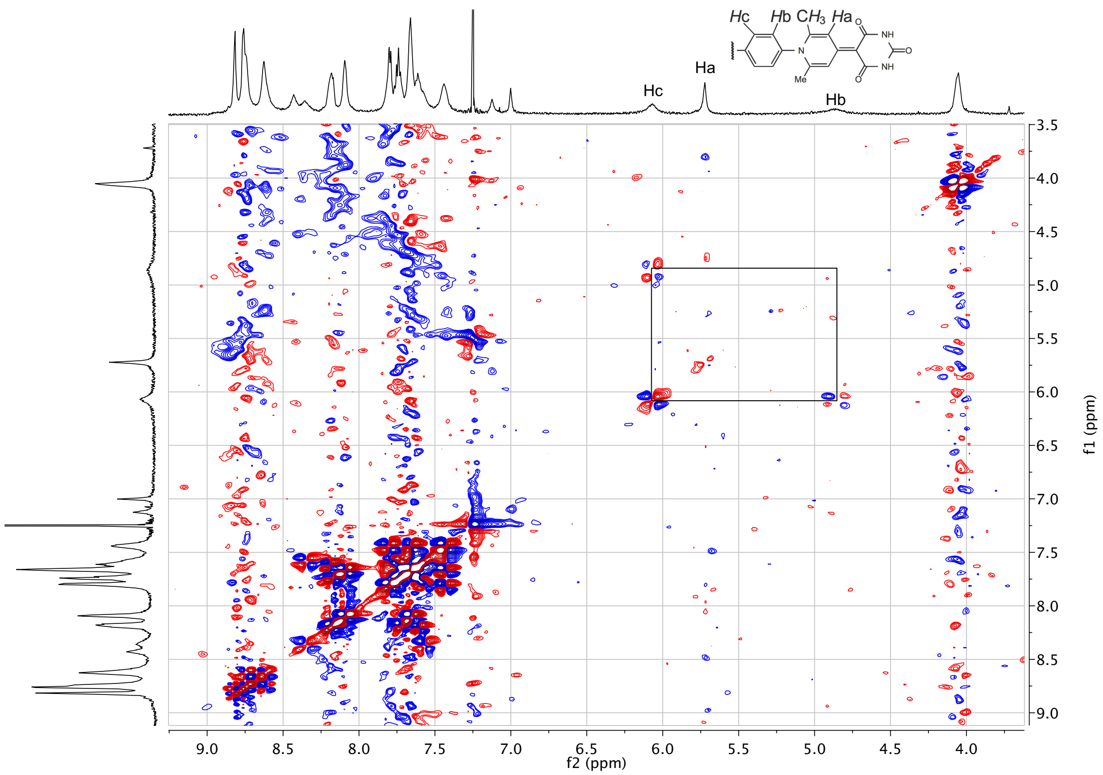

Supplementary Figure 7. DQF-COSY spectrum of **2** in chloroform-d_1.

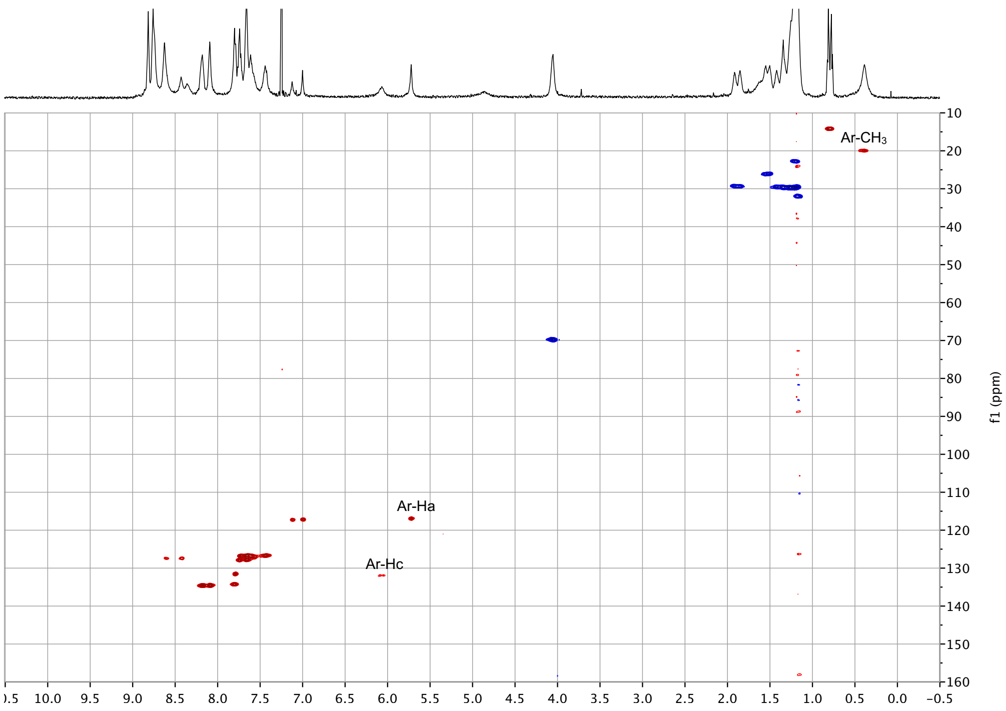

Supplementary Figure 8. HSQC spectrum of **2** in chloroform-d_1. CH_3 and CH peaks are phased up (red), and CH_2 carbons are phase down (blue).

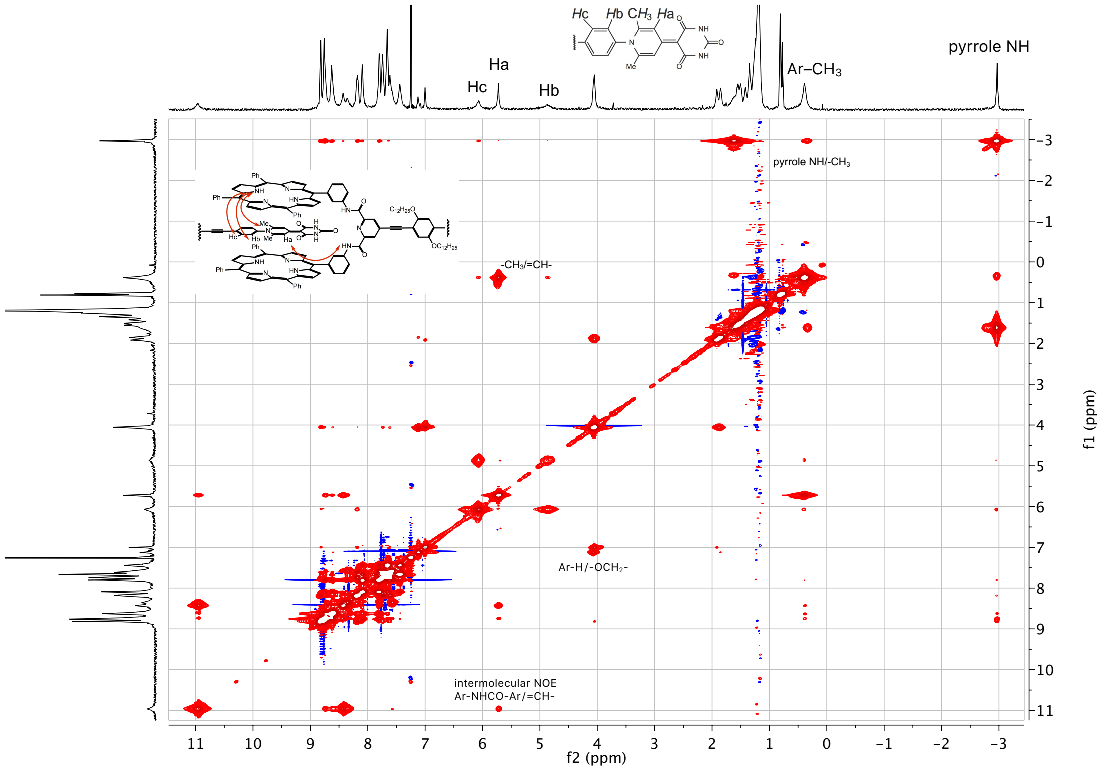

Supplementary Figure 9. NOESY spectrum of **2** in chloroform-d_1.

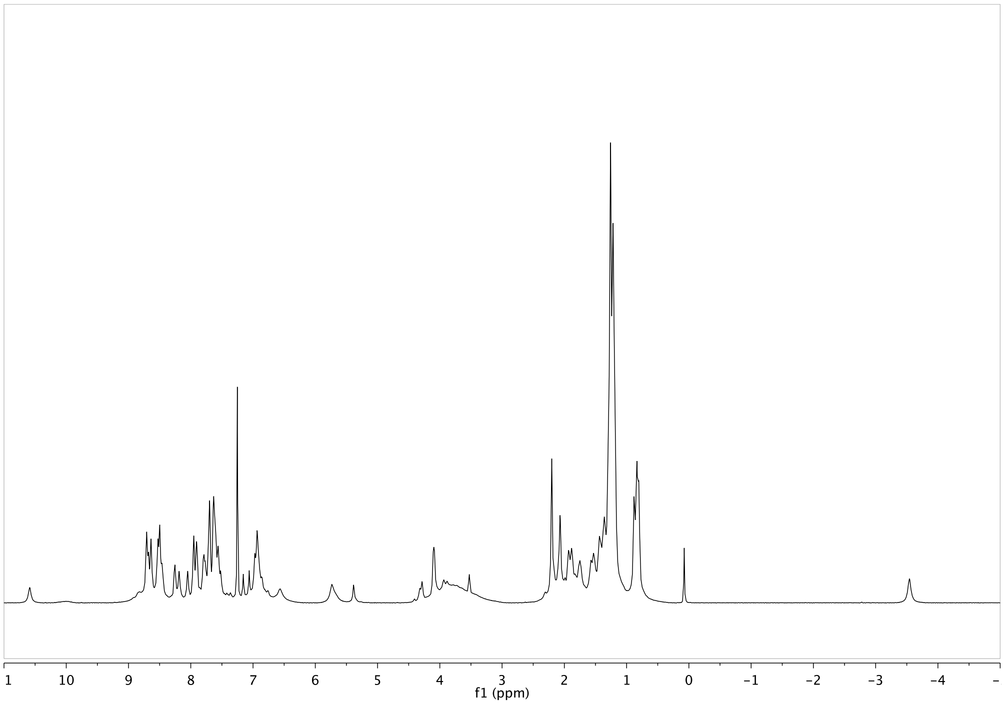

Supplementary Figure 10. ^{1}H NMR spectrum of a mixture of **1** (5.0 x 10^{-3} mol L^{-1}) and **2** (5.0 x 10^{-3} mol L^{-1}) in chloroform-d_1 at 25 °C.

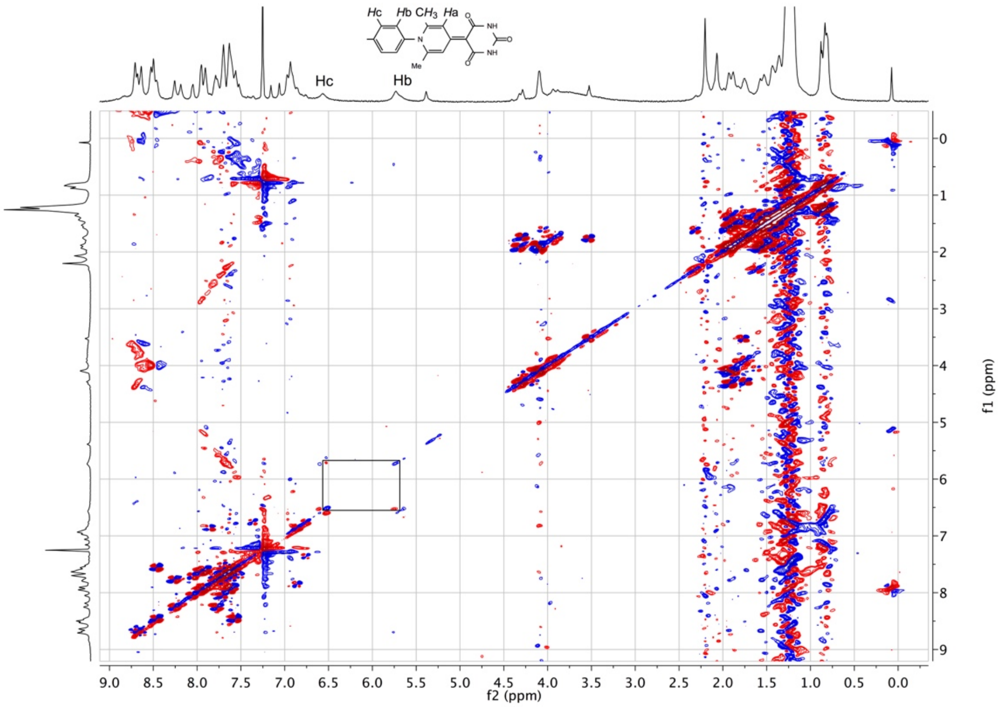

Supplementary Figure 11. DQF-COSY spectrum of a mixture of **1** (5.0 x 10^{-3} mol L^{-1}) and **2** (5.0 x 10^{-3} mol L^{-1}) in chloroform-d_1 at 25 °C.

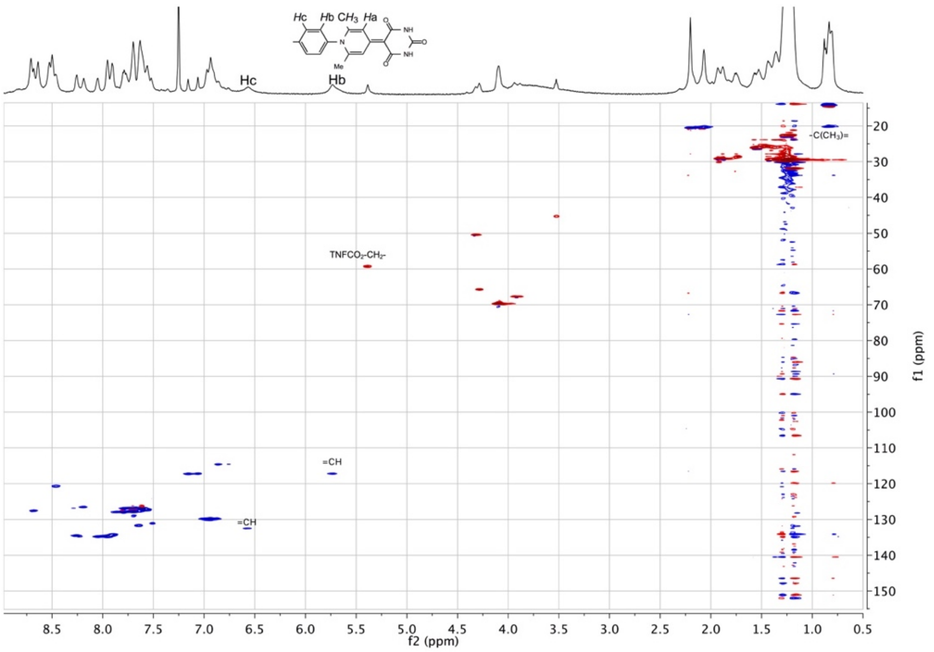

Supplementary Figure 12. HSQC spectrum of a mixture of **1** (5.0×10^{-3} mol L^{-1}) and **2** (5.0×10^{-3} mol L^{-1}) in chloroform-d_1 at 25 °C. CH_3 and CH peaks are phased up (blue), and CH_2 peaks are phased down (red).

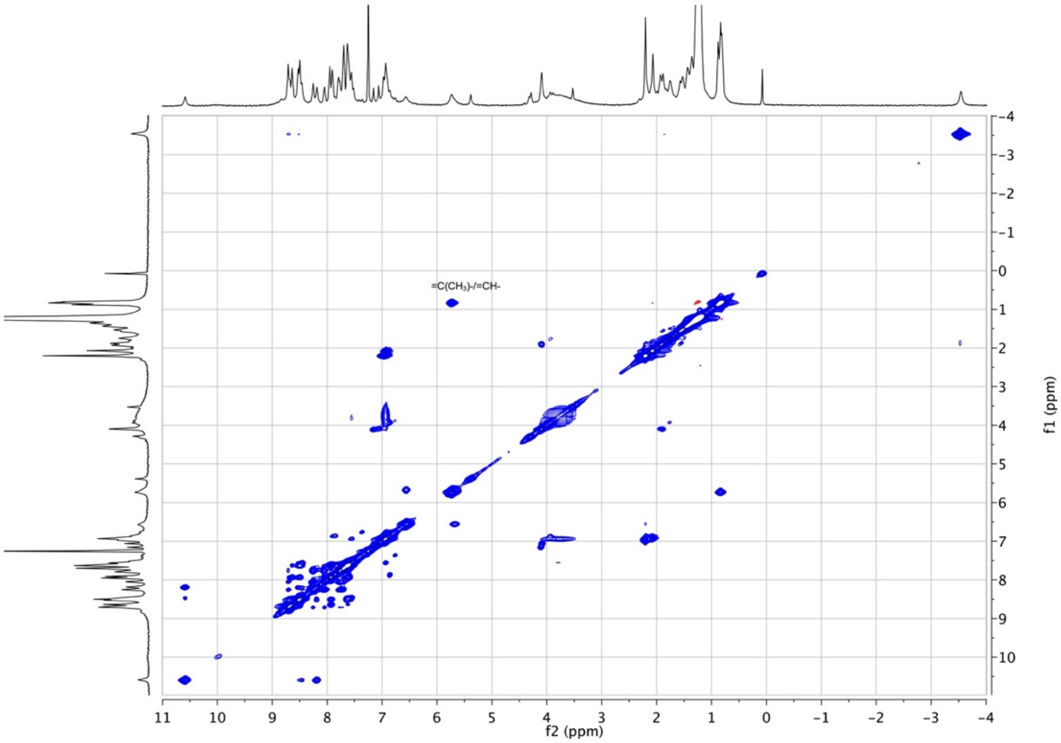

Supplementary Figure 13. NOESY spectrum of a mixture of **1** (5.0×10^{-3} mol L^{-1}) and **2** (5.0×10^{-3} mol L^{-1}) in chloroform-d_1 at 25 °C.

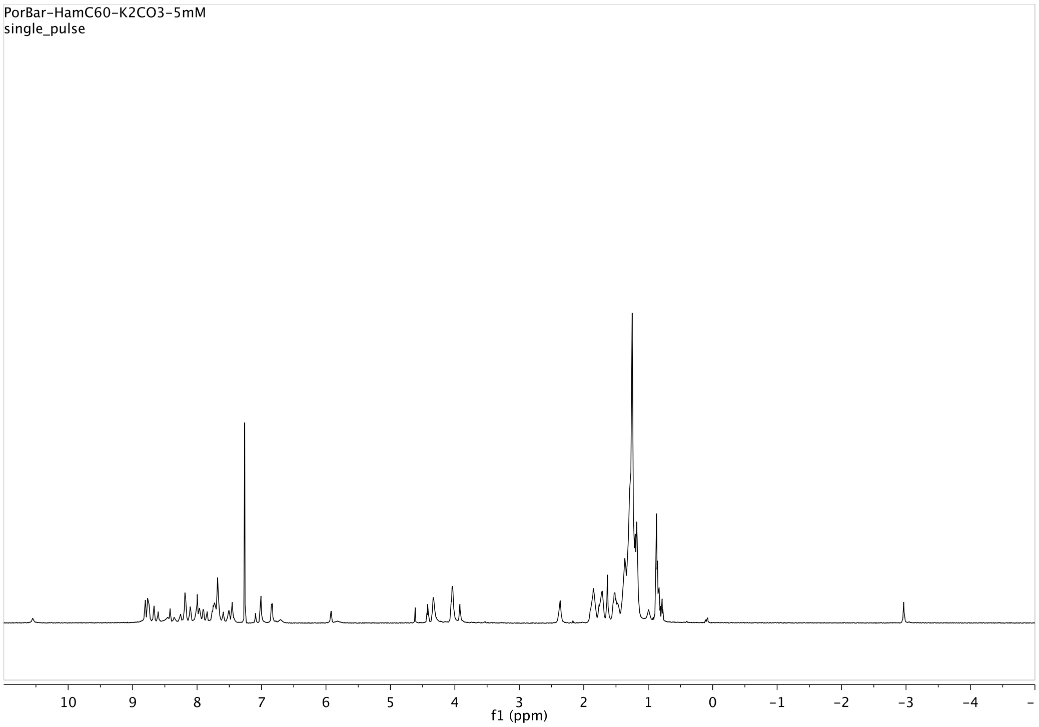

Supplementary Figure 14. ^{1}H NMR spectrum of a mixture of **2** (5.0×10^{-3} mol L^{-1}) and **3** (5.0×10^{-3} mol L^{-1}) in chloroform-d_1 at 25 °C.

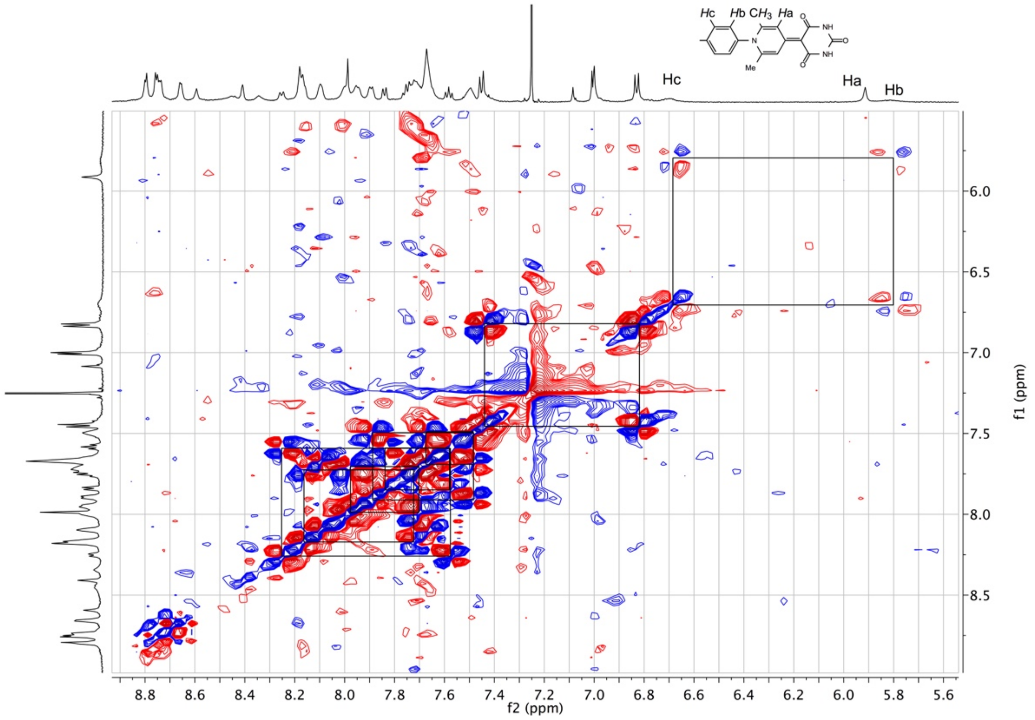

Supplementary Figure 15. DQF-COSY spectrum of a mixture of **2** (5.0×10^{-3} mol L^{-1}) and **3** (5.0×10^{-3} mol L^{-1}) in chloroform-d_1 at 25 °C.

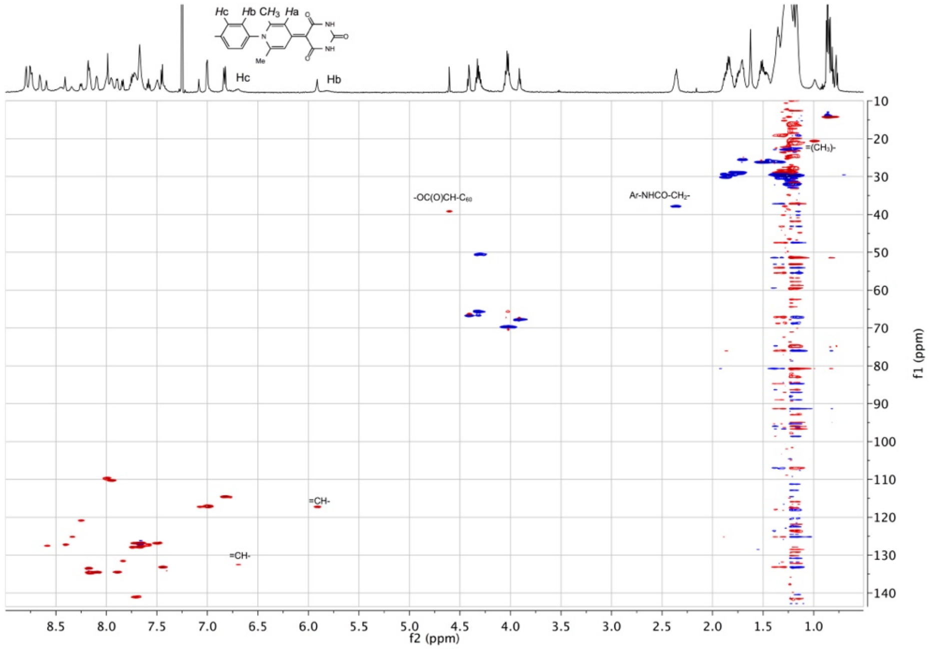

Supplementary Figure 16. HSQC spectrum of a mixture of **2** and **3** in chloroform-d_1. CH_3 and CH peaks are phased up (blue), and CH_2 peaks are phased down (red).

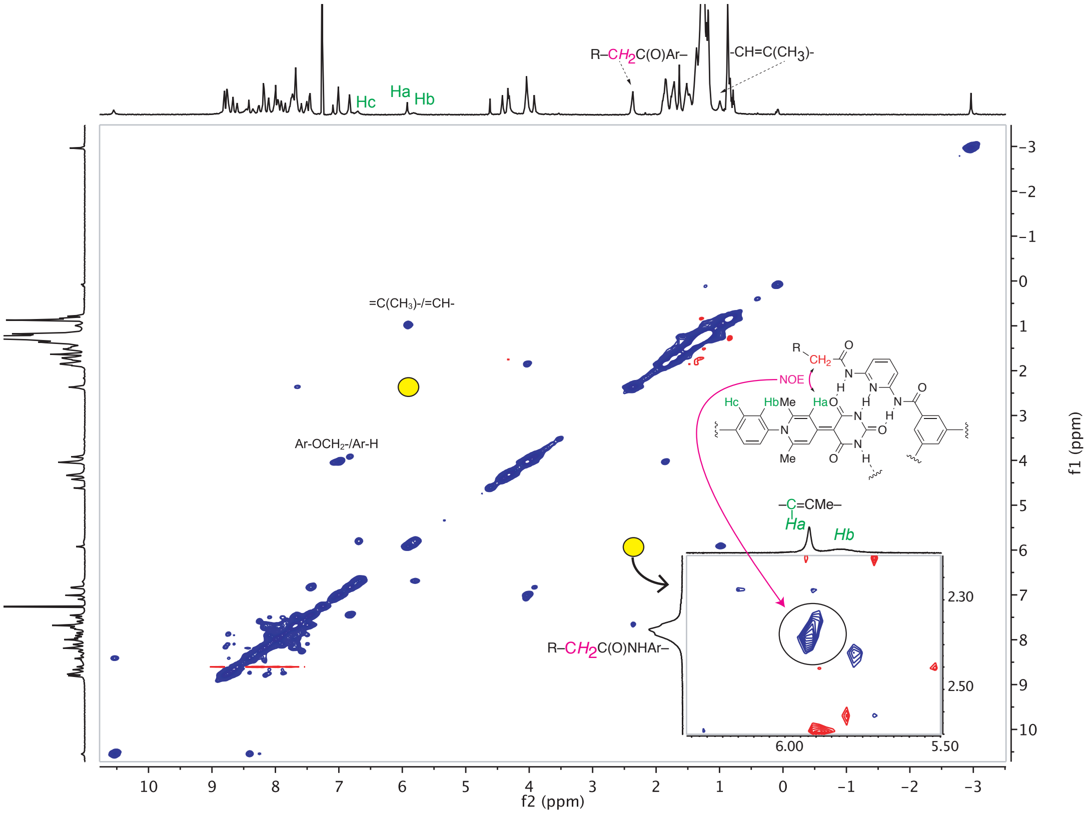

Supplementary Figure 17. NOESY spectrum of a mixture of **2** (5.0×10^{-3} mol L^{-1}) and **3** (5.0×10^{-3} mol L^{-1}) in chloroform-d_1 at 25 °C.

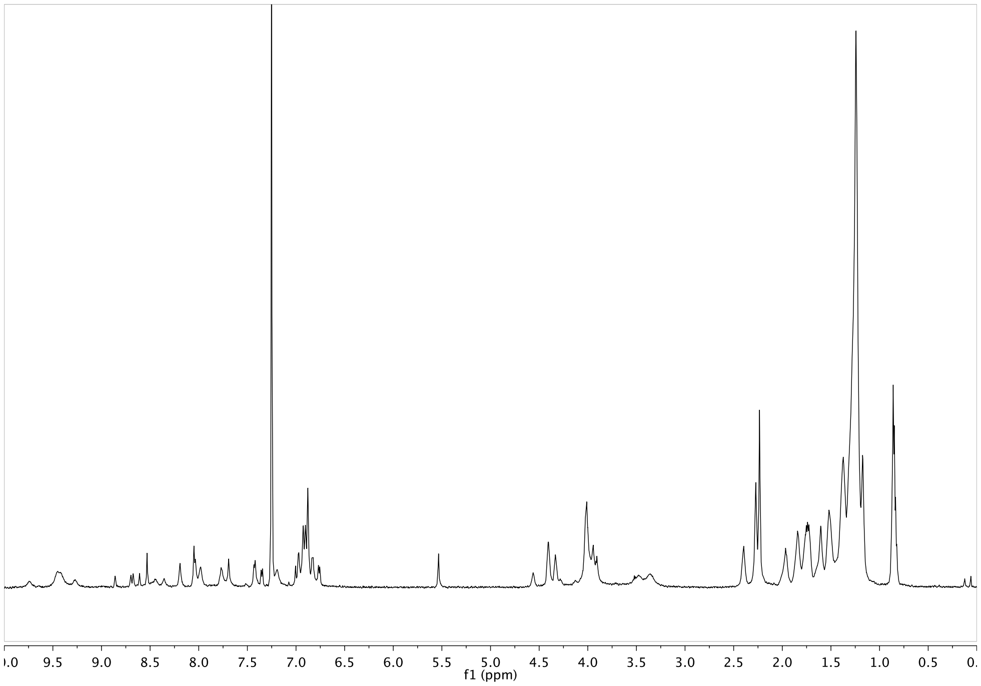

Supplementary Figure 18. ^{1}H NMR spectrum of a mixture of **3** (5.0×10^{-3} mol L^{-1}) and **1** (5.0×10^{-3} mol L^{-1}) in chloroform-d_1 at 25 °C.

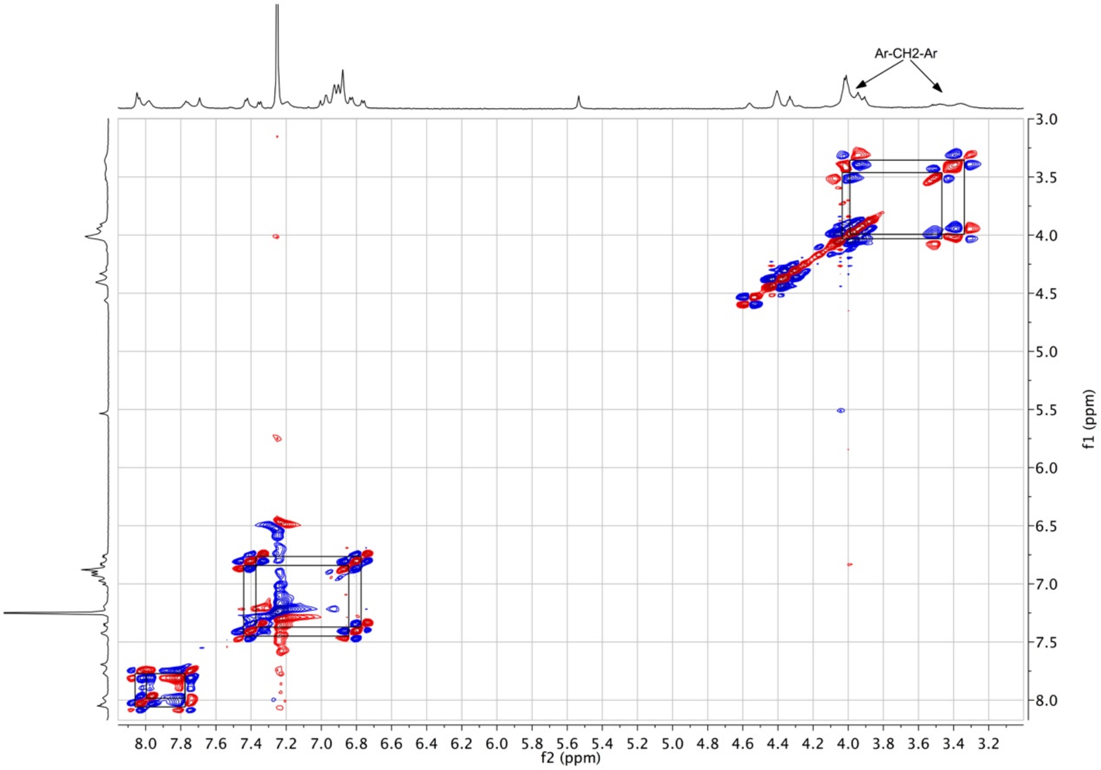

Supplementary Figure 19. DQF-COSY spectrum of a mixture of **3** (5.0×10^{-3} mol L^{-1}) and **1** (5.0×10^{-3} mol L^{-1}) in chloroform-d_1 at 25 °C.

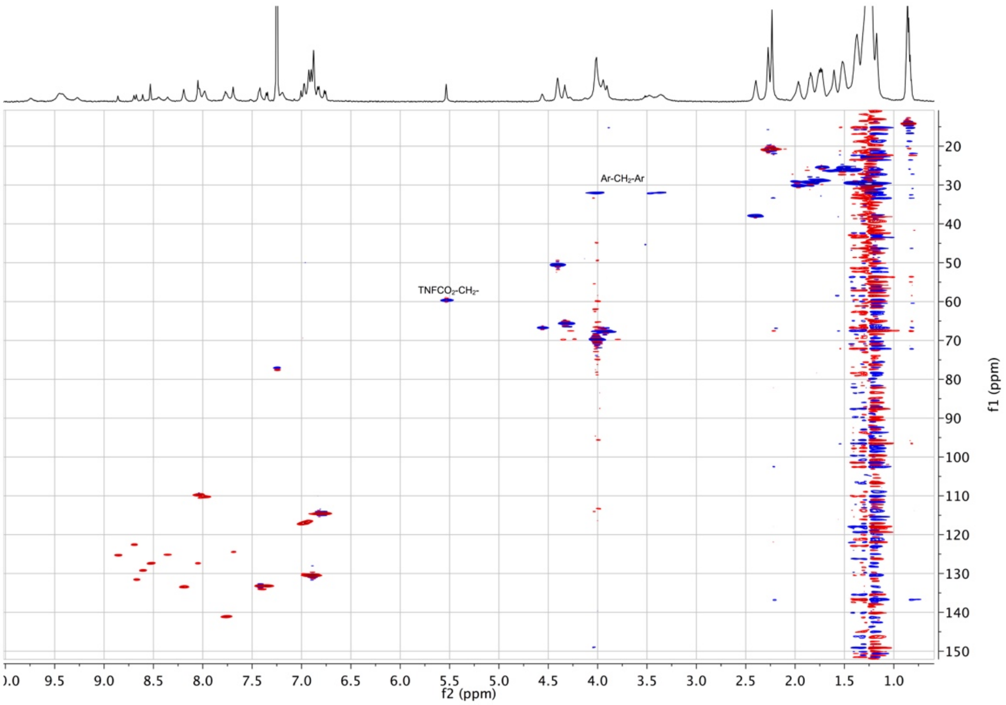

Supplementary Figure 20. HSQC spectrum of a mixture of **3** (5.0×10^{-3} mol L^{-1}) and **1** (5.0×10^{-3} mol L^{-1}) in chloroform-d_1 at 25 °C. CH_3 and CH peaks are phased up (red), and CH_2 peaks are phased down (blue).

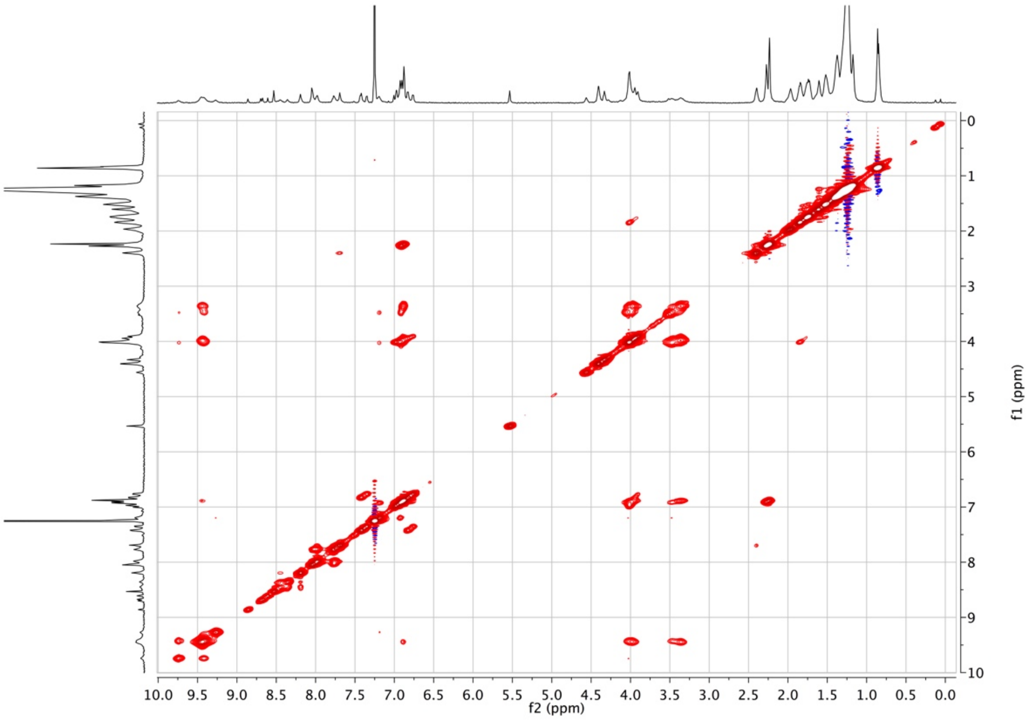

Supplementary Figure 21. NOESY spectrum of a mixture of **3** (5.0×10^{-3} mol L^{-1}) and **1** (5.0×10^{-3} mol L^{-1}) in chloroform-d_1 at 25 °C.

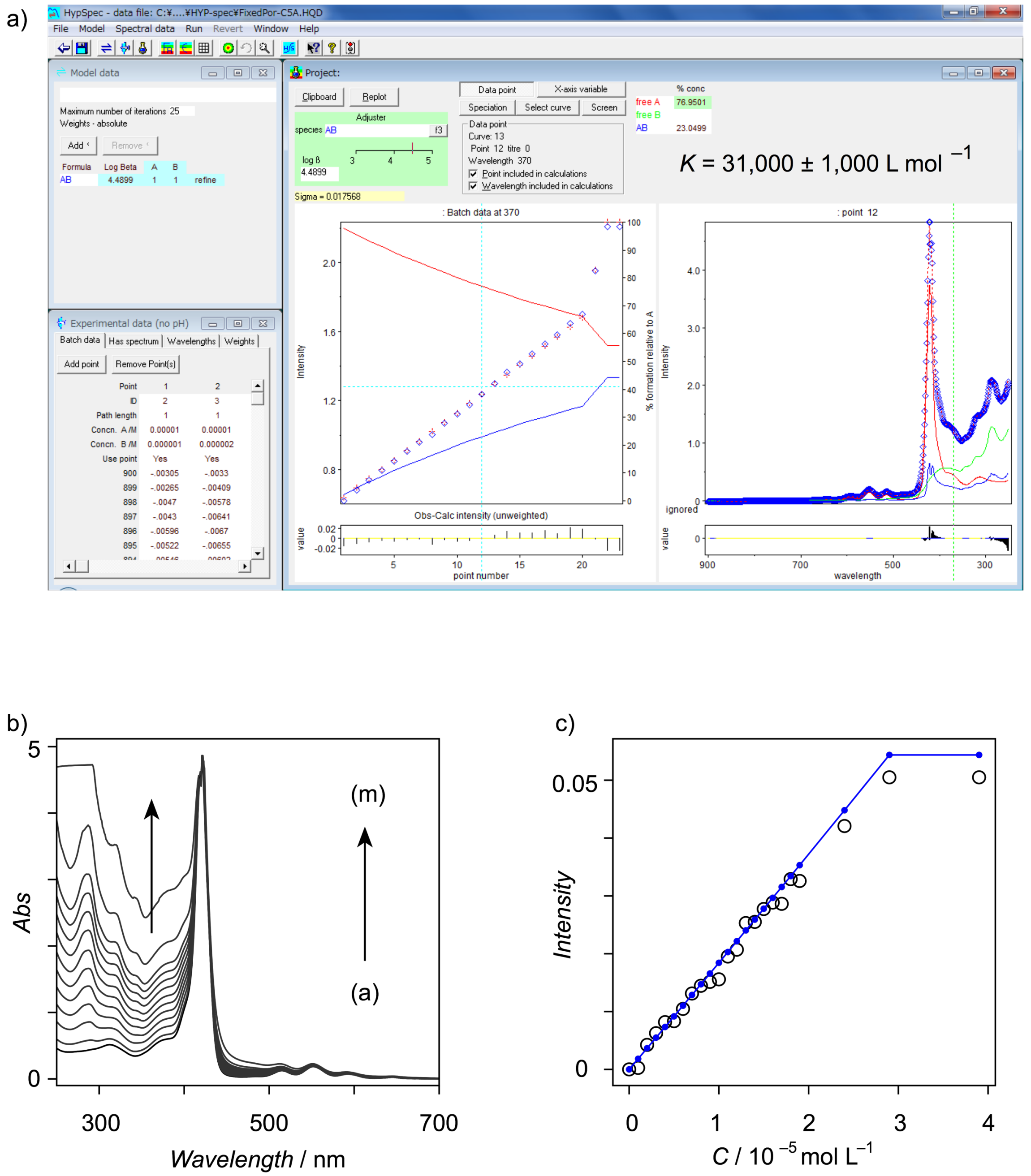

Supplementary Figure 22. Determination of an association constant between **1** and **2** in 1,2-dichloroethane at 25 °C by using (a) HypSpec software. (b) UV-vis absorption changes of **2** (1.0×10^{-5} mol L^{-1}) upon the addition of **1** and (c) the fitting curve at 370 nm. Concentrations of **1** are (a-m) 0.0, 0.1, 0.3, 0.5, 0.7, 0.9, 1.1, 1.3, 1.5, 1.7, 1.9, 2.5, 4.0 × 10^{-5} mol L^{-1}.

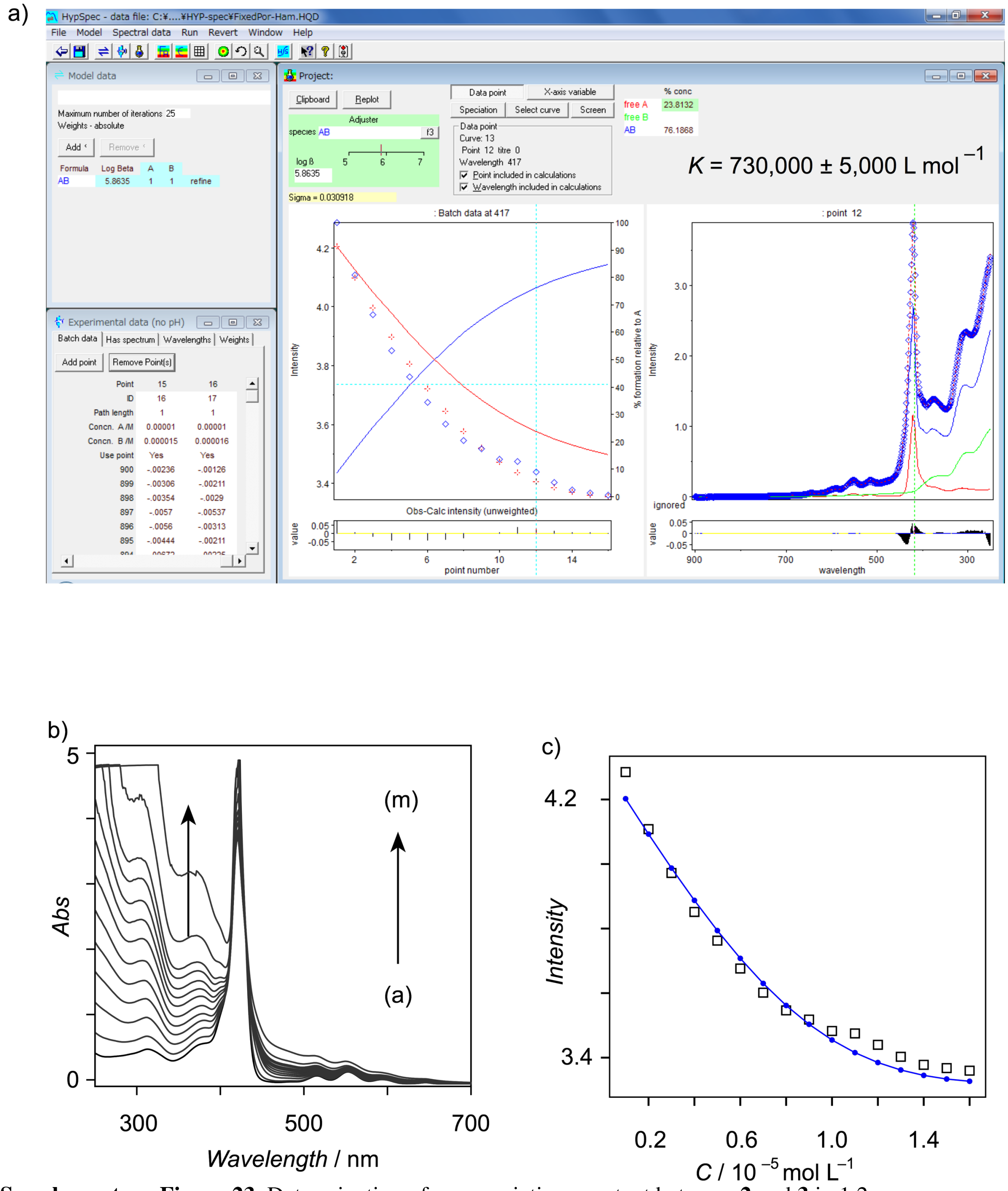

Supplementary Figure 23. Determination of an association constant between **2** and **3** in 1,2-dichloroethane at 25 °C by using (a) HypSpec software. (b) UV-vis absorption changes of **2** (1.0×10^{-5} mol L^{-1}) upon the addition of **3** and (c) the fitting curve at 417 nm. Concentrations of **3** are (a-m) 0.0, 0.1, 0.3, 0.5, 0.7, 0.9, 1.1, 1.3, 1.5, 1.7, 1.9, 2.5, 4.0 × 10^{-5} mol L^{-1}.

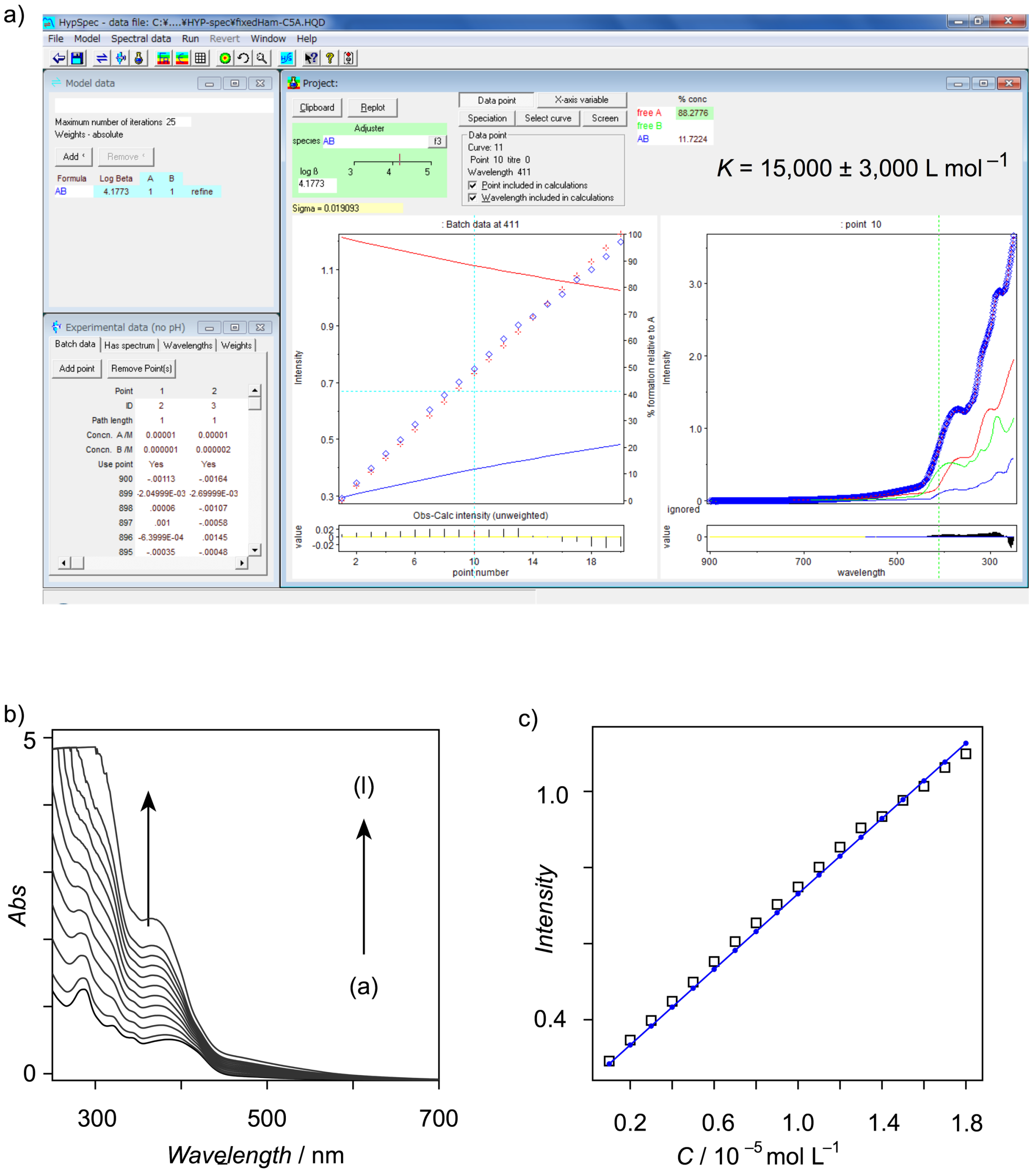

Supplementary Figure 24. Determination of an association constant between **3** and **1** in 1,2-dichloroethane at 25 °C by using (a) HypSpec software. (b) UV-vis absorption changes of **1** (1.0×10^{-5} mol L^{-1}) upon the addition of **3** and (c) the fitting curve at 411 nm. Concentrations of **3** are (a-l) 0.0, 0.1, 0.3, 0.5, 0.7, 0.9, 1.1, 1.3, 1.5, 1.7, 1.9, 2.5×10^{-5} mol L^{-1}.

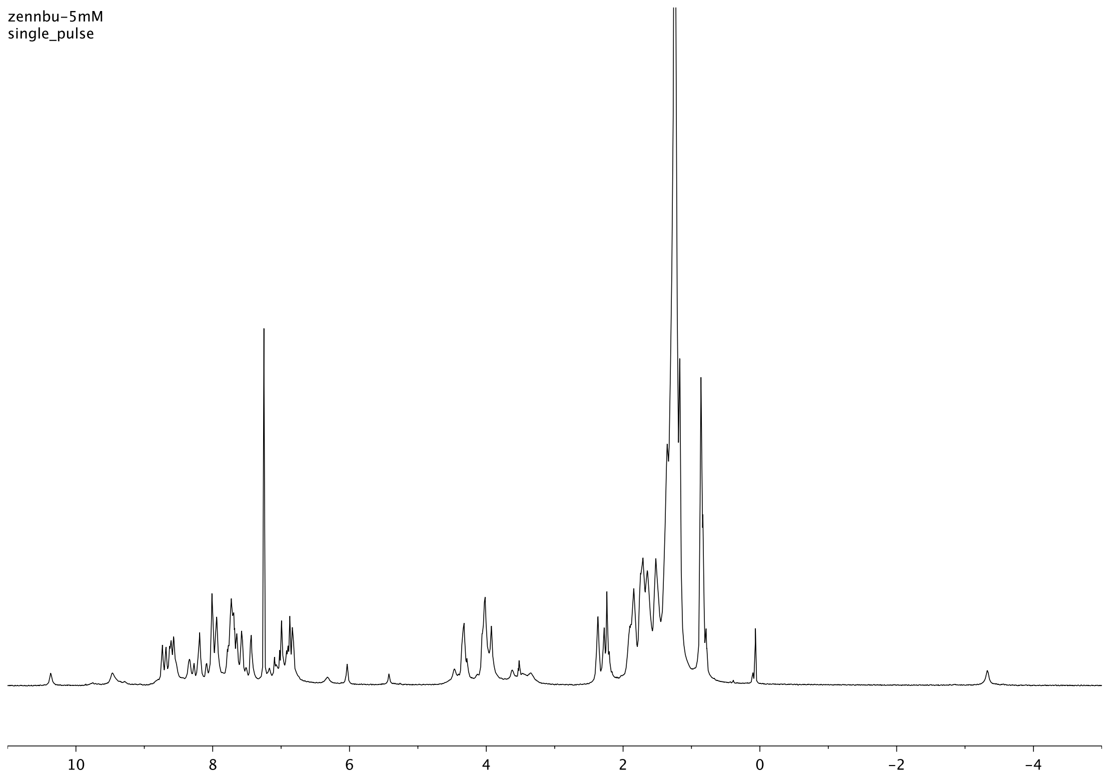

Supplementary Figure 25. ^{1}H NMR spectrum of a 1:1:1 mixture of **1** (5.0 x 10^{-3} mol L^{-1}), **2** (5.0 x 10^{-3} mol L^{-1}) and **3** (5.0 x 10^{-3} mol L^{-1}) in chloroform-d_1 at 25 °C.

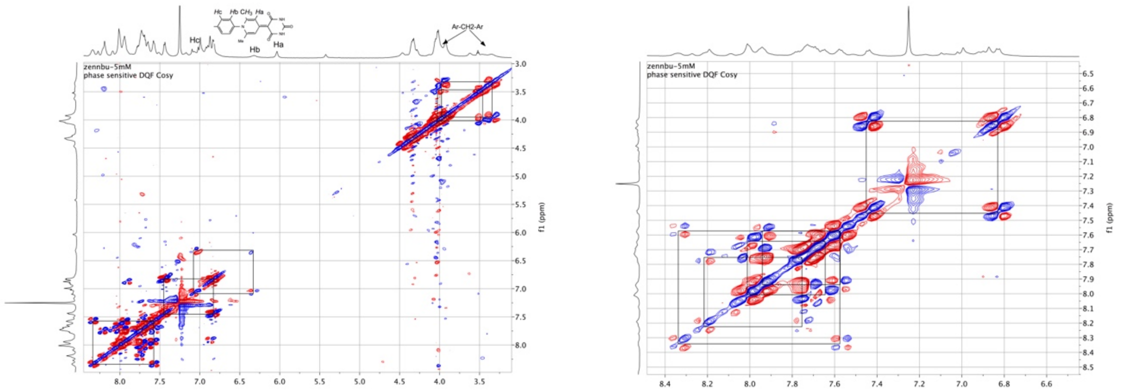

Supplementary Figure 26. DQF-COSY spectrum of a 1:1:1 mixture of **1** (5.0 x 10^{-3} mol L^{-1}), **2** (5.0 x 10^{-3} mol L^{-1}) and **3** (5.0 x 10^{-3} mol L^{-1}) in chloroform-d_1 at 25 °C.

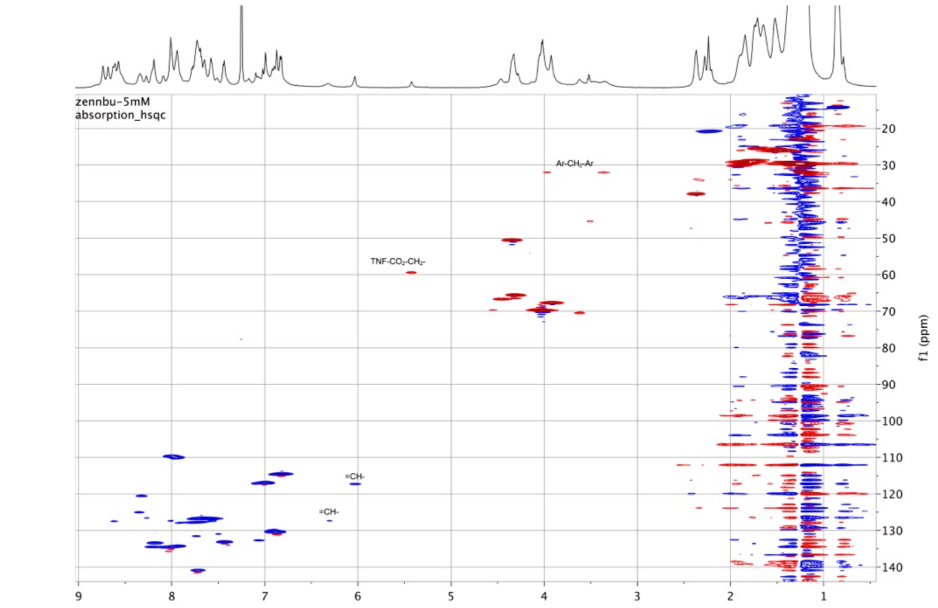

Supplementary Figure 27. HSQC spectrum of a 1:1:1 mixture of **1** (5.0×10^{-3} mol L^{-1}), **2** (5.0×10^{-3} mol L^{-1}) and **3** (5.0×10^{-3} mol L^{-1}) in chloroform-d_1 at 25 °C. CH_3 and CH peaks are phased up (blue), and CH_2 peaks are phased down (red).

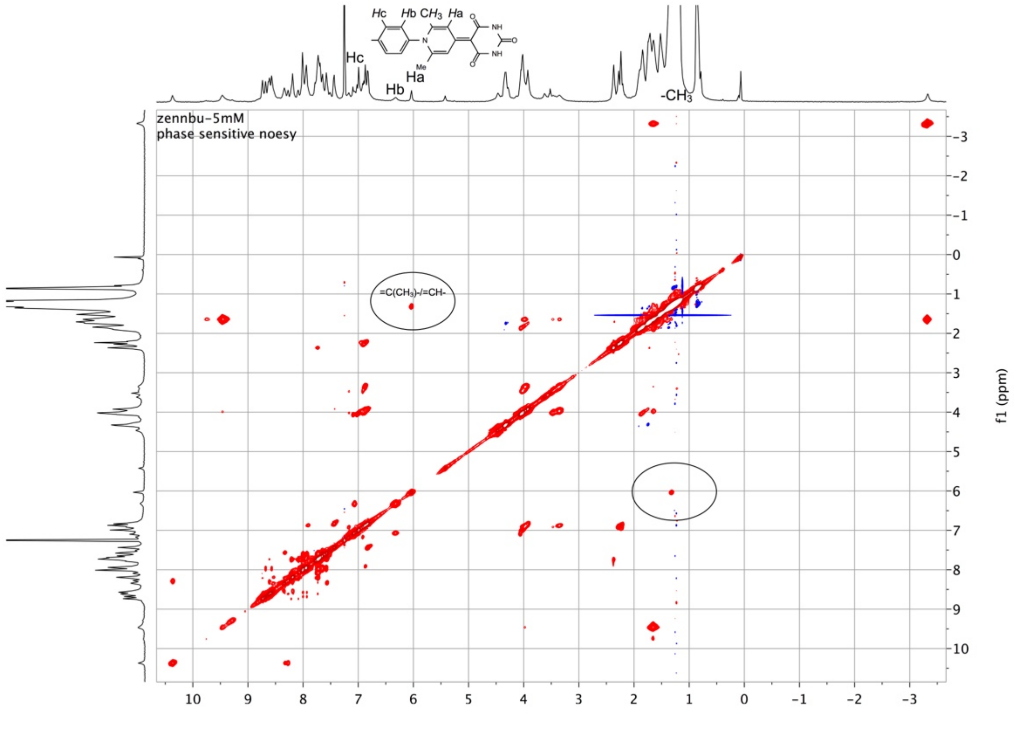

Supplementary Figure 28. NOESY spectrum of a 1:1:1 mixture of **1** (5.0×10^{-3} mol L^{-1}), **2** (5.0×10^{-3} mol L^{-1}) and **3** (5.0×10^{-3} mol L^{-1}) in chloroform-d_1 at 25 °C.

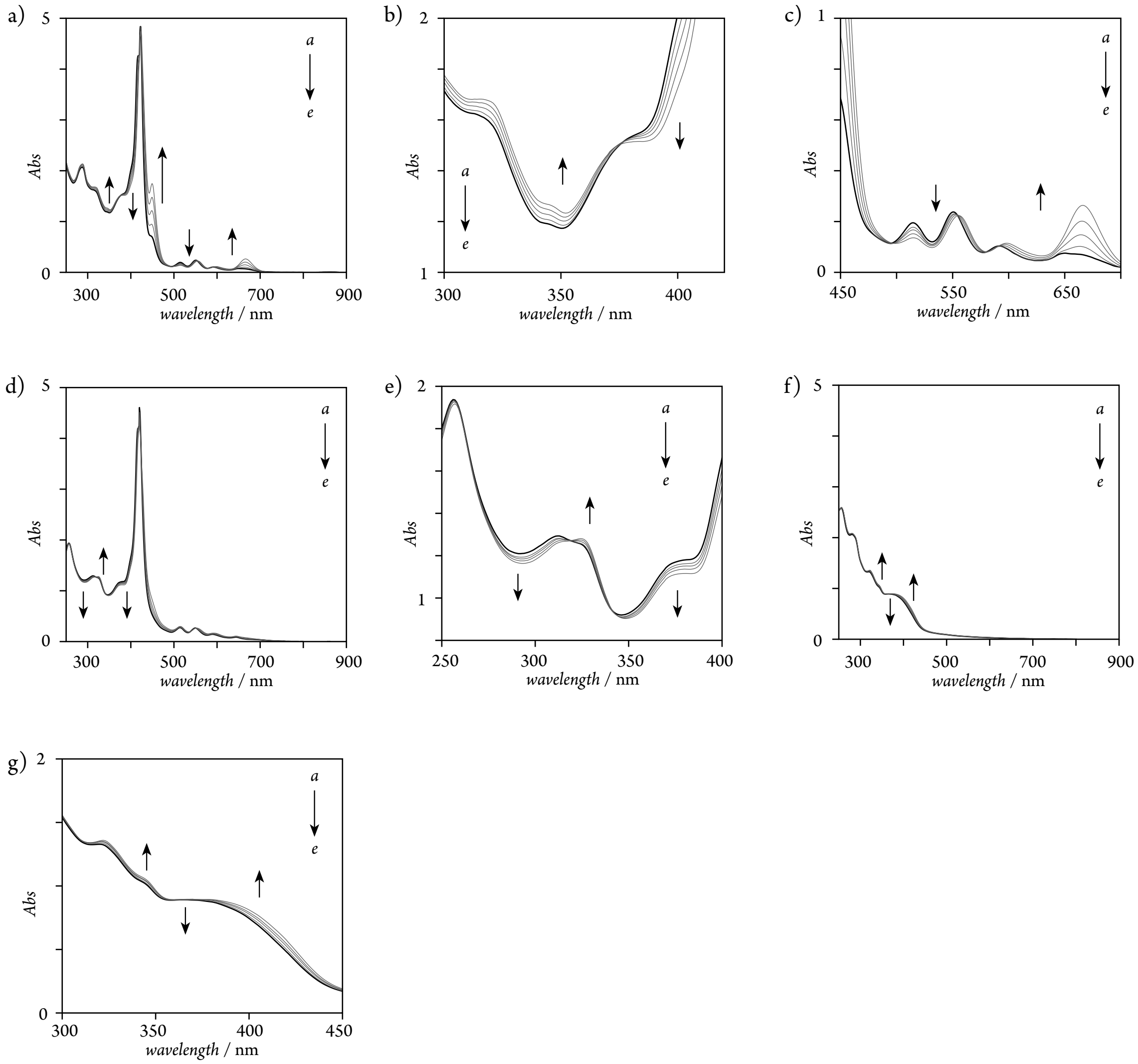

Supplementary Figure 29. Variable temperature UV-vis spectra of (a-c) a 1:1 mixture of **1** (1.0×10^{-5} mol L^{-1}) and **2** (1.0×10^{-5} mol L^{-1}), (d-e) a 1:1 mixture of **2** (1.0×10^{-5} mol L^{-1}) and **3** (1.0×10^{-5} mol L^{-1}), and (f-g) a 1:1 mixture of **3** (1.0×10^{-5} mol L^{-1}) and **1** (1.0×10^{-5} mol L^{-1}) in 1,2-dichloroethane (from a to e: 40, 30, 20, 10, 0 °C).

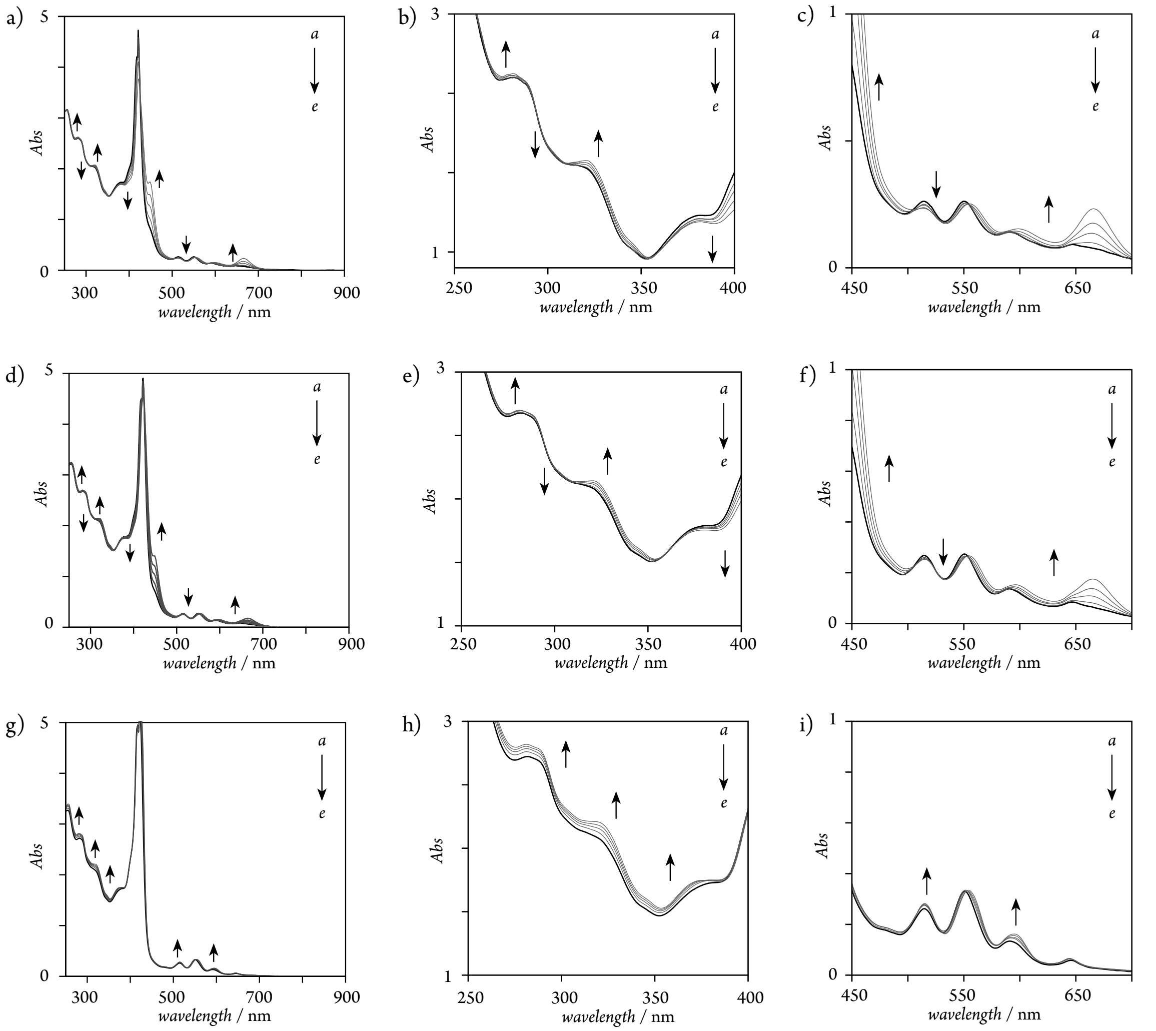

Supplementary Figure 30. Variable temperature UV-vis absorption spectra of (a-c) a 1:1:1 mixture of **1** (1.0×10^{-5} mol L^{-1}), **2** (1.0 x 10^{-5} mol L^{-1}) and **3** (1.0×10^{-5} mol L^{-1}), (d-f) a linear combination of the UV-vis absorption spectra of 1:1 mixtures of **1** (1.0×10^{-5} mol L^{-1}) and **2** (1.0×10^{-5} mol L^{-1}), **2** (1.0×10^{-5} mol L^{-1}) and **3** (1.0×10^{-5} mol L^{-1}), and **3** (1.0×10^{-5} mol L^{-1}) and **1** (1.0×10^{-5} mol L^{-1}), and (g-i) a linear combination of the UV-vis absorption spectra of **1** (1.0×10^{-5} mol L^{-1}), **2** (1.0×10^{-5} mol L^{-1}), and **3** (1.0×10^{-5} mol L^{-1}) in 1,2-dichloroethane (from a to e: 40, 30, 20, 10, 0 °C).

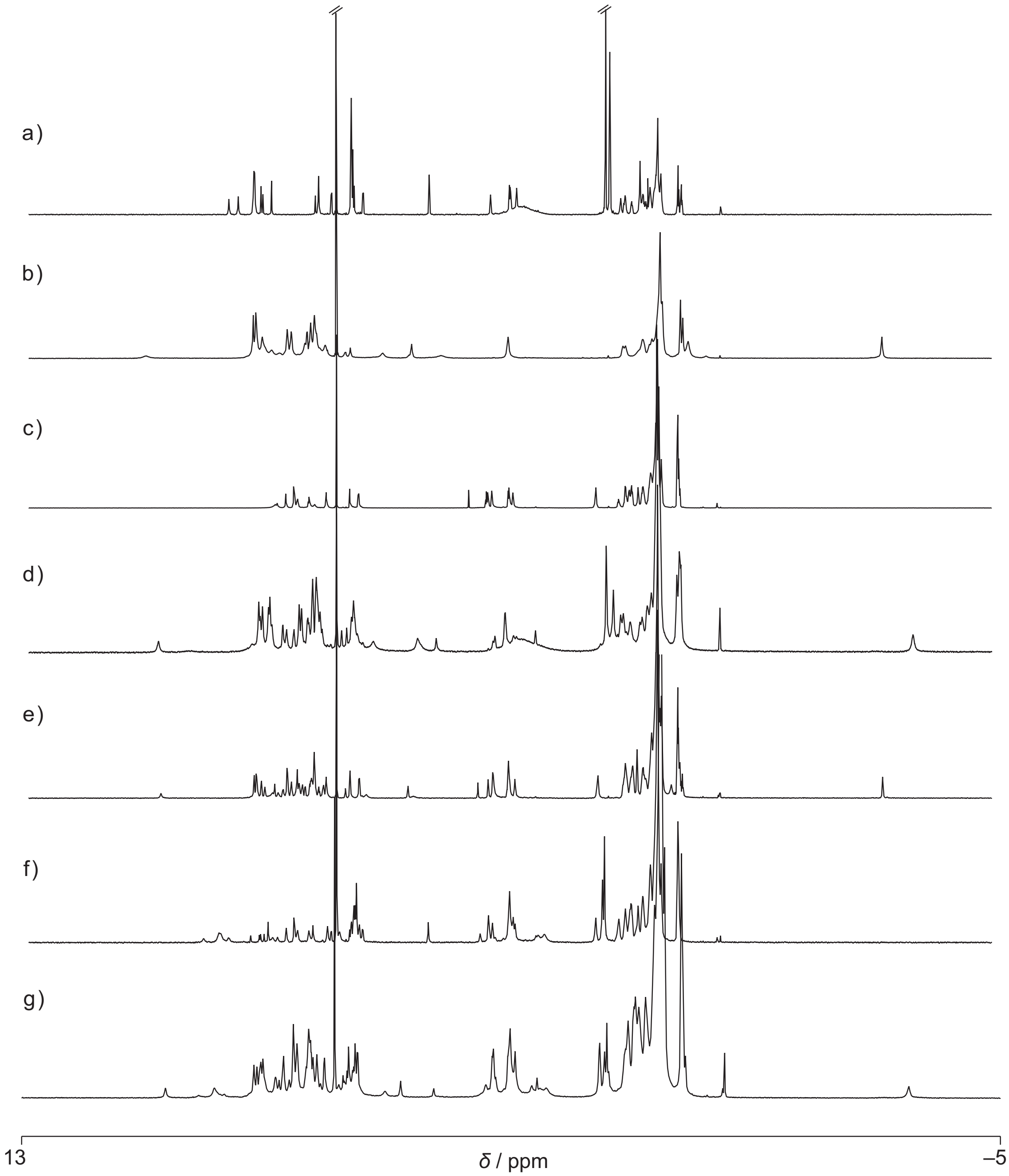

Supplementary Figure 31. ^{1}H NMR spectra of (a) **1** (5.0×10^{-3} mol L^{-1}), (b) **2** (5.0×10^{-3} mol L^{-1}), (c) **3** (5.0×10^{-3} mol L^{-1}), (d) a 1:1 mixture of **1** (5.0×10^{-3} mol L^{-1}) and **2** (5.0×10^{-3} mol L^{-1}), (e) a 1:1 mixture of **2** (5.0×10^{-3} mol L^{-1}) and **3** (5.0×10^{-3} mol L^{-1}), (f) a 1:1 mixture of **3** (5.0×10^{-3} mol L^{-1}) and **1** (5.0×10^{-3} mol L^{-1}), and (g) a 1:1:1 mixture of **1** (5.0×10^{-3} mol L^{-1}), **2** (5.0×10^{-3} mol L^{-1}) and **3** (5.0×10^{-3} mol L^{-1}) in chloroform-d_1 at 25°C.

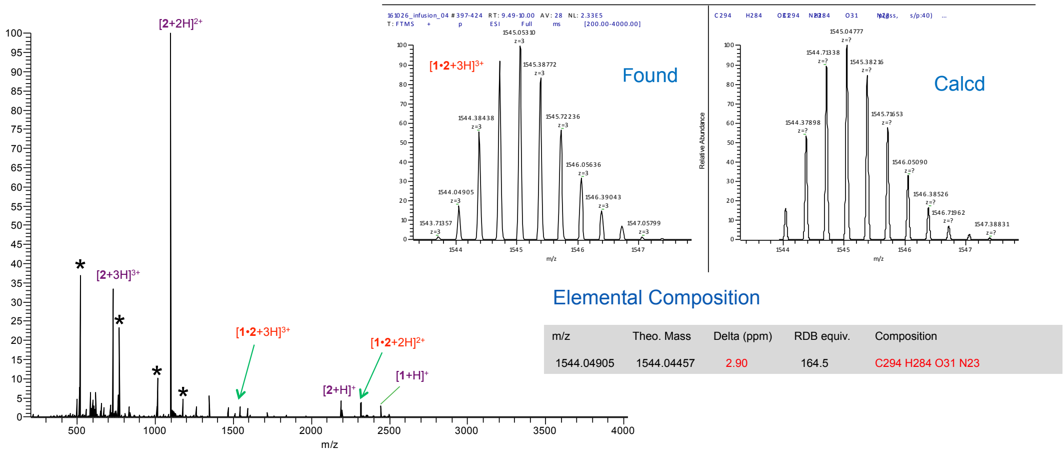

Supplementary Figure 32. ESI-Orbitrap-MS spectrum of heterodimer **1•2** in a mixture of **1** and **2**. Found and calculated isotope patterns of $[\mathbf{1{\cdot}2} + 3H]^{3+}$. *: undefined contamination.

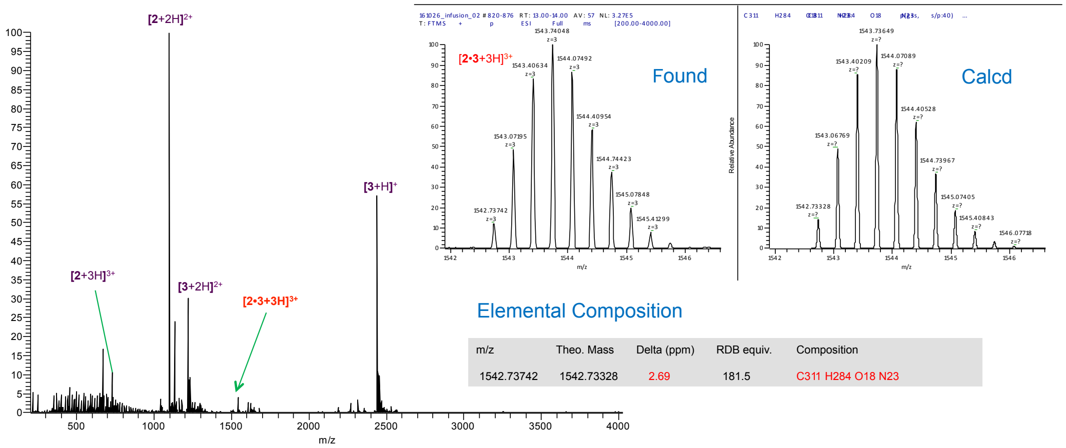

Supplementary Figure 33. ESI-Orbitrap-MS spectrum of heterodimer **2•3** in a mixture of **2** and **3**. Found and calculated isotope patterns of $[\mathbf{2{\cdot}3} + 3H]^{3+}$.

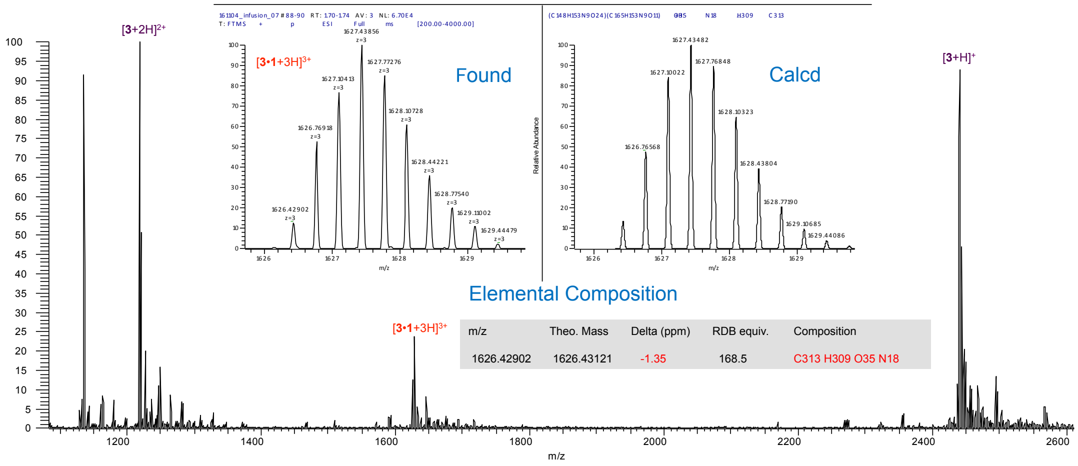

Supplementary Figure 34. ESI-Orbitrap-MS spectrum of heterodimer **3•1** in a mixture of **3** and **1**. Found and calculated isotope patterns of $[\mathbf{3{\cdot}1} + 3H]^{3+}$.

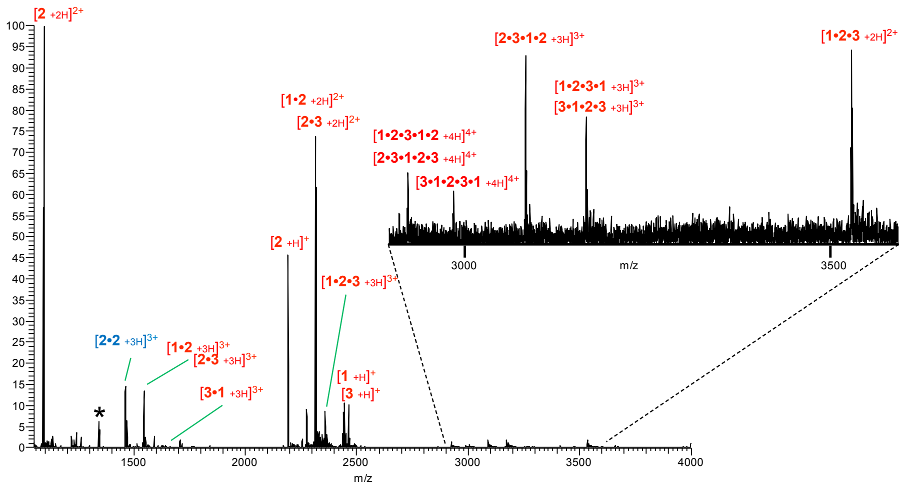

Supplementary Figure 35. ESI-Orbitrap-MS full and expanded spectra of a mixture of **1**, **2**, and **3** ([**1**] = [**2**] = [**3**] = 0.09 mmol L^{-1}) in chloroform. *: undefined contamination.

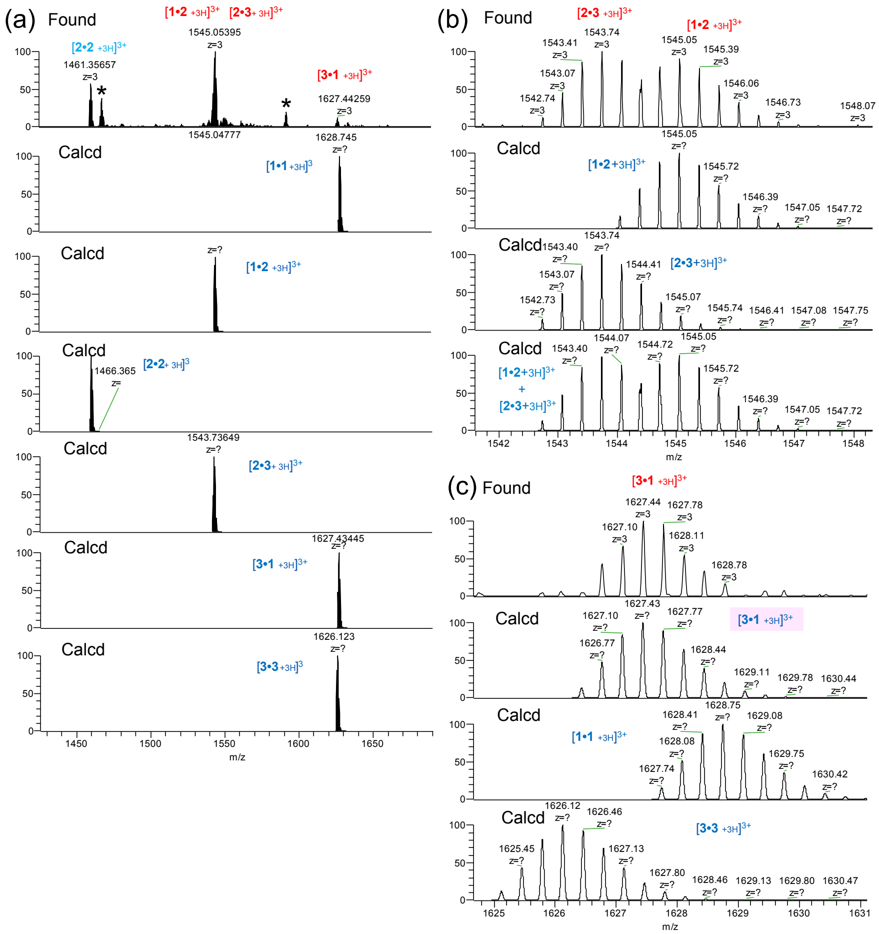

Supplementary Figure 36. (a) Comparison of observed molecular ions [**1•2** + 3H]$^{3+}$, [**2•3** + 3H]$^{3+}$, and [**3•1** + 3H]$^{3+}$ in Supplementary Figure 35 and calculated isotope patterns of [**1•1** + 3H]$^{3+}$, [**1•2** + 3H]$^{3+}$, [**2•2** + 3H]$^{3+}$, [**2•3** + 3H]$^{3+}$, [**3•1** + 3H]$^{3+}$, [**3•3** + 3H]$^{3+}$. *: undefined contamination. (b,c) Expanded MS spectra of spectra (a).

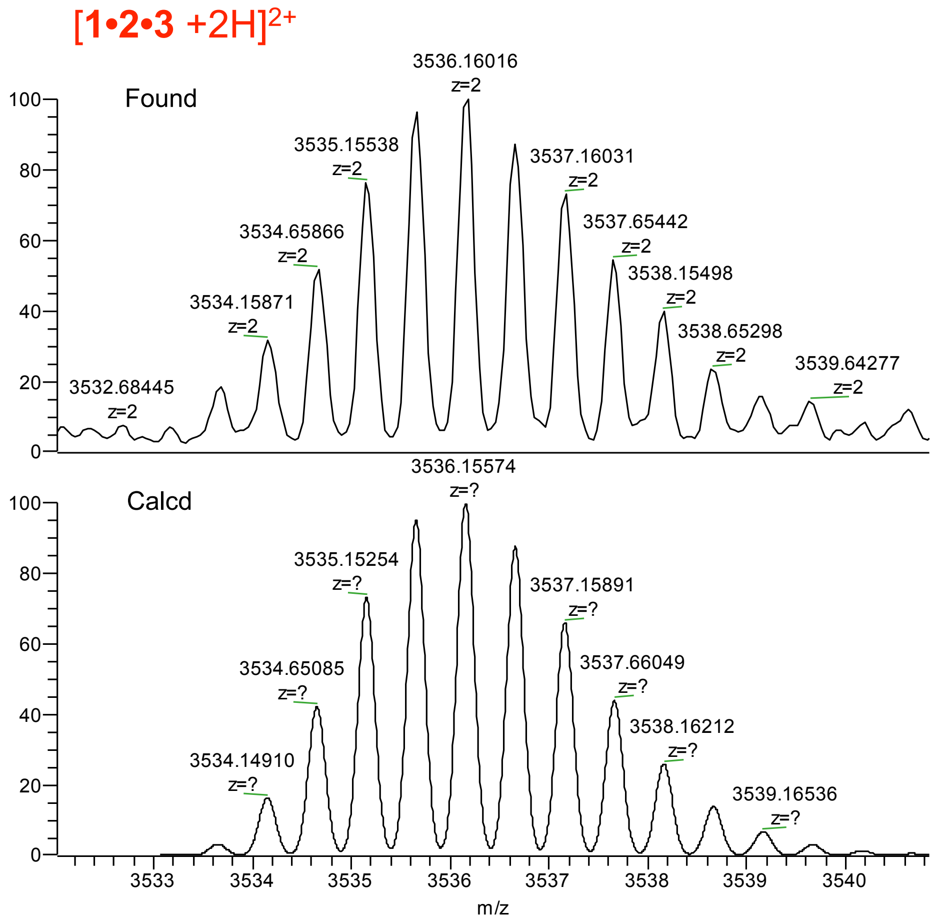

Supplementary Figure 37. Found and calculated (resolving at 18000) isotope patterns of [**1**•**2**•**3** + 2H]$^{2+}$.

Supplementary Table 1. Selected mass information in [**1**•**2**•**3** + 2H]$^{2+}$. (red: the most abundant peak)

Found (m/z)	Calcd	difference (Found−Calcd)	error (ppm)
3534.15871	3534.14910	0.00961	2.72
3534.65866	3534.65085	0.00781	2.21
3535.15538	3535.15254	0.00284	0.80
3535.65364	3535.65419	−0.00055	−0.16
3536.16016	3536.15574	0.00442	1.25
3536.65819	3536.65598	0.00221	0.62
3537.16031	3537.15891	0.00140	0.40
3537.65442	3537.66049	−0.00607	−1.72
3538.15498	3538.16212	−0.00714	−2.02

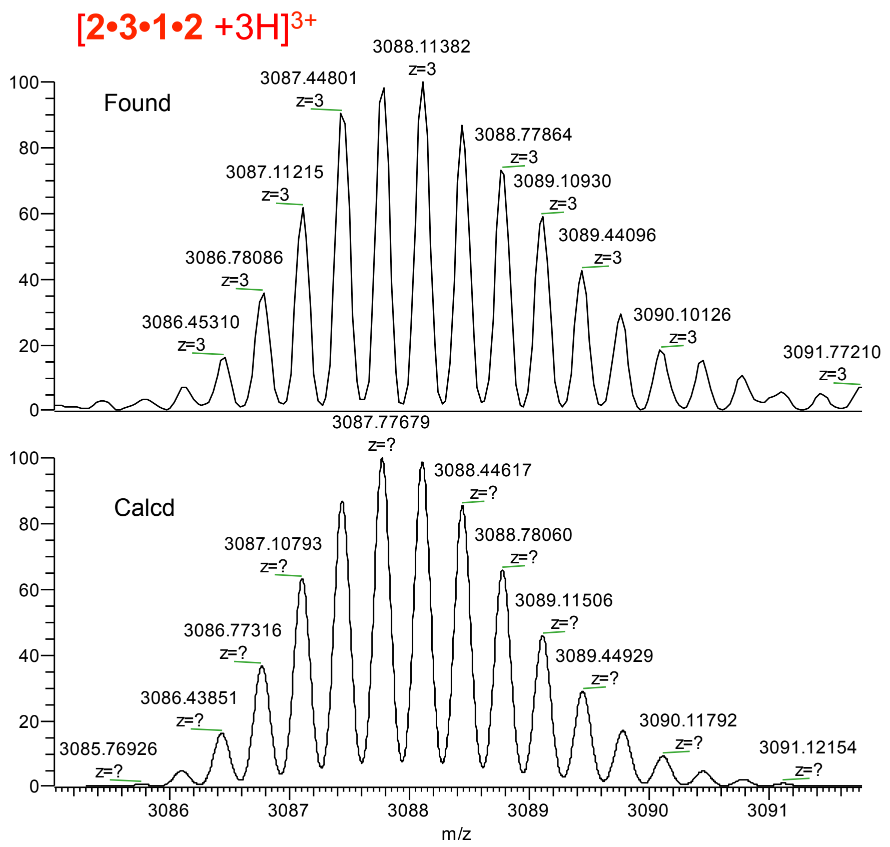

Supplementary Figure 38. Found and calculated (resolving at 20000) isotope patterns of [**2**•**3**•**1**•**2** + 3H]$^{2+}$.

Supplementary Table 2. Selected mass information in [**2**•**3**•**1**•**2** + 3H]$^{3+}$. (red: the most abundant peak)

Found (m/z)	Calcd	difference (Found−Calcd)	error (ppm)
3086.78086	3086.77316	0.00770	2.49
3087.11215	3087.10793	0.00422	1.37
3087.44801	3087.44245	0.00556	1.80
3087.78128	3087.77679	0.00449	1.45
3088.11382	3088.11145	0.00237	0.77
3088.44732	3088.44617	0.00115	0.37
3088.77864	3088.78060	−0.00196	−0.63
3089.10930	3089.11506	−0.00576	−1.86
3089.44096	3089.44929	−0.00833	−2.70

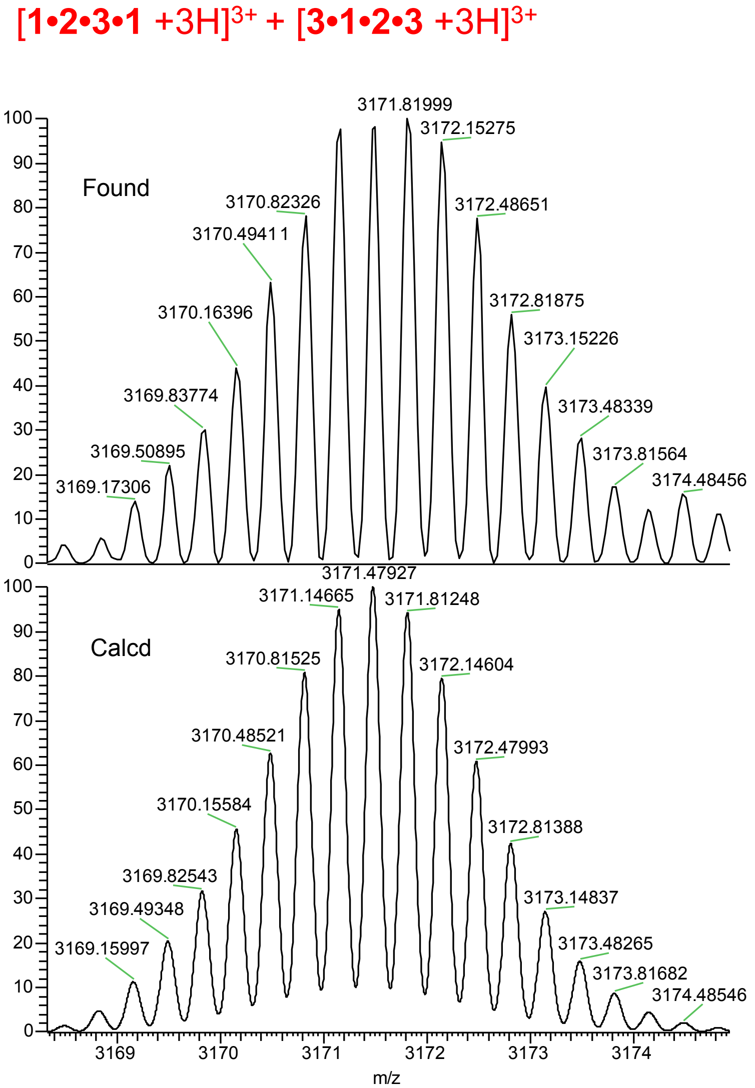

Supplementary Figure 39. Found and calculated (resolving at 20000) isotope patterns of [**1**·**2**·**3**·**1** + 3H]$^{2+}$ + [**3**·**1**·**2**·**3** + 3H]$^{2+}$.

Supplementary Table 3. Selected mass information in [**1**·**2**·**3**·**1** + 3H]$^{3+}$ + [**3**·**1**·**2**·**3** + 3H]$^{3+}$. (red: the most abundant peak)

Found (m/z)	Calcd	difference (Found–Calcd)	error (ppm)	Found (m/z)	Calcd	difference (Found–Calcd)	error (ppm)
3170.49411	3170.48521	0.00890	2.81	3172.81875	3172.81388	0.00487	1.53
3170.82326	3170.81525	0.00801	2.53	3173.15226	3173.14837	0.00389	1.23
3171.15310	3171.14665	0.00645	2.03	3173.48339	3173.48265	0.00074	0.23
3171.48717	3171.47927	0.00790	2.49	3173.81564	3173.81682	−0.00118	−0.37
3171.81999	3171.81248	0.00751	2.37	3174.14715	3174.15123	−0.00408	−1.29
3172.15275	3172.14604	0.00671	2.12	3174.48456	3174.48546	−0.00090	−0.28
3172.48651	3172.47933	0.00718	2.26				

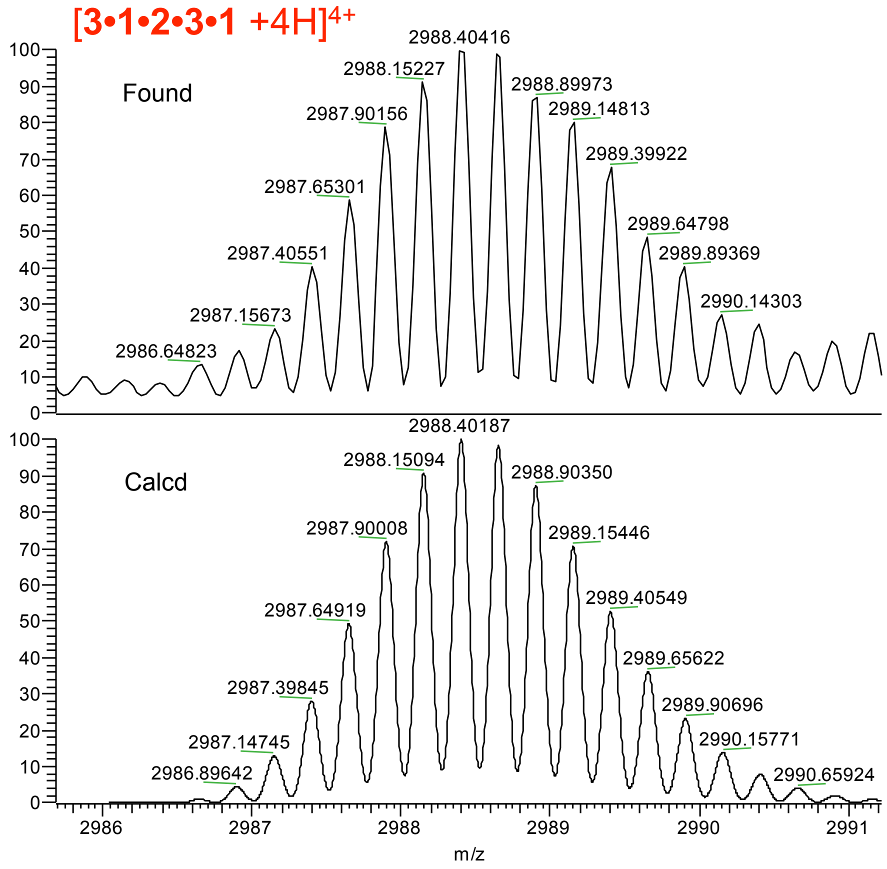

Supplementary Figure 40. Found and calculated (resolving at 25000) isotope patterns of [**3·1·2·3·1** + 4H]$^{4+}$.

Supplementary Table 4. Selected mass information in [**3·1·2·3·1** + 4H]$^{4+}$. (red: the most abundant peak)

Found (m/z)	Calcd	difference (Found−Calcd)	error (ppm)
2987.40551	2987.39845	0.00706	2.36
2987.65301	2987.64919	0.00382	1.28
2987.90156	2987.90008	0.00148	0.50
2988.15227	2988.15094	0.00133	0.45
2988.40416	2988.40187	0.00229	0.77
2988.65097	2988.65281	−0.00184	−0.62
2988.89973	2988.90350	−0.00377	−1.26
2989.14813	2989.15446	−0.00633	−2.12
2989.39922	2989.40549	−0.00627	−2.10

[1•2•3•1•2 +4H]$^{4+}$ + [2•3•1•2•3 +4H]$^{4+}$

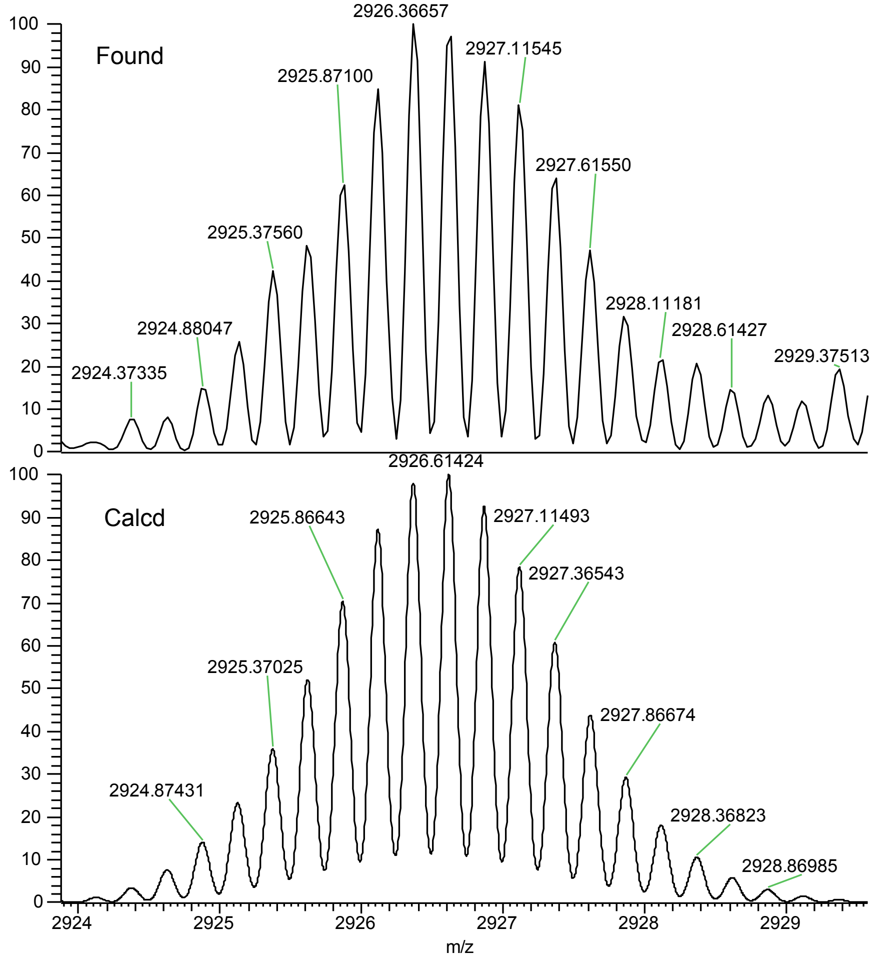

Supplementary Figure 41. Found and calculated (resolving at 24000) isotope patterns of [**1**·**2**·**3**·**1**·**2** + 4H]$^{4+}$ + [**2**·**3**·**1**·**2**·**3** + 4H]$^{4+}$.

Supplementary Table 5. Selected mass information in [**1**·**2**·**3**·**1**·**2** + 4H]$^{4+}$ + [**2**·**3**·**1**·**2**·**3** + 4H]$^{4+}$. (red: the most abundant peak)

Found (m/z)	Calcd	difference (Found−Calcd)	error (ppm)	Found (m/z)	Calcd	difference (Found−Calcd)	error (ppm)
2924.11752	2924.12236	−0.00484	−1.66	2926.86753	2926.86448	0.00305	1.04
2924.37335	2924.37368	−0.00033	−0.11	2927.11545	2927.11493	0.00052	0.18
2924.62353	2924.62455	−0.00102	−0.35	2927.36583	2927.36543	0.00040	0.14
2924.88047	2924.87431	0.00616	2.11	2927.61550	2927.61611	−0.00061	−0.21
2925.13074	2925.12256	0.00818	2.80	2927.86333	2927.86674	−0.00341	−1.16
2925.37560	2925.37025	0.00535	1.83	2928.11181	2928.11754	−0.00573	−1.96
2925.62166	2925.61815	0.00351	1.20	2928.36675	2928.36823	−0.00148	−0.51
2925.87110	2925.86643	0.00467	1.60	2928.61427	2928.61911	−0.00484	−1.65
2926.11809	2926.11526	0.00283	0.97	2928.87187	2928.86985	0.00202	0.69
2926.36657	2926.36457	0.00200	0.68	2929.11739	2929.12078	−0.00339	−1.16
2926.61652	2926.61424	0.00228	0.78	2929.37513	2929.37131	0.00382	1.30

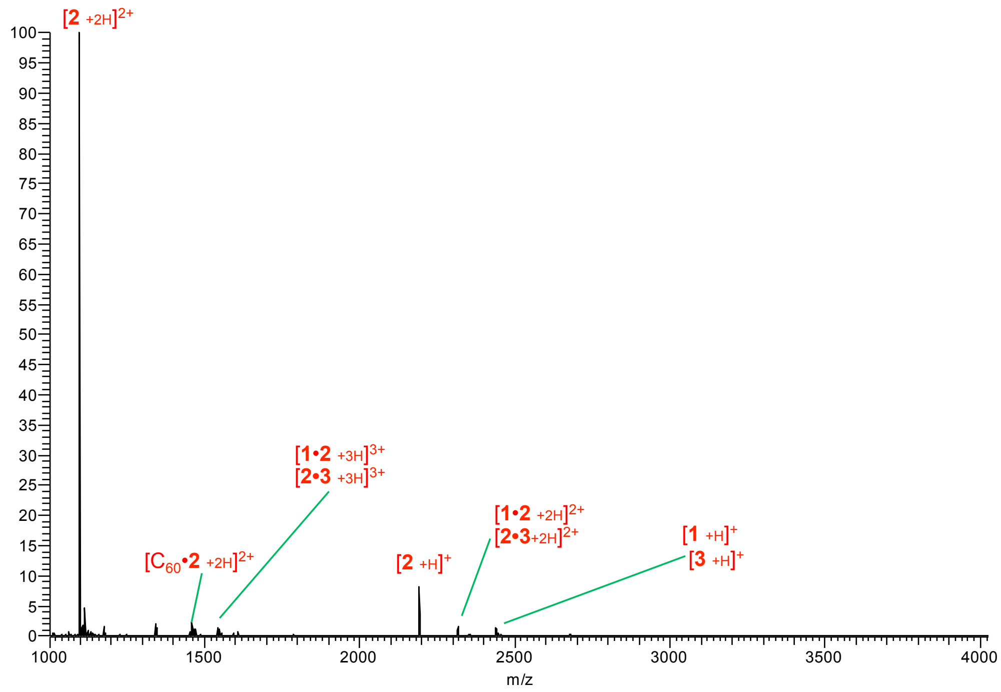

Supplementary Figure 42. End-capping experiment of supramolecular terpolymer *poly*-**1**-**2**-**3** in the presence of C_{60}. ESI-Orbitrap-MS spectrum of a mixture of **1**, **2**, **3,** and C_{60} ([**1**] = [**2**] = [**3**] = 0.07 mmol L^{-1}) in chloroform.

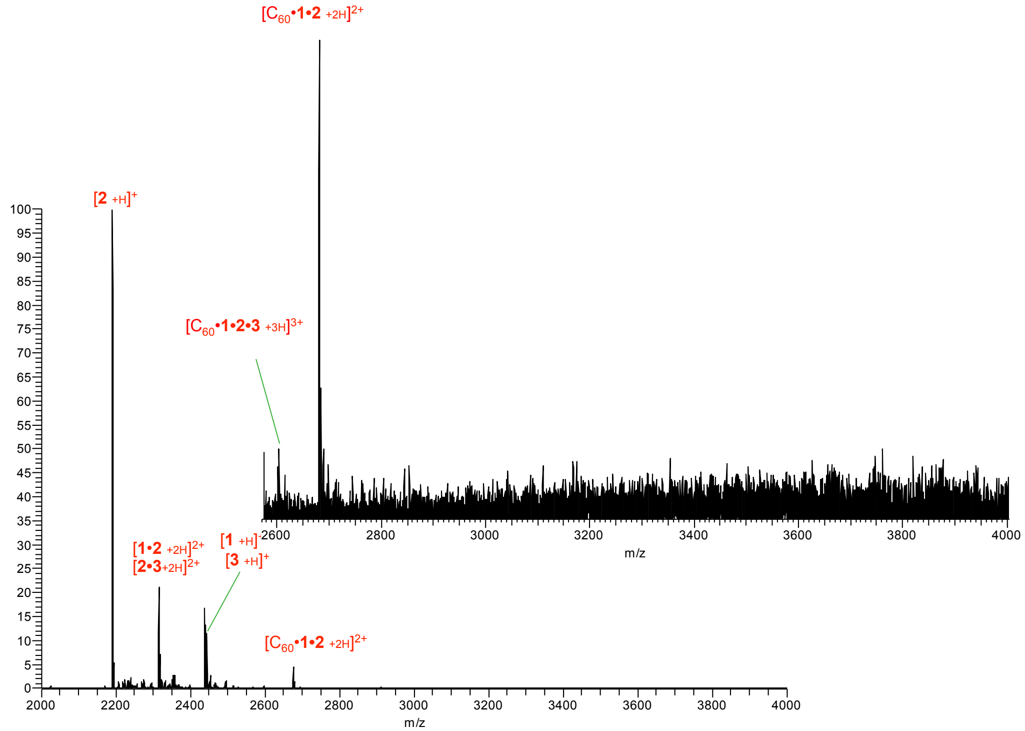

Supplementary Figure 43. Expanded spectrum of Supplementary Figure 42.

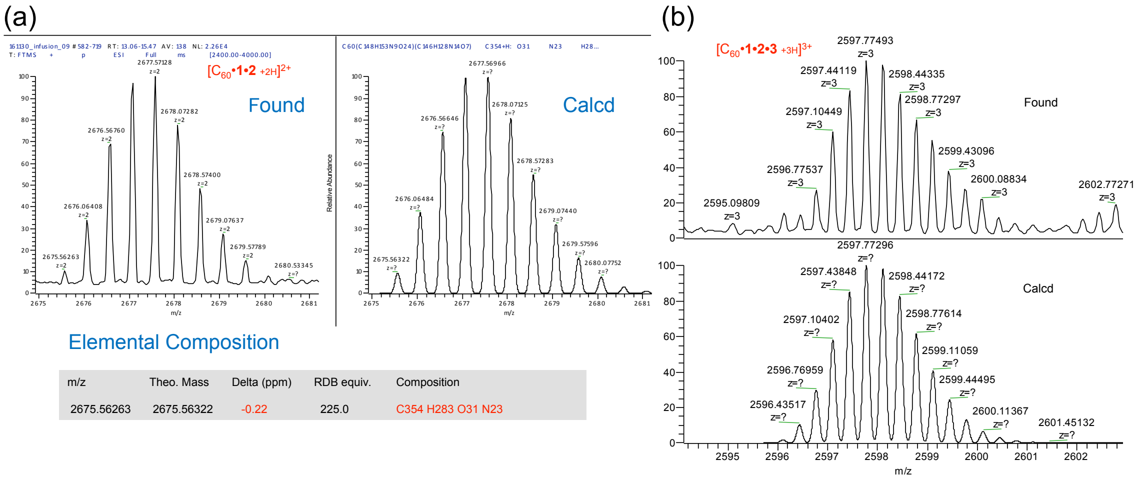

Supplementary Figure 44. Found and calculated (resolving at 20000) isotope patterns of [C_{60}•**1**•**2** + 2H]$^{2+}$ (a), and [C_{60}•**1**•**2**•**3** + 3H]$^{3+}$ (b) found in Supplementary Figure 43.

Supplementary Table 6. Selected mass information in [C_{60}•**1**•**2**•**3** + 3H]$^{3+}$. (red: the most abundant peak)

Found (m/z)	Calcd	difference (Found−Calcd)	error (ppm)
2596.77537	2596.76959	0.00578	2.23
2597.10449	2597.10402	0.00047	0.18
2597.44119	2597.43848	0.00271	1.04
2597.77493	2597.77296	0.00197	0.76
2598.10850	2598.10738	0.00112	0.43
2598.44335	2598.44172	0.00163	0.63
2598.77297	2598.77614	−0.00317	−1.22

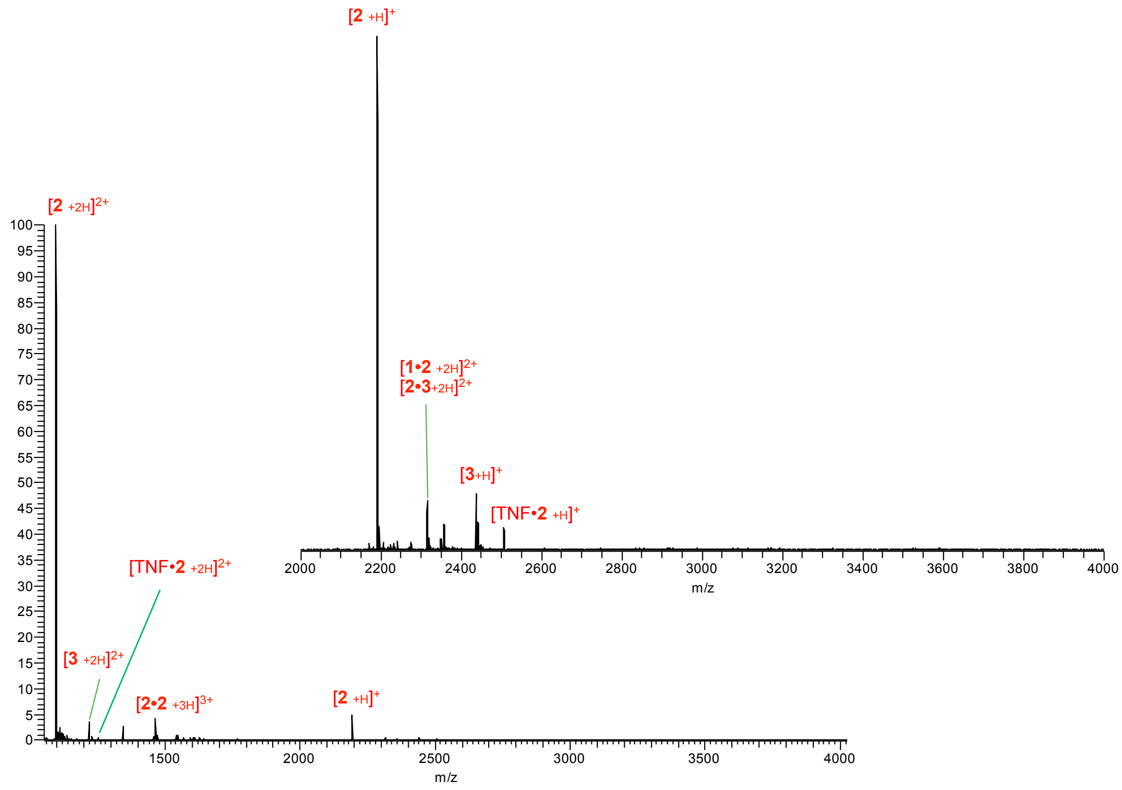

Supplementary Figure 45. End-capping experiment of supramolecular terpolymer *poly*-**1**-**2**-**3** in the presence of 2,4,7-trinitrofluorenone (TNF). ESI-Orbitrap-MS spectra of a mixture of **1**, **2**, **3,** and TNF ([**1**] = [**2**] = [**3**] = 0.07 mmol L^{-1}) in chloroform.

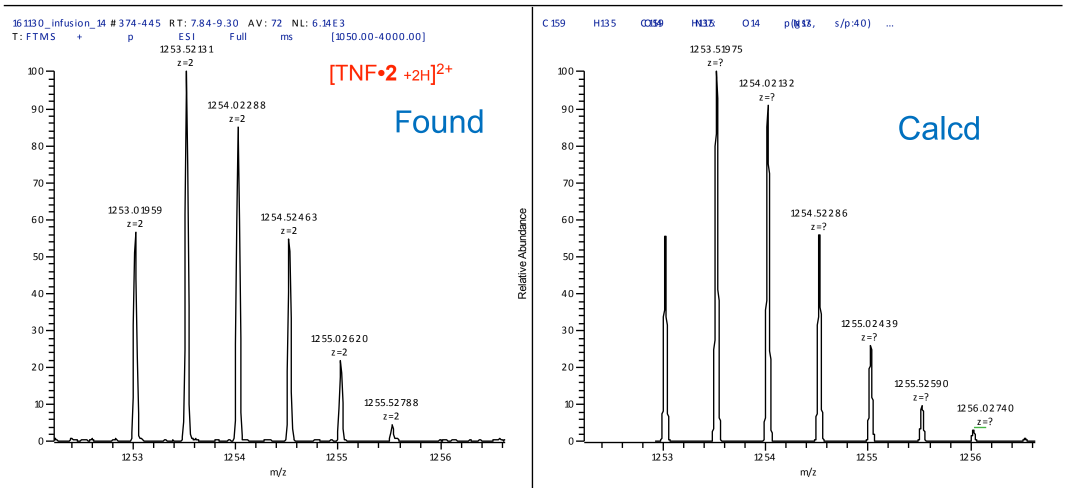

Elemental Composition

m/z	Theo. Mass	Delta (ppm)	RDB equiv.	Composition
1253.01959	1253.01817	1.13	101.0	C159 H135 O14 N17

Supplementary Figure 46. Found and calculated isotope patterns of [TNF•**2** + 2H]$^{2+}$ in a mixture of **1**, **2**, **3,** and TNF ([**1**] = [**2**] = [**3**] = 0.07 mmol L^{-1}) in chloroform.

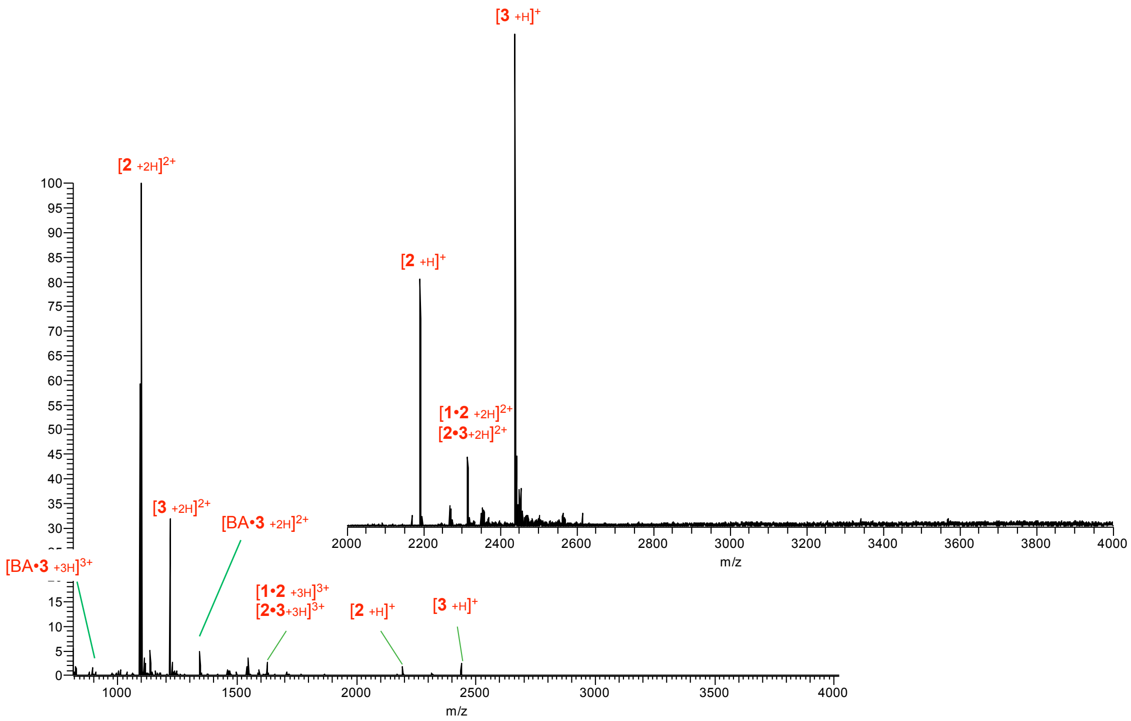

Supplementary Figure 47. End-capping experiment of supramolecular terpolymer *poly*-**1**-**2**-**3** in the presence of 5-(*p*-methoxybenzylidene)barbituric acid (BA). ESI-Orbitrap-MS spectra of a mixture of **1**, **2**, **3**, and BA ([**1**] = [**2**] = [**3**] = 0.07 mmol L^{-1}) in chloroform.

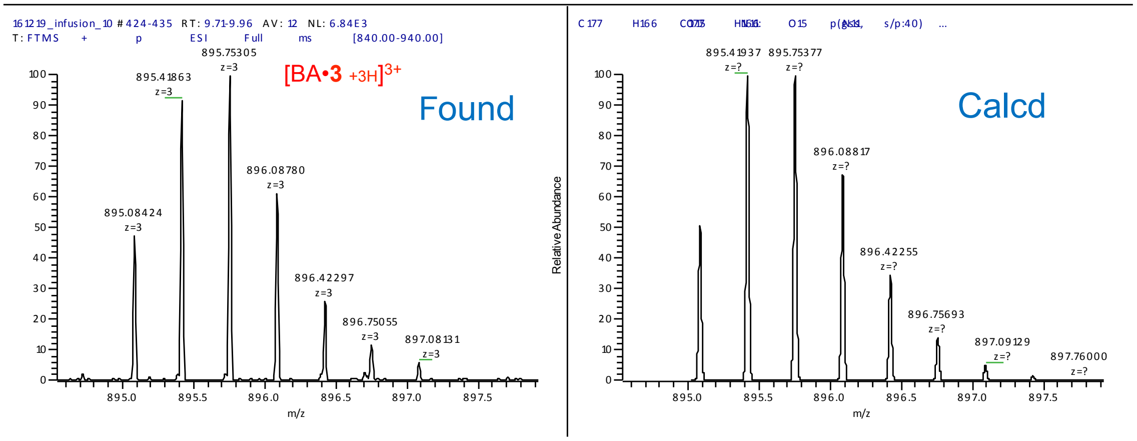

Elemental Composition

m/z	Theo. Mass	Delta (ppm)	RDB equiv.	Composition
895.08424	895.08495	-0.79	100.5	C177 H166 O15 N11

Supplementary Figure 48. Found and calculated isotope patterns of $[BA\cdot\mathbf{3} + 3H]^{3+}$ in a mixture of **1**, **2**, **3,** and BA ([**1**] = [**2**] = [**3**] = 0.07 mmol L^{-1}) in chloroform.

Supplementary Table 7. Concentration dependent diffusion coefficients (*D*) of (a) **1**, (b) **2**, (c) **3** (d) a 1:1 mixture of **1** and **2,** (e) a 1:1 mixture of **2** and **3**, (f) a 1:1 mixture of **3** and **1**, and (g) a 1:1:1 mixture of **1** and **2** and **3**, in chloroform-d_1 at 24 °C. (a), (b) and (c): *C* indicates the concentration of **1**, **2** and **3**. (d), (e), (f) and (g): *C* indicates the averaged concentrations of **1** and **2** {([**1**] + [**2**]) / 2}, **2** and **3** {([**2**] + [**3**]) / 2}, **3** and **1** {([**3**] + [**1**]) / 2}, and **1**, **2** and **3** {([**1**] + [**2**] + [**3**]) / 3}.

(a)

C / mmol L^{-1}	10	7.5	5.0	2.5	1.0
D / 10^{-10} m^2 s^{-1}	3.2 ± 0.1	3.42 ± 0.03	3.28 ± 0.03	3.46 ± 0.03	3.34 ± 0.07

(b)

C / mmol L^{-1}	10	7.5	5.0	2.5	1.0
D / 10^{-10} m^2 s^{-1}	3.4 ± 0.1	3.45 ± 0.08	3.6 ± 0.1	3.3 ± 0.1	3.4 ± 0.2

(c)

C / mmol L^{-1}	10	7.5	5.0	2.5	1.0
D / 10^{-10} m^2 s^{-1}	3.24 ± 0.04	3.05 ± 0.08	3.21 ± 0.03	3.22 ± 0.03	3.12 ± 0.09

(d)

C / mmol L^{-1}	10	7.5	5.0	2.5	1.0
D / 10^{-10} m^2 s^{-1}	2.06 ± 0.01	2.25 ± 0.02	2.68 ± 0.07	3.12 ± 0.03	3.28 ± 0.03

(e)

C / mmol L^{-1}	10	7.5	5.0	2.5	1.0
D / 10^{-10} m^2 s^{-1}	2.2 ± 0.1	2.4 ± 0.1	2.65 ± 0.07	2.73 ± 0.07	3.00 ± 0.05

(f)

C / mmol L^{-1}	10	7.5	5.0	2.5	1.0
D / 10^{-10} m^2 s^{-1}	2.05 ± 0.01	2.23 ± 0.02	2.50 ± 0.02	3.02 ± 0.03	3.09 ± 0.04

(g)

C / mmol L^{-1}	10	7.5	5.0	2.5	1.0	0.10
D / 10^{-10} m^2 s^{-1}	0.56 ± 0.04	1.29 ± 0.01	1.81 ± 0.03	2.46 ± 0.08	3.04 ± 0.04	3.41 ± 0.05

Supplementary Table 8. Concentration dependent viscosity (η) of (a) **1**, (b) **2**, (c) **3** (d) a 1:1 mixture of **1** and **2,** (e) a 1:1 mixture of **2** and **3**, (f) a 1:1 mixture of **3** and **1**, and (g) a 1:1:1 mixture of **1** and **2** and **3**, in chloroform at 24 °C. (a), (b) and (c): *C* indicates the concentration of **1**, **2** and **3**. (d), (e), (f) and (g): *C* indicates the averaged concentrations of **1** and **2** {([**1**] + [**2**]) / 2}, **2** and **3** {([**2**] + [**3**]) /2}, **3** and **1** {([**3**] + [**1**]) /2}, and **1**, **2** and **3** {([**1**] + [**2**] + [**3**]) /3}.

(a)

C / mmol L^{-1}	1.0	2.0	3.0	4.0	5.0
η / mPa s	0.541 ± 0.006	0.548 ± 0.005	0.552 ± 0.006	0.553 ± 0.001	0.558 ± 0.009
C / mmol L^{-1}	6.0	7.0	8.0	9.0	10.0
η / mPa s	0.567 ± 0.04	0.575 ± 0.006	0.580 ± 0.005	0.586 ± 0.006	0.590 ± 0.002

(b)

C / mmol L^{-1}	1.0	2.0	3.0	4.0	5.0
η / mPa s	0.540 ± 0.003	0.543 ± 0.003	0.546 ± 0.003	0.549 ± 0.003	0.555 ± 0.002
C / mmol L^{-1}	6.0	7.0	8.0	9.0	10.0
η / mPa s	0.559 ± 0.004	0.567 ± 0.004	0.576 ± 0.005	0.584 ± 0.005	0.595 ± 0.002

(c)

C / mmol L^{-1}	1.0	2.0	3.0	4.0	5.0
η / mPa s	0.540 ± 0.007	0.549 ± 0.006	0.552 ± 0.01	0.558 ± 0.008	0.562 ± 0.01
C / mmol L^{-1}	6.0	7.0	8.0	9.0	10.0
η / mPa s	0.570 ± 0.01	0.575 ± 0.01	0.582 ± 0.008	0.586 ± 0.007	0.59 ± 0.02

(d)

C / mmol L^{-1}	1.0	2.0	3.0	4.0	5.0
η / mPa s	0.546 ± 0.008	0.550 ± 0.009	0.552 ± 0.009	0.560 ± 0.007	0.567 ± 0.006
C / mmol L^{-1}	6.0	7.0	8.0	9.0	10.0
η / mPa s	0.569 ± 0.008	0.580 ± 0.004	0.59 ± 0.01	0.594 ± 0.01	0.60 ± 0.02

(e)

C / mmol L^{-1}	1.0	2.0	3.0	4.0	5.0
η / mPa s	0.542 ± 0.008	0.548 ± 0.002	0.551 ± 0.002	0.552 ± 0.005	0.56 ± 0.01
C / mmol L^{-1}	6.0	7.0	8.0	9.0	10.0
η / mPa s	0.57 ± 0.01	0.58 ± 0.01	0.596 ± 0.09	0.600 ± 0.009	0.60 ± 0.01

(f)

C / mmol L^{-1}	1.0	2.0	3.0	4.0	5.0
η / mPa s	0.539 ± 0.005	0.54 ± 0.01	0.544 ± 0.001	0.548 ± 0.005	0.55 ± 0.01
C / mmol L^{-1}	6.0	7.0	8.0	9.0	10.0
η / mPa s	0.56± 0.01	0.567 ± 0.005	0.578 ± 0.009	0.581 ± 0.004	0.60 ± 0.01

(g)

C / mmol L^{-1}	1.0	2.0	3.0	4.0	5.0
η / mPa s	0.539 ± 0.008	0.578 ± 0.008	0.612± 0.008	0.654 ± 0.006	0.697 ± 0.007
C / mmol L^{-1}	6.0	7.0	8.0	9.0	10.0
η / mPa s	0.740 ± 0.004	0.77 ± 0.01	0.838 ± 0.005	0.875 ± 0.001	0.95 ± 0.02

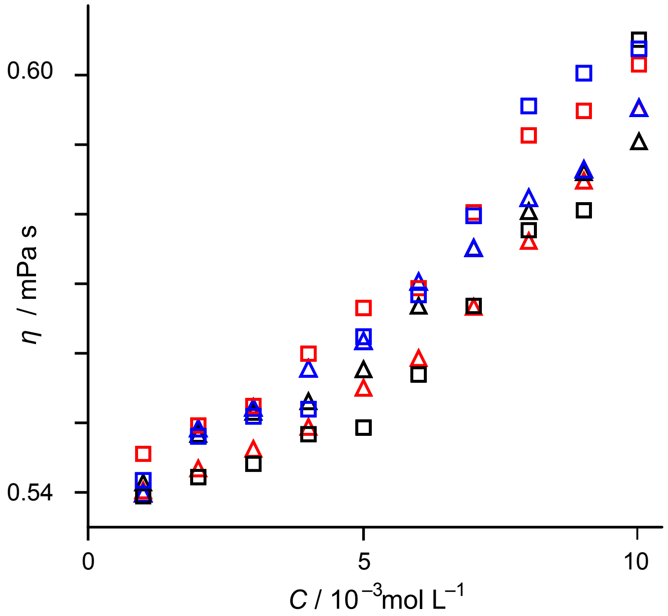

Supplementary Figure 49. Solution viscosities of **1** (black triangle), **2** (red triangle), **3** (blue triangle), a 1:1 mixture of **1** and **2** (red square), a 1:1 mixture of **2** and **3** (blue square), and a 1:1 mixture of **3** and **1** (black square) in chloroform at 24 °C.

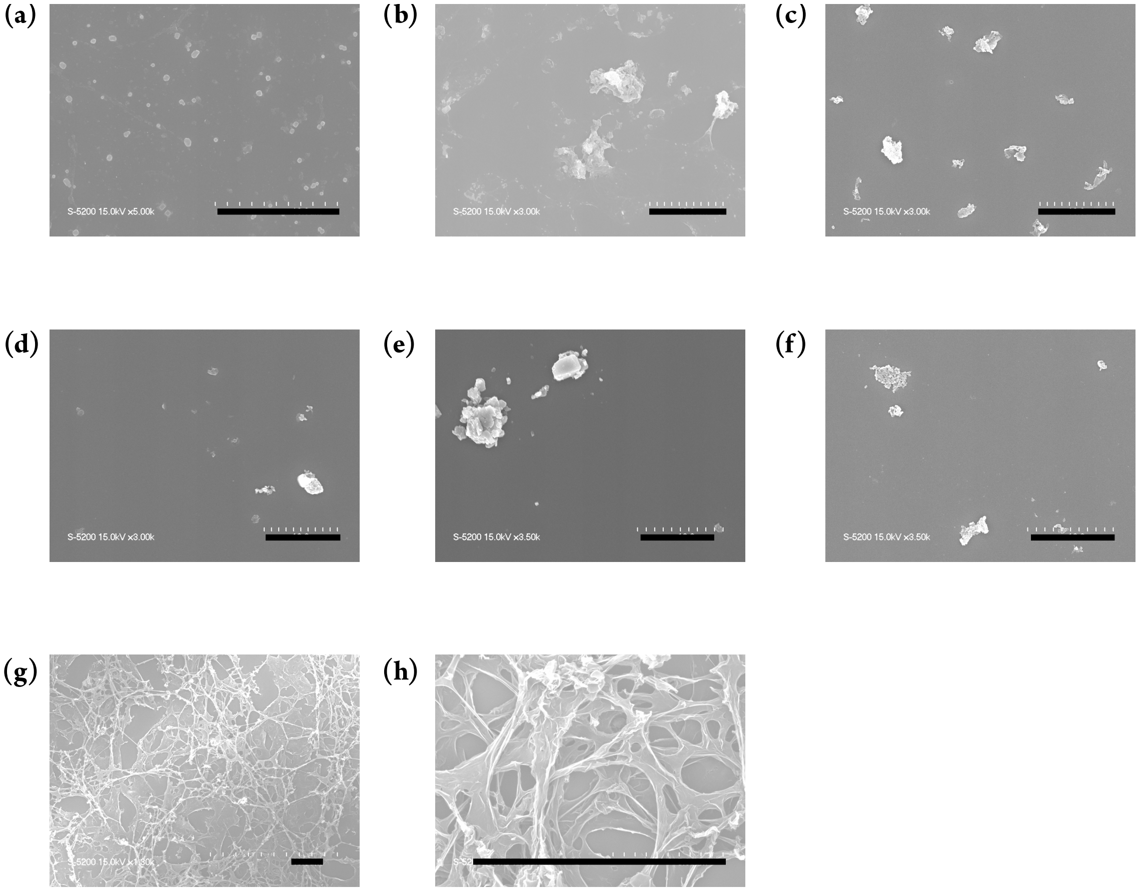

Supplementary Figure 50. The SEM images of the cast films of (a) **1**, (b) **2**, (c) **3**, (d) a 1:1 mixture of **1** and **2**, (e) a 1:1 mixture of **2** and **3**, (f) a 1:1 mixture of **3** and **1**, and (g, h) a 1:1:1 mixture of **1**, **2** and **3**. Scale bars are 10 μm.

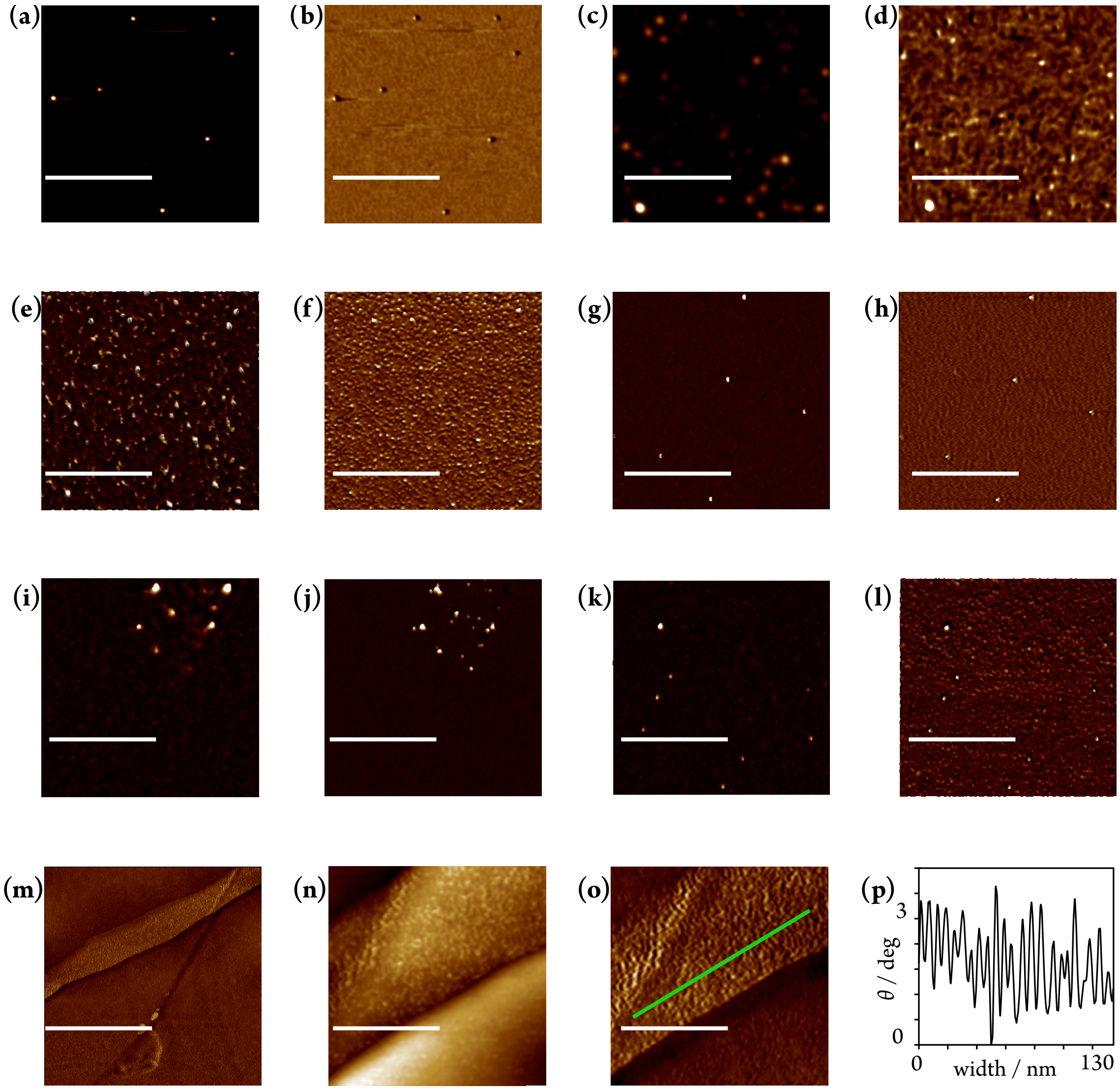

Supplementary Figure 51. The AFM images of the cast films of (a) **1** (topography), (b) **1** (phase), (c) **2** (topography), (d) **2** (phase), (e) **3** (topography), (f) **3** (phase), (g) a 1:1 mixture of **1** and **2** (topography), (h) a 1:1 mixture of **1** and **2** (phase), (i) a 1:1 mixture of **2** and **3** (topography), (j) a 1:1 mixture of **2** and **3** (phase), (k) a 1:1 mixture of **3** and **1** (topography), (l) a 1:1 mixture of **3** and **1** (phase), (m) a 1:1:1 mixture of **1**, **2** and **3** (phase), (n) a 1:1:1 mixture of **1**, **2** and **3** (topography), and (o) a 1:1:1 mixture of **1**, **2** and **3** (phase). (p) The phase profile of the green line in image (o). Scale bars are 500 nm for (a)–(m), and 100 nm for (n) and (o).

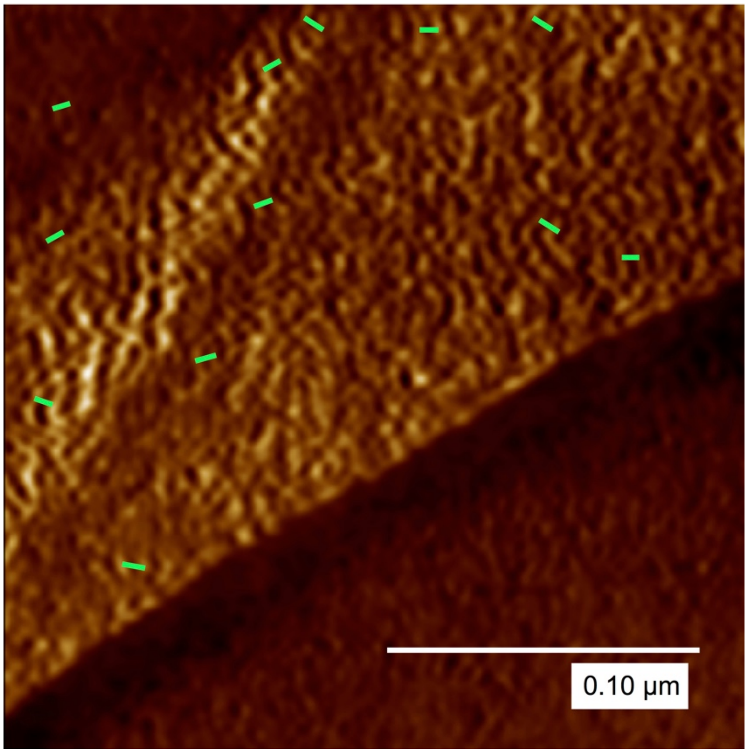

Supplementary Figure 52. AFM image of the cast film of a 1:1:1 mixture of **1**, **2** and **3** (phase). A scale bar is 100 nm.

Supplementary Table 9. The straight-line distance (*d* / nm) between two fibers that arrayed in parallel (green lines in Supplementary Figure 52).

entry	1	2	3	4	5	6	7	8	9	10	11	Averaged
d / nm	3.98	3.92	3.90	3.91	4.08	4.43	4.09	4.19	3.18	4.42	3.06	3.9(4)

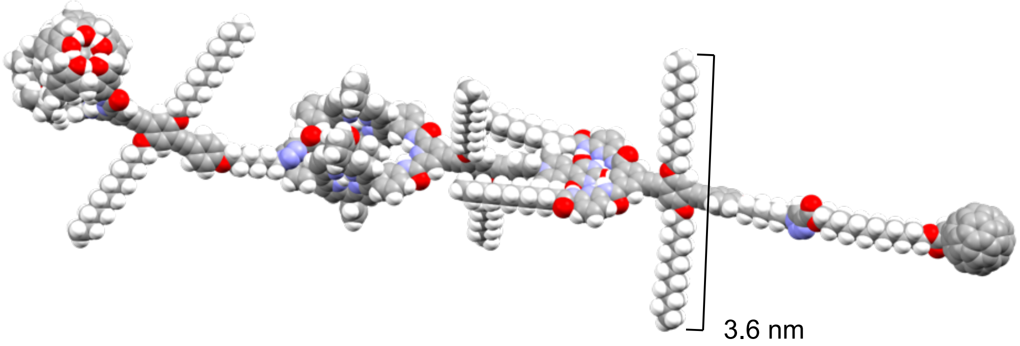

Supplementary Figure 53. Calculated structure of ternary complex **1•2•3** using MacroModel V9.1 with Amber* force field.

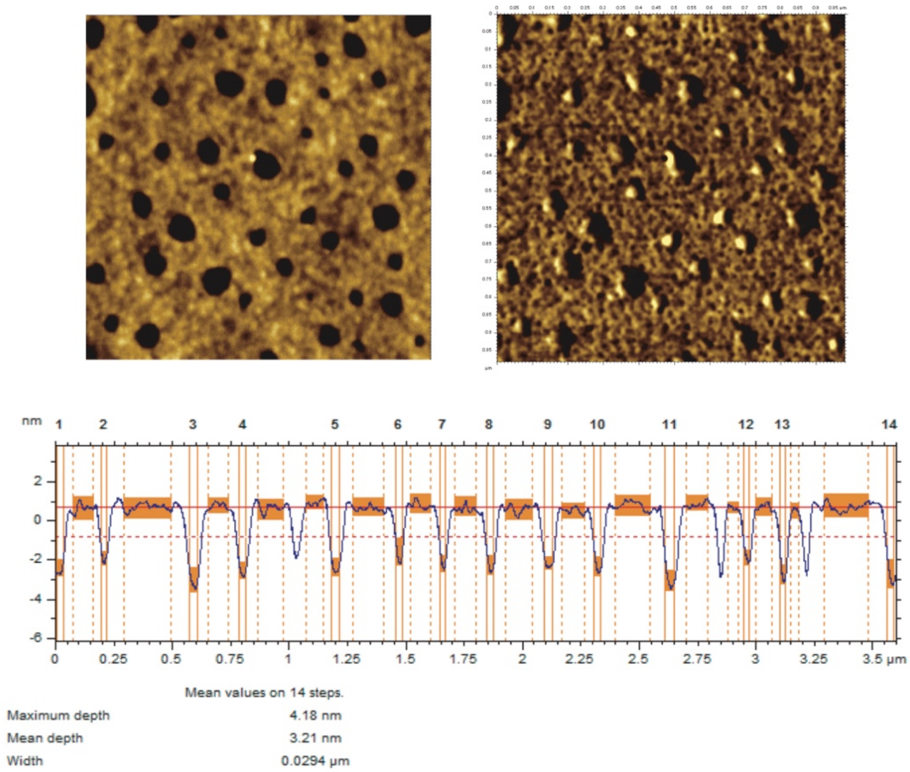

Supplementary Figure 54. AFM images of a thin-film annealed at 80 °C. (Left) Topographic image. (right) Phase images. (bottom) The height profile of the topographic image.

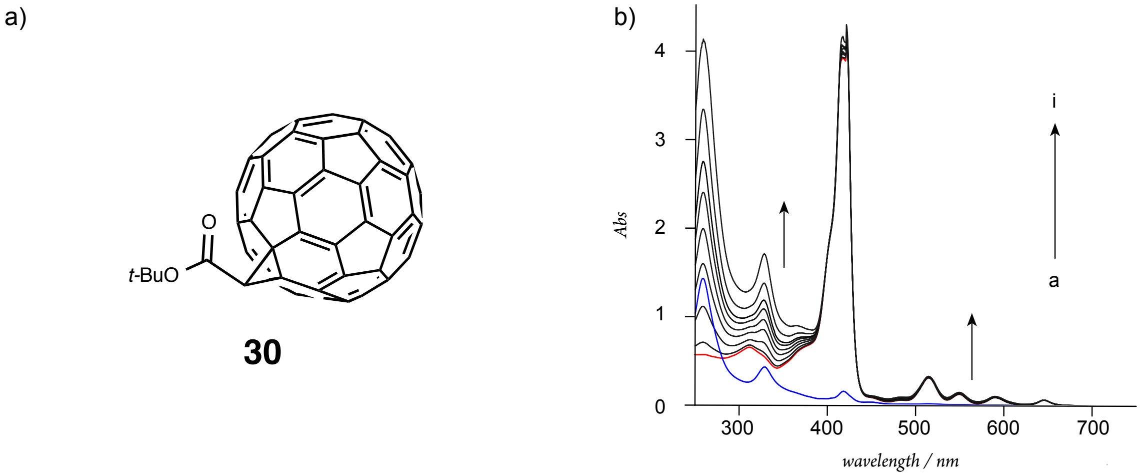

Supplementary Figure 55. (a) Molecular structure of **30**. (b) UV-vis Absorption spectra of **30** (1.0×10^{-5} mol L^{-1}) (blue line) and **2** (1.0×10^{-5} mol L^{-1}) (red line). Concentrations of **30** are (a-i) 0.1, 0.3, 0.6, 0.9, 1.2, 1.5, 1.8, 2.0, 3.0×10^{-5} mol L^{-1} at 25°C in 1,2-dichloroethane (gray line).

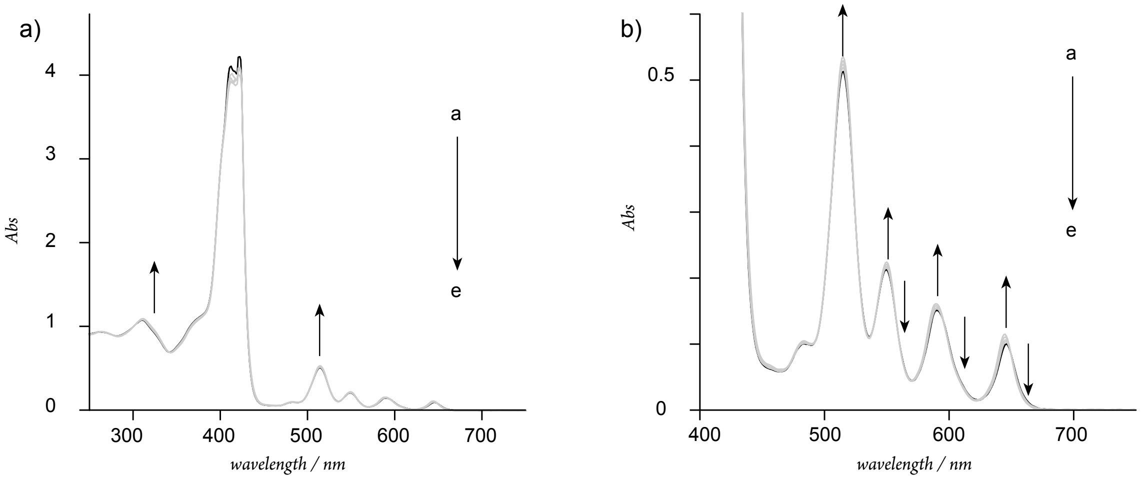

Supplementary Figure 56. (a, b) Variable temperature UV-vis spectra of **2** (1.0×10^{-5} mol L^{-1}) in 1,2-dichloroethane (from a to e: 40, 30, 20, 10, 0 °C).

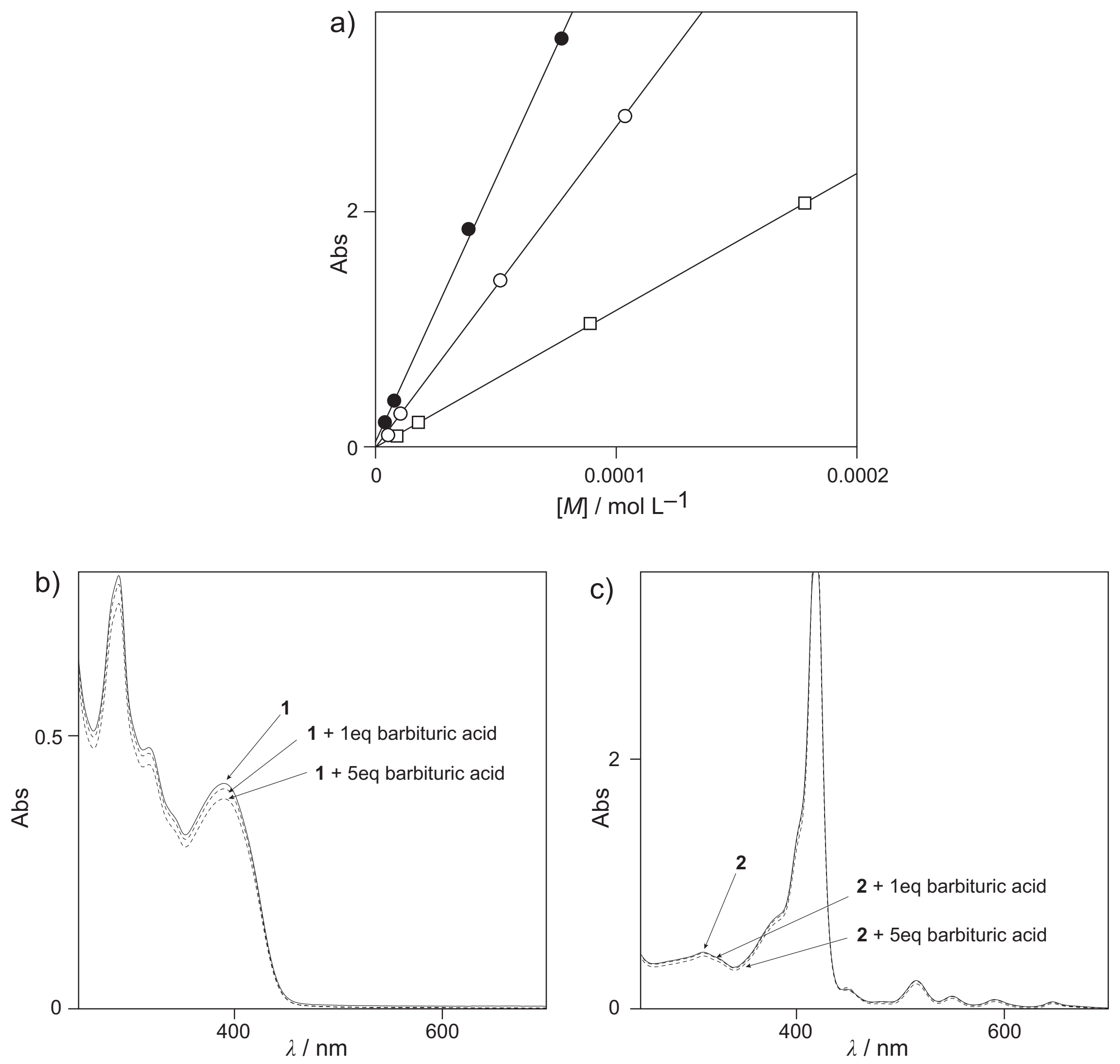

Supplementary Figure 57. a) Calibration plots of monomers **1** (filled circle), **2** (open square), and **3** (open circle), obtained for the absorbances at 400, 520, and 400 nm by UV-vis absorption spectroscopy. The good linear correlations indicate that the monomer aggregation is negligible in the concentration ranges applied for the host-guest titration. UV/vis absorption spectra of **1** (b) and **2** (c) in the presence of barbituric acid.

Supplementary Methods

General: All reagents and solvents were of the commercial reagent grade and were used without further purification except where noted. Dry dimethylformamide (DMF), triethylamine and diisopropylamine were obtained by distillation over CaH_2. ^{1}H and ^{13}C NMR spectra were recorded on a Varian mercury-300 spectrometer, JEOL JNM-ECA500 spectrometer and JEOL JNM-ECA600 spectrometer, and chemical shifts were reported as the delta scale in ppm relative to $CHCl_3$ (δ = 7.26 and 77.0 for ^{1}H and ^{13}C, respectively) and tetrahydrofuran (THF) (δ = 3.58 and 67.2 for ^{1}H and ^{13}C, respectively). UV/vis absorption spectra were recorded on a JASCO V-560 spectrometer. Fluorescence spectra were recorded on a JASCO FP-6500 spectrometer. IR spectra were recorded on JASCO FT/IR-4600 spectrometer. ESI-Mass and APCI-Mass spectra were recorded on Thermo Fisher Scientific LTQ Orbitrap XL hybrid FTMS. FD-Mass spectra were recorded on JEOL JMS-T100 GCV 4G. Preparative separations were performed by silica gel gravity column chromatography (Silica Gel 60N (spherical, neutral)). Recycling preparative GPC-HPLC separations were carried out on JAI LC-908s using preparative JAIGEL-2H, 2H, 1H columns in series. Compound **1**, **2** and **3** were synthesized according to Fig. S1-3. Compound **4**,[1] **6**,[2] **8**,[3] **10**,[4] **12**,[3] **13**,[5] **16**,[6] **17**,[7] **19**,[8], **22**,[9], and **30**[10] were prepared according to the reported methods.

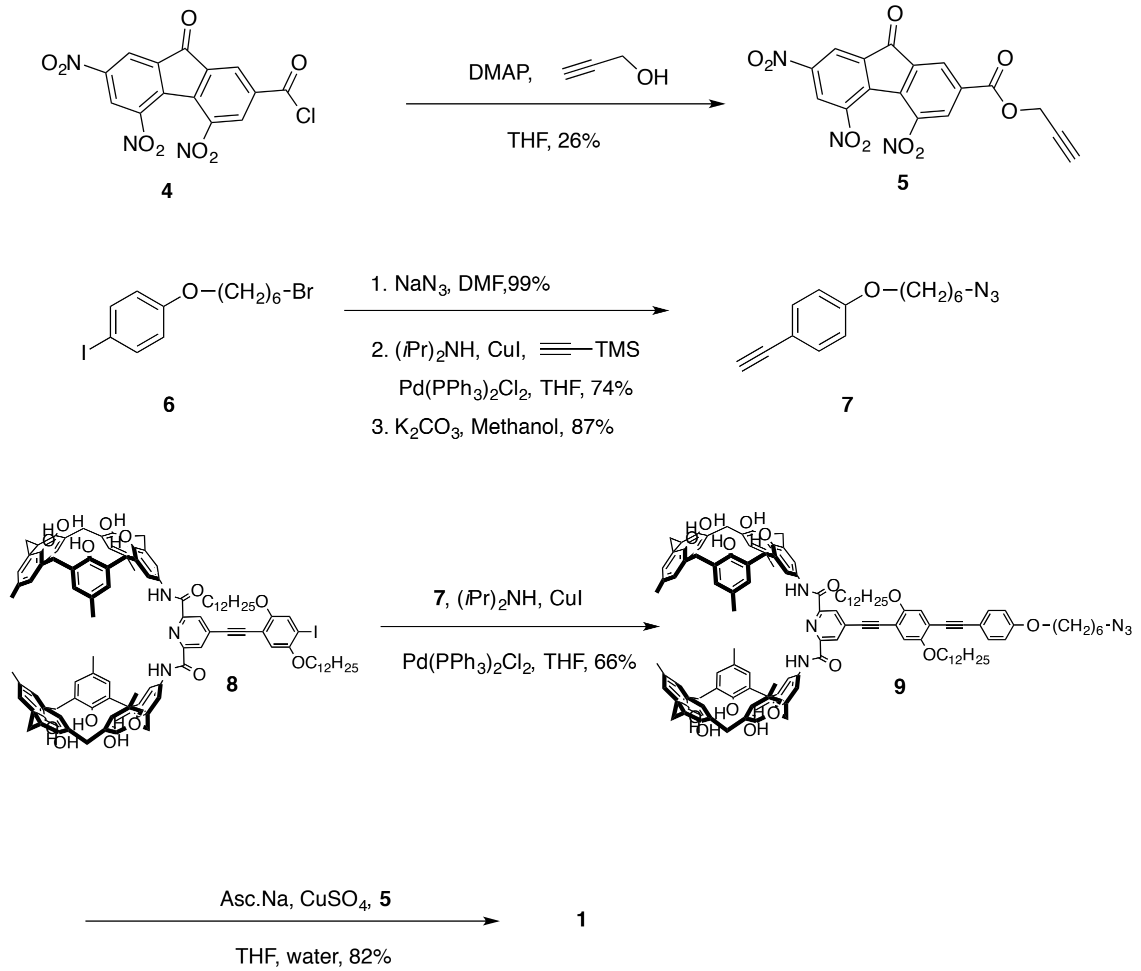

Supplementary Figure 58. Synthesis of compound **1**. DMAP = *N, N*-dimethyl-4-aminopyridine, DMF = dimethylformamide, TMS = trimethylsilyl, THF = tetrahydrofuran, Asc.Na = Sodium ascorbate.

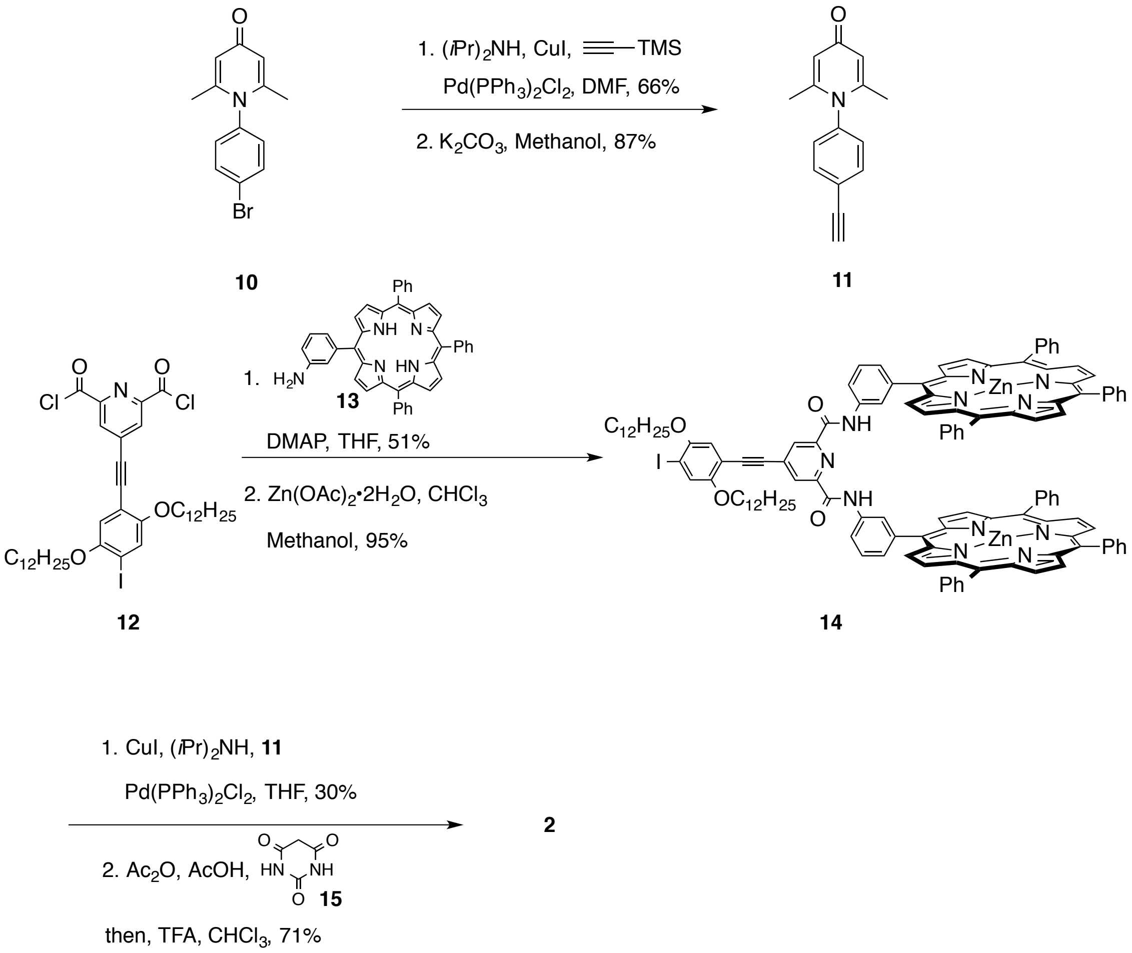

Supplementary Figure 59. Synthesis of compound **2**. Ac = acetyl, TFA = 2,2,2-trifluoroacetic acid.

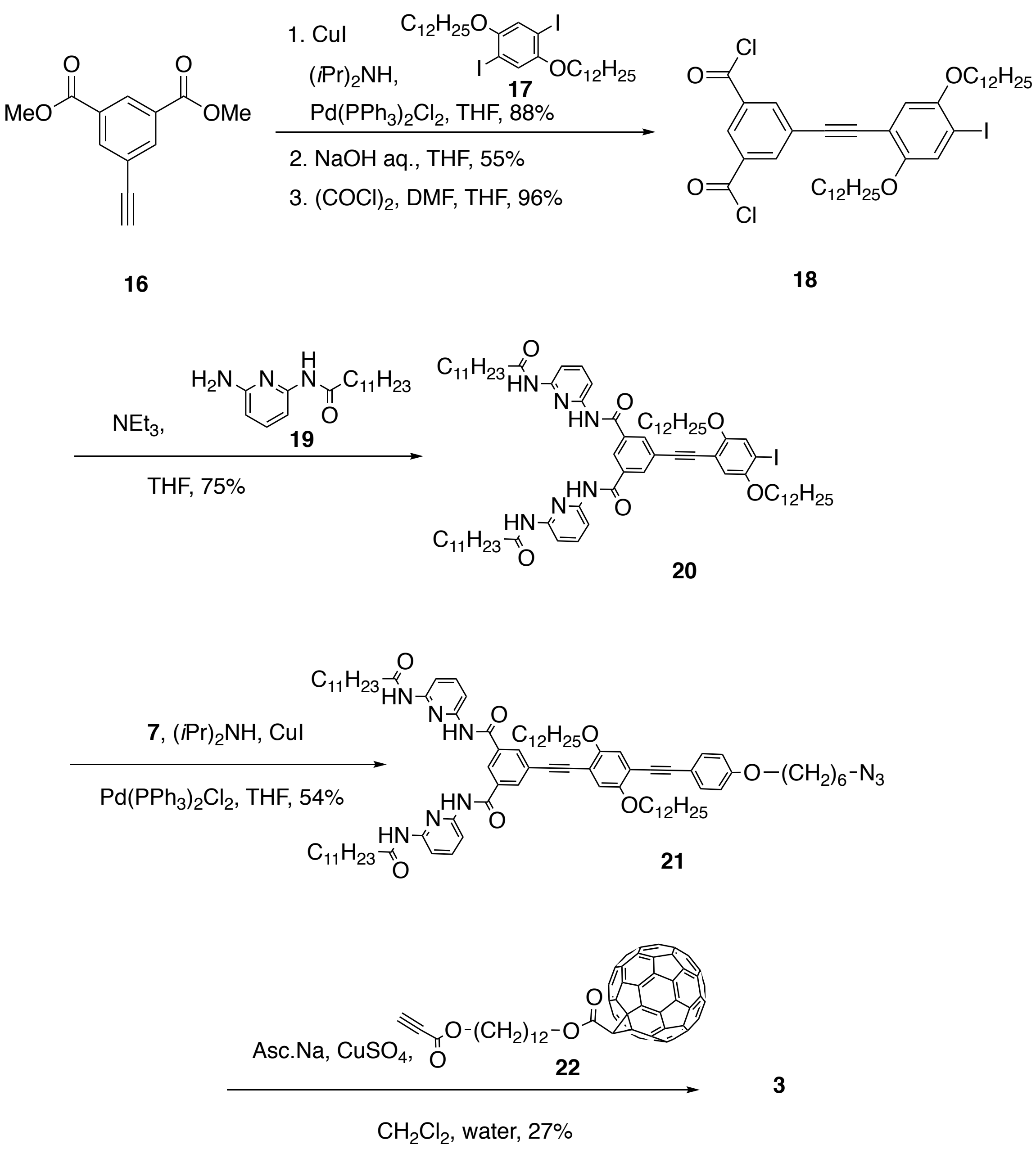

Supplementary Figure 60. Synthesis of compound **3**.

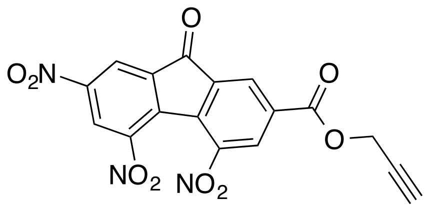

Prop-2-yn-1-yl 4,5,7-trinitro-9-oxo-9H-fluorene-2-carbonylate (**5**): To a solution of **4** (582 mg, 2.23 mmol) in dry THF (50 ml) was added DMAP (1.36 g, 11.3 mmol) and 2-propyn-1-ol (250 mg, 4.46 mmol). After being stirred at room temperature for 6 hours under argon atmosphere, the solution was diluted with ethyl acetate. The organic layer was washed with 1M hydrochloric acid (11.3 ml, 11.3 mmol) and water, dried over Na_2SO_4, and concentrated *in vacuo*. The crude product was purified by column chromatography on silica gel (0%–30% ethyl acetate in *n*-hexane) to give desired product **5** (226mg, 26%) as a yellow solid. M.p.: 56–58 °C; ^{1}H NMR (300 MHz, $CDCl_3$): δ 9.00 (d, 1H, *J* = 2.1 Hz), 8.86 (d, 1H, *J* = 2.1 Hz), 8.85 (d, 1H, *J* = 1.6 Hz), 8.76 (d, 1H, *J* = 1.6 Hz), 5.05 (d, 2H, *J* = 2.4 Hz), 2.62 (t, 1H, *J* = 2.4 Hz); ^{13}C NMR (75 MHz, $CDCl_3$) δ184.8, 161.9, 149.6, 146.8, 146.5, 138.4, 138.3, 137.7, 136.5, 134.7, 131.8, 129.3, 125.4, 122.7, 76.4, 76.3, 54.0; IR (ATR): *ν* 3268, 3078, 2925, 2910, 2834, 2108, 1729, 1619, 1597, 1529, 1434, 1429, 1378, 1352, 1340, 1286, 1271, 1230, 1154, 1115, 1073, 1038, 982, 910, 869 cm^{-1}; HRMS (ESI$^-$): calcd. for $C_{17}H_7N_3O_9$ *m*/*z* 397.0188 M$^-$, found *m*/*z* 397.0183.

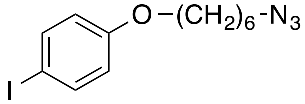

1-[(6-Azidohexyl)oxy]-4-iodobenzene (**23**): To a solution of **6** (3.48 g, 9.10 mmol) in dry DMF (18 ml) was added NaN_3 (1.18 g, 18.2 mmol). After being stirred at 100 °C for 10 hours under argon atmosphere, the reaction mixture was added ethyl acetate (30 ml). After being filtered, the filtrate was washed with brine, dried over Na_2SO_4 and concentrated *in vacuo* to give desired product **23** (3.10 g, 99%) as clear oil. ^{1}H NMR (300 MHz, $CDCl_3$): δ 7.54 (d, 2H, *J* = 8.6 Hz), 6.67 (d, 2H, *J* = 8.6 Hz), 3.92 (t, 2H, *J* = 6.4 Hz), 3.28 (t, 2H, *J* = 6.8 Hz), 1.72–1.85 (m, 2H), 1.56–1.69 (m, 2H), 1.36–1.55 (m, 4H); ^{13}C NMR (75 MHz, $CDCl_3$): δ 158.8, 138.1, 116.8, 82.5, 67.8, 51.3, 29.0, 28.7, 26.3, 25.6; IR (ATR): *ν* 2929, 2854, 2085, 1584, 1569, 1484, 1466, 1386, 1318, 1249, 1238, 1172, 1112, 1063, 1058, 1003, 995, 816, 727 cm^{-1}; HRMS (FD$^+$): calcd. for $C_{12}H_{16}N_3OI$ *m*/*z* 345.0338 M$^+$, found 345.0332.

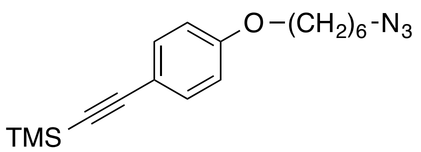

[{4-((6-Azidohexyl)oxy)phenyl}ethynyl]trimethylsilane (**24**): To a solution of **23** (3.10 g, 8.98 mmol) in dry THF (80 ml) was added CuI (171 mg, 0.898 mmol) and dry diisopropylamine (3.1 ml, 23 mmol). After being stirred at room temperature for 15 minutes under argon atmosphere, trimethylsilylacetylene (1.5 ml, 11 mmol) was added. $Pd(PPh_3)_2Cl_2$ (315 mg, 0.449 mmol) was added to the resultant solution. After being stirred at room temperature for 15 hours under argon atmosphere in the dark, the reaction mixture was passed through florisil column, and diluted with ethyl acetate. The organic layer was washed with saturated aqueous NH_4Cl and brine, dried over Na_2SO_4, and concentrated *in vacuo*. The crude product was purified by column chromatography on silica gel (0%–20% ethyl acetate in *n*-hexane) to give desired product **24** (2.10 g, 74%) as clear oil. ^{1}H NMR (300 MHz, $CDCl_3$): δ 7.39 (d, 2H, J = 8.5 Hz), 6.80 (d, 2H, J = 8.5 Hz), 3.95 (t, 2H, J = 6.5 Hz), 3.28 (t, 2H, J = 6.6 Hz), 1.73–1.84 (m, 2H), 1.58–1.69 (m, 2H), 1.38–1.54 (m, 4H), 0.23 (s, 9H); ^{13}C NMR (75 MHz, $CDCl_3$) δ159.2, 133.4, 115.0, 114.2, 105.2, 92.3, 67.7, 51.3, 29.0, 28.7, 26.5, 25.6, 0.0 ppm. IR (ATR): ν 3002, 2930, 2853, 2151, 2087, 1602, 1541, 1505, 1467, 1385, 1315, 1258, 1243, 1106, 1112, 1009, 990, 845, 829, 755 cm^{-1}; HRMS (FD$^+$): calcd. for $C_{17}H_{25}N_3OSi$ *m*/*z* 315.1767 M$^+$, found *m*/*z* 315.1757.

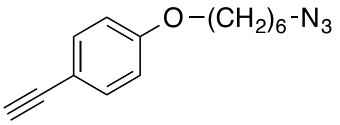

1-[(6-Azidohexyl)oxy]-4-ethynylbenzene (**7**): To a solution of **24** (126 mg, 0.4 mmol) in methanol (11 ml) was added potassium carbonate (221 mg, 1.6 mmol). After being stirred at room temperature for 20 minutes under argon atmosphere, the reaction mixture was extracted with ethyl acetate. The organic layer was washed with brine, dried over Na_2SO_4 and concentrated *in vacuo* to give desired compound **7** (92.5 mg, 95%) as yellow oil. ^{1}H NMR (300 MHz, $CDCl_3$): δ 7.42 (d, 2H, J = 8.6 Hz), 6.83 (d, 2H, J = 8.6 Hz), 3.96 (t, 2H, J = 6.3 Hz), 3.28 (t, 2H, J = 6.3 Hz), 2.99 (s, 1H), 1.73–1.88 (m, 2H), 1.57–1.70 (m, 2H), 1.39–1.54 (m, 4H); ^{13}C NMR (75 MHz, $CDCl_3$) δ159.4, 133.5, 114.4, 113.9, 83. 7, 75.7, 67.7, 51.3, 29.0, 28.8, 26.5, 25.6 ppm. IR (ATR): ν 3285, 2939, 2862, 2187, 2104, 1604, 1566, 1505, 1458, 1393, 1363, 1308, 1255, 1231, 1171, 1111, 1022, 1003, 994, 916, 831, 729 cm^{-1}; HRMS (FD$^+$): calcd. for $C_{14}H_{17}N_3O$ *m*/*z* 243.1372 M$^+$, found *m*/*z* 243.1372.

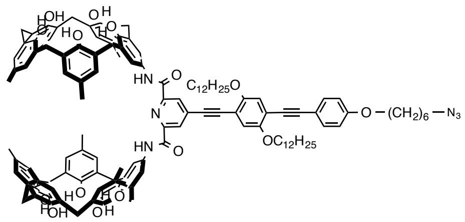

Biscalix[5]arene **9**: To a solution of **8** (170 mg, 0.130 mmol) in dry THF (3.5 ml) was added CuI (2.9 mg, 0.015 mmol) and dry diisopropylamine (0.10 ml, 0.71 mmol). After being stirred at room temperature for 15 minutes under argon atmosphere, **7** (63.4 mg, 0.261 mmol) was added. $Pd(PPh_3)_2Cl_2$ (10.5 mg, 0.015 mmol) was added to the solution. After being stirred at room temperature for 22 hours under argon atmosphere in the dark, the reaction mixture was passed through florisil column, and diluted with ethyl acetate. The organic layer was washed with saturated aqueous NH_4Cl and brine, dried over Na_2SO_4 and concentrated *in vacuo*. The crude product was purified by column chromatography on silica gel (0%–30% ethyl aceate in *n*-hexane). Reprecipitation (*n*-hexane-chloroform) gave desired product **9** (122 mg, 66%) as a yellow solid. M.p.: >190 °C (dec.); ^1H NMR (300 MHz, $CDCl_3$): δ 9.28 (s, 2H), 9.09 (s, 2H), 8.80 (s, 4H), 8.78 (s, 4H), 8.50 (s, 2H), 7.61 (s, 4H), 7.48 (d, 2H, *J* = 8.5 Hz), 6.91–7.07 (m, 18H), 6.88 (d, 2H, *J* = 8.5 Hz), 3.93–4.12 (m, 6H), 3.58–4.00 (m, 20H), 3.30 (t, 2H, *J* = 6.4 Hz), 2.23 (s, 12H), 2.14 (s, 12H), 2.03–2.29 (m, 4H), 1.74–1.95 (m, 4H), 1.14–1.67 (m, 40H), 0.87 (t, 3H, *J* = 6.8 Hz), 0.80 (t, 3H, *J* = 6.8 Hz); ^{13}C NMR (75 MHz, $CDCl_3$): δ161.1, 159.3, 154.1, 153.2, 149.5, 147.8, 147.7, 135.6, 134.0, 133.1, 132.8, 130.8, 130.6, 130.4, 129.8, 129.6, 127.5, 126.9, 126.5, 126.4, 125.7, 121.9, 117.2, 116.5, 116.4, 115.1, 114.6, 114.4, 111.2, 96.0, 94.3, 90.8, 84.4, 69.6, 67.7, 51.3, 31.9, 31.9, 31.5, 31.3, 29.7, 29.6, 29.5, 29.4, 29.3, 29.1, 29.0, 28.8, 26.5, 26.1, 25.9, 25.7, 22.7, 22.6, 20.4, 20.4, 14.1, 14.1; IR (ATR): 3266, 2917, 2850, 2187, 2085, 1670, 1595, 1516, 1479, 1373, 1281, 1242, 1167, 996, 988, 869, 835, 788 cm^{-1}; HRMS (ESI$^-$): calcd. for $C_{131}H_{144}O_{15}N_6$ *m/z* 1020.5350 $[M–H]^{2–}$, found *m/z* 1020.5346.

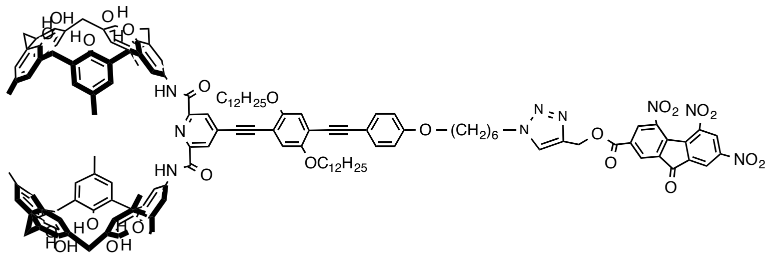

Trinitrofluorenobiscalix[5]arene **1**: To a solution of **9** (177 mg, 92.5 μmol) in dry THF (6.6 ml) and distilled water (0.7 ml) was added **5** (73.4 mg, 185 μmol), $CuSO_4$ (29.5 mg, 185 μmol) and Asc.Na (36.7 mg, 185 μmol). After being stirred at 25 °C for 13 hours under argon atmosphere, the reaction mixture was extracted with ethyl acetate. The organic layer was washed with saturated aqueous NH_4Cl and brine, dried over Na_2SO_4, and concentrated *in vacuo*. The crude product was purified by column chromatography on silica gel (0%–50% ethyl acetate in *n*-hexane). The mixture was further purified by GPC to give desired product **1** (176 mg, 82%) as a yellow solid. M.p.: >150 °C (dec.); ^{1}H NMR (600 MHz, $CDCl_3$): δ 9.28 (s, 2H), 9.10 (s, 2H), 8.81 (m, 5H), 8.79 (s, 4H), 8.67 (s, 2H), 8.64 (s, 1H), 8.48 (s, 2H), 7.66 (s, 1H), 7.60 (s, 4H), 7.36 (d, 2H, *J* = 8.5 Hz), 6.91–7.01 (m, 18H), 6.76 (d, 2H, *J* = 8.5 Hz), 5.53 (s, 2H), 4.38 (t, 2H, *J* = 6.7 Hz), 4.02 (t, 2H, *J* = 6.7 Hz), 4.00 (t, 2H, *J* = 6.7 Hz), 3.89 (t, 2H, *J* =6.7 Hz), 3.31–4.27 (m, 20H), 2.23 (s, 12H), 2.14 (s, 12H), 1.94 (m, 2H), 1.86 (m, 4H), 1.73 (m, 2H), 1.14–1.64 (m, 40H), 0.87 (t, 3H, *J* = 6.8 Hz), 0.81 (t, 3H, *J* = 6.8 Hz); ^{13}C NMR (150 MHz, $CDCl_3$) δ 184.6, 162.5, 161.0, 159.1, 154.1, 153.2, 149.5, 149.3, 147.8, 147.8, 147.8, 146.5, 146.2, 141.4, 138.2, 138.0, 137.5, 136.1, 135.5, 134.8, 133.1, 131.3, 130.8, 130.6, 130.5, 129.8, 129.7, 129.1, 127.5, 126.9, 126.6, 126.5, 126.4, 125.8, 125.1, 124.2, 122.5, 121.9, 117.1, 116. 5, 116.2, 115.1, 114.4, 111.3, 95.9, 94.1, 91.0, 84.6, 69.6, 67.5, 59.5, 50.3, 31.9, 31.9, 31.6, 31.4, 29.9, 29.7, 29.7, 29.6, 29.5, 29.4, 29.3, 29.1, 28.7, 26.1, 26.0, 25.2, 22.7, 22.6, 20.4, 20.4, 14.1, 14.1; IR (ATR): 3224, 2920, 2849, 2194, 1733, 1684, 1593, 1538, 1480, 1340, 1251, 1223, 1168, 992, 909, 840, 827, 773 cm^{-1}; HRMS (ESI$^-$): calcd. for $C_{148}H_{152}O_{24}N_9$ *m/z* 2439.0956 [M–H]$^-$, found *m/z* 2439.0922.

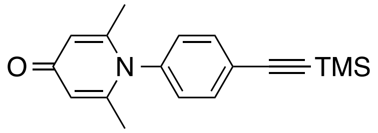

1-[4-(2-Trimethylsilyl)ethynylphenyl]-2,6-dimethyl-4-pyridinone (**25**): To a solution of **10** (1.55 g, 5.57 mmol) in dry DMF (7 ml) was added CuI (106 mg, 0.557 mmol) and dry triethylamine (4.0 ml, 29 mmol). After being stirred at room temperature for 15 minutes under argon atmosphere, $Pd(PPh_3)_2Cl_2$ (193 mg, 0.279 mmol) was added. After being stirred at room temperature for 15 minutes under argon atmosphere, trimethylsilylacetylene (4.5 ml, 32 mmol) was added to the resultant solution. After being stirred at 90 °C for 12 hours under argon atmosphere in the dark, the reaction mixture was passed through florisil column, and diluted with ethyl acetate. The organic layer was washed with saturated aqueous NH_4Cl and brine, dried over Na_2SO_4 and concentrated *in vacuo*. The crude product was purified by column chromatography on silica gel (0%–50% ethyl acetate in *n*-hexane, then 10% methanol in chloroform) to give desired product **25** (1.09 g, 66%) as a yellow solid. M.p.: >171 °C (dec.); ^{1}H NMR (300 MHz, $CDCl_3$): δ 7.63 (d, 2H, J = 8.3 Hz), 7.15 (d, 2H, J = 8.3 Hz), 6.28 (s, 2H), 1.88 (s, 6H), 0.27 (s, 9H); ^{13}C NMR (75 MHz, $CDCl_3$) δ179.4, 148.4, 139.1, 133.6, 127.9, 125.0, 117.5, 102.9, 97.4, 21.4, –0.23; IR (ATR): ν 3031, 2967, 2951, 2157, 1633, 1597, 1573, 1552, 1502, 1464, 1405, 1355, 1255, 1249, 1223, 1181, 1113, 1020, 993, 991, 940, 843, 835 cm^{-1}; HRMS (ESI$^+$): calcd. for $C_{18}H_{22}NOSi$ *m*/*z* 296.1465 [M+H]$^+$, found *m*/*z* 296.1467.

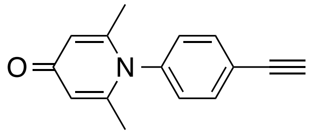

1-(4-Ethynylphenyl)-2,6-dimethyl-4-pyridinone (**11**): To a solution of **25** (120 mg, 0.406 mmol) in methanol (12 ml) was added potassium carbonate (561 mg, 4.06 mmol). After being stirred at room temperature for 10 minutes under argon atmosphere, the reaction mixture was extracted with ethyl acetate. The organic layer was washed with brine, dried over Na_2SO_4 and concentrated *in vacuo* to give desired compound **11** (78.5 mg, 87%) as a yellow solid. M.p.: >200 °C (dec.); ^{1}H NMR (300 MHz, $CDCl_3$): δ 7.67 (d, 2H, J = 8.3 Hz), 7.19 (d, 2H, J = 8.3 Hz), 6.30 (s, 2H), 3.22 (s, 1H), 1.90 (s, 6H); ^{13}C NMR (75 MHz, $CDCl_3$) δ179.4, 148.5, 139.5, 133.8, 128.1, 124.0, 117.5, 81.7, 79.8, 21.4; IR (ATR): ν 3147, 3092, 3036, 2963, 2913, 2087, 1634, 1600, 1557, 1506, 1464, 1418, 1355, 1265, 1193, 1096, 1038, 1014, 986, 943, 852, 829 cm^{-1}; HRMS (ESI$^+$): calcd for $C_{15}H_{14}NO$ *m*/*z* 224.1070 [M+H]$^+$, found *m*/*z* 224.1067.

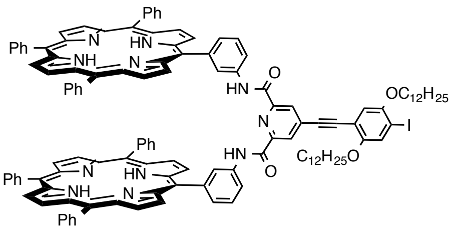

Bisporphyrin mono iodide **26**: To a solution of **12** (43.1 mg, 54.0 μmol) in dry THF (6 ml) was added **13** (338 mg, 243 μmol) and DMAP (79.2 mg, 648 μmol). After being stirred for 9 hours at room temperature under argon atmosphere, the reaction mixture was extracted with chloroform. The organic layer was washed with brine, dried over Na_2SO_4 and concentrated *in vacuo*. The crude product was purified by column chromatography on silica gel (30% ethyl acetate in *n*-hexane). Reprecipitation (*n*-hexane-chloroform) gave desired product **26** (54.7 mg, 51%) as a purple solid. M.p.: >300 °C; ^{1}H NMR (300 MHz, $CDCl_3$): δ 9.53 (s, 2H), 8.84 (d, 4H, *J* = 4.8 Hz), 8.80–8.85 (m, 8H), 8.77 (d, 4H, *J* = 4.8 Hz), 8.63 (s, 2H), 8.05–8.30 (m, 18H), 7.97 (d, 2H, *J* = 8.1 Hz), 7.93 (d, 2H, *J* = 8.3 Hz), 7.65–7.80 (m, 14H), 7.56 (m, 2H), 7.29 (s, 1H), 6.81 (s, 1H), 3.93 (t, 2H, *J* = 6.7 Hz), 3.92 (t, 2H, *J* = 6.7 Hz), 1.7–1.85 (m, 4H), 1.05–1.55 (m, 36H), 0.85 (t, 3H, *J* = 6.9 Hz), 0.76 (t, 3H, *J* = 6.9 Hz), –2.84 (s, 4H); ^{13}C NMR (75 MHz, $CDCl_3$): δ 160.4, 154.8, 151.9, 150.1, 147.7, 143.3, 142.4, 142.4, 135.7, 135.0, 134.9, 134.7, 134.1, 131.6, 127.9, 127.8, 127.3, 126.9, 126.8, 126.5, 125.8, 124.0, 123.9, 120.6, 120.4, 119.7, 119.2, 116.2, 111.6, 93.1, 90.1, 89.8, 70.4, 69.9, 32.2, 32.1, 30.0, 30.0, 29.9, 29.9, 29.8, 29.8, 29.8, 29.7, 29.6, 29.5, 29.4, 29.4, 29.2, 26.4, 26.0, 23.0, 22.9, 14.4, 14.4; IR (ATR): *v* 3294, 3014, 2913, 2845, 2193, 1800, 1683, 1582, 1520, 1464, 1429, 1338, 1202, 1175, 1150, 1068, 1004, 997, 960, 928, 898 cm^{-1}; HRMS (ESI$^+$): calcd. for $C_{127}H_{115}N_{11}O_4I$ *m/z* 1984.8178 $[M+H]^+$, found *m/z* 1984.8208.

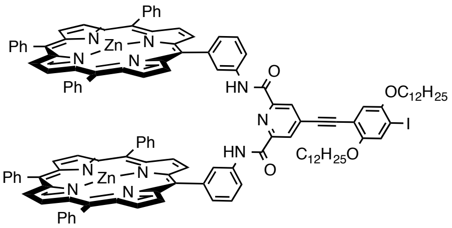

Zinc bisporphyrin monoidodide **14**: To a solution of **26** (740 mg, 0.373 mmol) in chloroform (43 ml) and methanol (21 ml) was added Zinc acetate dihydrate (696 mg, 3.17 mmol). After being stirred for 1 hour at room temperature under argon atmosphere, the reaction mixture was extracted with ethyl acetate. The organic layer was washed with brine, dried over Na_2SO_4, and concentrated *in vacuo* to give desired product **14** (750 mg, 95%) as a purple solid. M.p.: >300 °C; ^{1}H NMR (300 MHz, $CDCl_3$): δ 9.42 (s, 2H), 8.90 (d, 4H, J = 4.6 Hz), 8.89 (d, 4H, J = 4.6 Hz), 8.82 (d, 4H, J = 4.3 Hz), 8.77 (d, 4H, J = 4.3 Hz), 8.48 (s, 2H), 8.05–8.30 (m, 18H), 7.83 (d, 2H, J = 7.3 Hz), 7.30–7.80 (m, 17H), 6.95 (m, 1H), 6.35 (s, 1H), 3.77 (m, 2H), 3.48 (m, 2H), 1.7–1.85 (m, 4H), 1.00–1.55 (m, 36H), 0.85 (t, 3H, J = 6.8 Hz), 0.75 (t, 3H, J = 6.8 Hz), –2.84 (s, 4H); ^{13}C NMR (75 MHz, $CDCl_3$): δ 160.3, 153.5, 151.2, 150.1, 150.1, 149.9, 149.6, 147.0, 143.4, 142.8, 142.7, 135.0, 134.6, 134.5, 134.3, 132.8, 132.0, 131.5, 127.3, 127.2, 126.9, 126.4, 126.4, 126.0, 124.8, 123.4, 121.2, 120.9, 119.6, 119.2, 114.9, 110.5, 92.2, 89.6, 88.9, 69.8, 69.3, 31.9, 31.8, 29.7, 29.7, 29.6, 29.6, 29.5, 29.4, 29.4, 29.4, 29.3, 29.1, 29.0, 28.5, 26.1, 25.4, 22.7, 22.6, 14.2, 14.1; IR (ATR): ν 3288, 3027, 2911, 2844, 2187, 1796, 1669, 1593, 1516, 1476, 1433, 1336, 1254, 1200, 1173, 1074, 1063, 990, 940, 896 cm^{-1}; HRMS (ESI$^+$): calcd. for $C_{127}H_{110}IN_{11}O_4Zn$ m/z 2107.6364 M$^+$, found m/z 2107.6366.

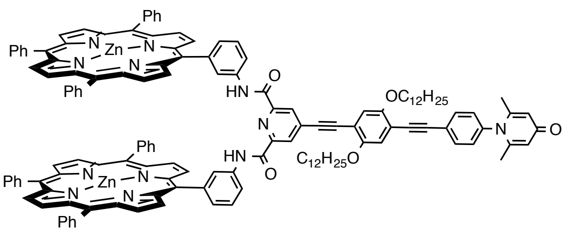

Zinc bisporphyrin **27**: To a solution of **14** (438 mg, 0.207 mmol) in dry THF (5 ml) was added CuI (3.9 mg, 0.021 mmol) and dry triethylamine (1.0 ml, 7.1mmol). After being stirred at room temperature for 15 minutes under argon atmosphere, **11** (78.5 mg, 0.352 mmol) was added. After being stirred at room temperature for 15 minutes under argon atmosphere, $Pd(PPh_3)_2Cl_2$ (7.3mg, 0.010 mmol) was added to the resultant solution. After being stirred at room temperature for 12 hours under argon atmosphere in the dark, the reaction mixture was passed through florisil column, and diluted with dichloromethane. The organic layer was washed with saturated aqueous NH_4Cl and brine, dried over Na_2SO_4, and concentrated *in vacuo*. The crude product was purified by column chromatography on silica gel (0%–70% ethyl acetate in *n*-hexane, then methanol in chloroform 20%) to give desired product **27** (135 mg, 30%) as a purple solid. M.p.: >300 °C; ^{1}H NMR (600 MHz, THF-d_8): δ 11.45 (s, 2H), 8.86 (d, 4H, J = 5.0 Hz), 8.79 (d, 4H, J = 5.0 Hz), 8.72 (d, 4H, J = 5.0 Hz), 8.64 (s, 2H), 8.59 (d, 4H, J = 5.0 Hz), 8.55 (d, 2H, J = 7.4 Hz), 8.46 (s, 2H), 8.27 (d, 2H, J = 7.4 Hz), 8.20 (d, 2H, J = 7.4 Hz), 8.08 (m, 4H), 7.83–7.68 (m, 12H), 7.65 (m, 8H), 7.53 (t, 2H, J = 7.4 Hz), 7.38 (m, 4H), 7.35 (s, 1H), 7.18 (s, 1H), 6.61 (br, 2H), 5.73 (br, 2H), 5.26 (br, 2H), 4.16 (m, 4H), 1.88–2.00 (m, 4H), 1.66–1.78 (m, 4H), 1.48–1.63 (m, 4H) 1.15–1.47 (m, 28H), 0.83 (t, 3H, J = 6.9 Hz), 0.81 (t, 3H, J = 6.9 Hz), 0.61 (br, 6H); ^{13}C NMR (150 MHz, THF-d_8) δ179.0, 162.6, 155.2, 154.6, 151.4, 150.8, 150.7, 150.6, 148.6, 144.3, 144.0, 138.9, 137.9, 135.3, 134.9, 132.7, 132.1, 132.0, 131.8, 131.2, 128.0, 127.9, 127.8, 127.6, 127.2, 127.1, 126.9, 126.8, 126.7, 125.0, 121.4, 121.1, 120.9, 117.7, 117.5, 117.1, 115.6, 113.5, 94.2, 93.5, 92.2, 88.5, 70.1, 70.0, 32.7, 32.6, 30.6, 30.5, 30.4, 30.4, 30.4, 30.3, 30.2, 30.1, 30.1, 27.1, 26.8, 23.4, 23.3, 20.1, 14.3, 14.2; IR (ATR): ν 3244, 3043, 2918, 2847, 2196, 1798, 1677, 1599, 1594, 1508, 1480, 1437, 1410, 1338, 1336, 1268, 1202, 1175, 1066, 992, 948, 901 cm^{-1}; HRMS (ESI$^+$): calcd. for $C_{142}H_{123}N_{12}O_5Zn_2$ *m/z* 2203.8317 [M+H]$^+$, found *m/z* 2203.8274.

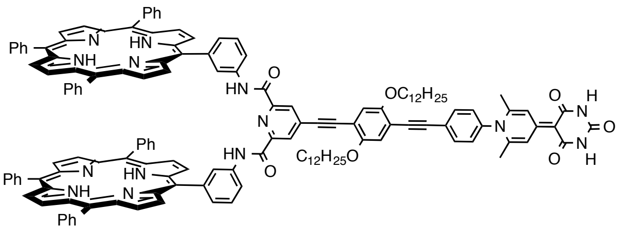

Barbituric bisporphyrin **2**: To a solution of **27** (135mg, 61.4 μmol) in acetic anhydride (4 ml) and glacial acetic acid (40 μl) was added barbituric acid **15** (7.9 mg, 61.4 μmol). After being stirred for 11 hours at 90 °C under argon atmosphere, the reaction mixture was added water (10 ml). After being filtered, the precipitate was dissolved in chloroform. TFA (0.5 mg, 6.5 mmol) was added to the solution. After being stirred for 30 minutes at room temperature, the organic layer was washed with saturated aqueous $NaHCO_3$ and brine, dried over Na_2SO_4, and concentrated *in vacuo*. The crude product was purified by GPC to give desired product **2** (95.4 mg, 71%) as a purple solid. M.p.: >300 °C; ^{1}H NMR (600 MHz, THF-d_8): δ 11.34 (s, 2H), 8.79 (br, 4H), 8.71 (br, 8H), 8.59 (s, 2H), 8.52–8.58 (m, 6H), 8.44 (s, 2H), 8.29 (d, 2H, *J* = 8.4 Hz), 8.16 (d, 2H, *J* = 8.4 Hz), 8.07 (d, 4H, *J* = 8.5 Hz), 7.59–7.80 (m, 20H), 7.57 (t, 2H, *J* = 8.4 Hz), 7.30 (s, 1H), 7.28 (br, 4H), 7.10 (s, 1H), 6.01 (br, 2H), 5.37 (br, 2H), 4.78 (br, 2H), 4.11 (m, 4H), 1.93 (m, 2H), 1.85 (m, 2H), 1.04–1.76 (m, 36H), 0.78 (t, 3H, *J* = 7.0 Hz), 0.71 (t, 3H, *J* = 7.0 Hz), 0.10 (br, 6H), –2.91 (s, 4H); ^{13}C NMR (150 MHz, THF-d_8): δ 179.3, 162.7, 155.1, 154.5, 151.3, 148.1, 142.9 142.7, 138.3, 138.0, 135.3, 135.1, 135.0, 134.8, 131.9, 131.3, 128.5, 128.3, 127.8, 127.5, 127.3, 124.3, 121.5, 121.0, 120.6, 120.4, 117.6, 117.5, 117.2, 115.4, 113.6, 93.9, 93.5, 92.3, 88.3, 70.1, 70.0, 32.6, 32.6, 30.6, 30.4, 30.4, 30.3, 30.2, 30.1, 27.1, 26.8, 23.3, 23.3, 19.4, 14.2; IR (ATR): *ν* 3267, 3021, 2918, 2846, 2199, 1752, 1652, 1645, 1591, 1594, 1520, 1486, 1477, 1465, 1355, 1260, 1202, 1174, 1067, 992, 984, 838 cm^{-1}; HRMS (ESI^{+}): calcd. for $C_{146}H_{130}N_{14}O_7$ *m*/*z* 1095.5118 $[M+2H]^{+}$, found *m*/*z* 1095.5137.

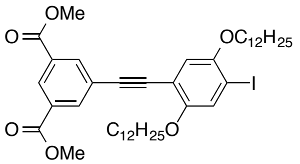

Dimethyl 5-[{2,5-bis(dodecyloxy)-4-iodophenyl}ethynyl]isophthalate (**28**): To a solution of **17** (13.7 g, 19.6 mmol) in dry THF (65 ml) was added CuI (124 mg, 0.653 mmol) and dry diisopropylamine (2.3 ml, 16 mmol). After being stirred at room temperature for 15 minutes under argon atmosphere, **16** (1.53 g, 6.53 mmol) was added. $Pd(PPh_3)_2Cl_2$ (230 mg, 0.327 mmol) was added to the resultant solution. After being stirred at room temperature for 20 hours under argon atmosphere in the dark, the reaction mixture was passed through florisil column, and diluted with ethyl acetate. The organic layer was washed with saturated aqueous NH_4Cl, brine, dried over Na_2SO_4, and concentrated *in vacuo*. The crude product was purified by column chromatography on silica gel (0–40% ethyl acetate in *n*-hexane) to give desired product **28** (4.63 g, 88%) as a yellow solid. M.p.: 46–47°C; ^{1}H NMR (300 MHz, $CDCl_3$): δ 8.62 (t, 1H *J* = 1.7 Hz), 8.36 (d, 2H *J*= 1.7 Hz), 7.32 (s, 1H), 6.91 (s, 1H), 4.00 (t, 2H, *J* = 6.6 Hz), 3.93–4.01 (m, 2H), 3.96 (s, 6H), 1.75–1.91 (m, 4H), 1.45–1.59 (m, 4H), 1.15–1.44 (m, 32H), 0.82–0.92 (m, 6H); ^{13}C NMR ($CDCl_3$, 75 MHz): δ^{13}C NMR (75 MHz, $CDCl_3$) δ = 165.6, 154.4, 151.8, 136.4, 130.9, 130.0, 124.5, 123.8, 115.9, 112.7, 92.0, 88.4, 87.6, 70.1, 69.9, 52.5, 31.9, 31.9, 29.7, 29.6, 29.6, 29.6, 29.6, 29.3, 29.3, 29.3, 29.2, 29.1, 26.0, 26.0, 22.7, 22.7, 14.1, 14.1; IR (ATR): *v* 2911, 2848, 2187, 1725, 1575, 1474, 1468, 1466, 1381, 1335, 1315, 1295, 1235, 1215, 1134, 1101, 1046, 997, 982, 908, 838 cm^{-1}; HRMS (ESI$^+$): calcd. for $C_{42}H_{61}IO_6Na$ *m*/*z* 811.3405 [M+Na]$^+$, found *m*/*z* 811.3411.

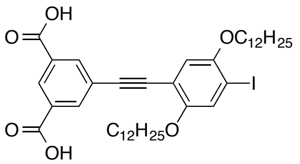

5-[{2,5-Bis(dodecyloxy)-4-iodophenyl}ethynyl]isophthalic acid (**29**): To a solution of **28** (3.65 g, 4.53 mmol) in THF (285 ml) and water (60 ml) was added NaOH (1.54 g, 38.5 mmol). After being stirred for 24 hours at room temperature under argon atmosphere, the reaction mixture was acidified with 3M hydrochloric acid to pH 1. After extraction with ethyl acetate, the organic layer was washed with brine, dried over Na_2SO_4, and concentrated *in vacuo* to give desired product **29** (1.98 g, 55%) as a yellow solid. M.p.: 135–137°C; ^{1}H NMR (300 MHz, THF-d_8): δ 8.61 (t, 1H *J*= 1.7 Hz), 8.30 (d, 2H *J*= 1.7 Hz), 7.42 (s, 1H), 7.05 (s, 1H), 4.03 (t, 2H, *J*= 6.5 Hz), 3.99 (t, 2H, *J*= 6.5 Hz), 1.76–1.88 (m, 4H), 1.49–1.65 (m, 4H), 1.20–1.47 (m, 32H), 0.84–0.92 (m, 6H); ^{13}C NMR (75 MHz, THF-d_8): δ 166.1, 155.4, 152.8, 136.5, 132.6, 130.8, 125.1, 124.5, 116.3, 113.6, 92.6, 88.6, 88.1, 70.4, 70.2, 32.7, 30.4, 30.1, 30.0, 26.9, 23.4, 14.3; IR (ATR): *v* 3633, 2912, 2848, 2187, 1705, 1584, 1474, 1426, 1376, 1286, 1262, 1229, 1144, 1096, 1046, 904, 854, 754 cm^{-1}; HRMS (ESI$^+$): calcd. for $C_{40}H_{58}IO_6$ *m*/*z* 761.3273 [M+H]$^+$, found *m*/*z* 761.3284.

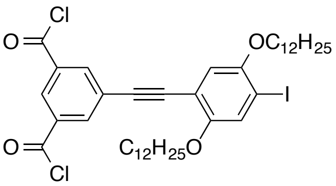

5-[{2,5-Bis(dodecyloxy)-4-iodophenyl}ethynyl]isophthaloyl dichloride (**18**): To a solution of **29** (1.98 g, 2.60 mmol) and dry DMF (32μl, 0.032 mmol) in dry THF (160 ml) was added oxalyl chloride (3.0 ml, 35 mmol) at 0°C. After being stirred for 2 hours at room temperature under argon atmosphere, the reaction mixture was concentrated *in vacuo* to give acid chloride **18** (2.0 g, 96%). M.p.: 93-95°C; ^{1}H NMR (300 MHz, $CDCl_3$): δ 8.70 (t, 1H, J = 1.7 Hz), 8.49 (d, 1H, J = 1.7 Hz), 7.34 (s, 1H), 6.92 (s, 1H), 4.01 (t, 2H, J = 7.0 Hz), 3.98 (t, 2H, J = 7.0 Hz), 1.90–1.77 (m, 4H), 1.60–1.45 (m, 4H), 1.44–1.18 (m, 32H), 0.83–0.93 (m, 6H); ^{13}C NMR (125 MHz, $CDCl_3$): δ 170.9, 154.3, 151.6, 137.2, 130.6, 129.7, 127.9, 125.0, 123.5, 115.4, 112.6, 91.6, 88.6, 69.9, 69.7, 32.0, 31.9, 29.8, 29.7, 29.7, 29.6, 29.6, 29.5, 29.4, 29.4, 29.3, 26.2, 26.2, 26.1, 22.7, 22.7, 14.1, 14.1; IR (ATR): *v* 2919, 2849, 2206, 1744, 1590, 1464, 1440, 1384, 1372, 1323, 1264, 1103, 1012, 907, 852, 755 cm^{-1}; HRMS ($APCI^+$) calcd. for $C_{40}H_{56}ICl_2O_4$ *m/z* 797.2595 $[M+H]^+$, found *m/z* 797.2581.

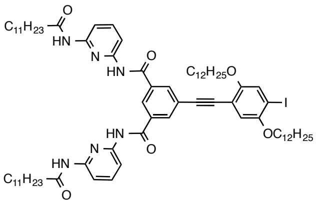

5-[{2,5-Bis(dodecyloxy)-4-iodophenyl}ethynyl]-N^1,N^3-bis(6-dodecanamidopyridin-2-yl)isophthalamide (**20**): To a solution of crude **18** (2.00 g, 2.51 mmol) in dry THF (220 ml) was added **19** (2.27 mg, 7.80 mmol) and triethylamine (4.5 ml, 32 mmol). After being stirred for 10 hours at room temperature under argon atmosphere, the solution was concentrated *in vacuo*. The crude product was purified by column chromatography on silica gel (0%–30% ethyl acetate in *n*-hexane) to give desired product **20** (2.54 g, 75%) as a yellow solid. M.p.: 74–76 °C; ^{1}H NMR (300 MHz, $CDCl_3$): δ 8.42 (bs, 2H), 8.35 (bs, 1H), 8.19 (d, 2H, J = 1.6 Hz), 8.04 (d, 2H, J = 8.3 Hz), 7.99 (d, 2H, J = 8.3 Hz), 7.76 (t, 2H, J = 8.3 Hz), 7.69–7.82 (m, 2H), 7.33 (s, 1H), 6.90 (s, 1H), 4.01 (t, 2H, J = 6.3 Hz), 3.97 (t, 2H, J = 6.3 Hz), 2.40 (t, 4H, J = 7.3 Hz), 1.60–1.90 (m, 8H), 1.09–1.57 (m, 68H), 0.79–0.93 (m, 12H) ppm; ^{13}C NMR (125 MHz, $CDCl_3$): δ 172.1, 163.8, 154.3, 151.8, 149.9, 149.1, 140.7, 134.7, 133.5, 125.2, 123.8, 115.8, 112.3, 110.2, 109.6, 91.6, 88.8, 88.7, 70.1, 69.8, 37.6, 31.9, 31.8, 29.6, 29.5, 29.4, 29.3, 29.2, 29.2, 29.1, 26.1, 25.9, 25.3, 22.6, 14.0; IR (ATR): *v* 3264, 2918, 2849, 2187, 1684, 1668, 1652, 1583, 1523, 1505, 1467, 1446, 1374, 1314, 1291, 1241, 1212, 1152, 1015, 851, 797 cm^{-1}; HRMS (ESI^+): calcd. for $C_{74}H_{111}IN_6O_6Na$ *m/z* 1329.7502 $[M+Na]^+$, found *m/z* 1329.7509.

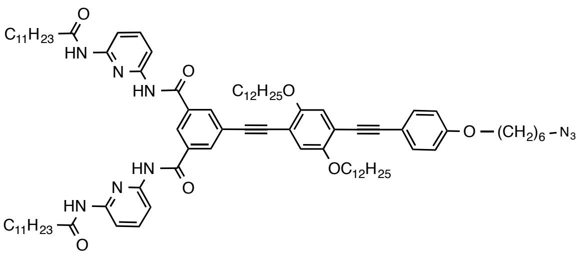

5-[{4-((4-((6-Azidohexyl)oxy)phenyl)ethynyl)-2,5-bis(dodecyloxy)phenyl}ethynyl]-*N*[1],*N*[3]-bis(6-dodecanamidopyridin-2-yl)isophthalamide (**21**): To a solution of **20** (295 mg, 0.230 mmol) in dry THF (5.0 ml) was added CuI (2.1 mg, 0.011 mmol) and dry diisopropylamine (81 μl, 0.58 mmol). After being stirred at room temperature for 15 minutes under argon atmosphere, **7** (83.9 mg, 0.350 mmol) was added. $Pd(PPh_3)_2Cl_2$ (16.1mg, 22.9 μmol) was added to the solution. After being stirred at room temperature for 15 hours under argon atmosphere in the dark, the reaction mixture was passed through florisil column, and diluted with ethyl acetate. The organic layer was washed with saturated aqueous NH_4Cl and brine, dried over Na_2SO_4 and concentrated *in vacuo*. The crude product was purified by column chromatography on silica gel (0%–30% ethyl aceate in *n*-hexane). Reprecipitation (methanol-chloroform) gave desired product **21** (177 mg, 54%) as a yellow solid. M.p.: 78–80 °C; ^{1}H NMR (300 MHz, $CDCl_3$): δ 8.46 (s, 2H), 8.36 (s, 1H), 8.19 (s, 2H), 8.04 (d, 2H, *J* = 8.2 Hz), 7.98 (d, 2H, *J* = 8.2 Hz), 7.69–7.80 (m, 4H), 7.46 (d, 2H, *J* = 8.8 Hz), 7.01 (s, 1H), 6.98 (s, 1H), 6.86 (d, 2H, *J* = 8.8 Hz), 3.91–4.09 (m, 6H), 3.29 (t, 2H, *J* = 7.4 Hz), 2.40 (t, 4H, *J* = 7.2 Hz), 1.00–1.90 (m, 84H), 0.79–0.91 (m, 12H) ppm; ^{13}C NMR (75 MHz, $CDCl_3$): δ 171.8, 163.6, 159.3, 153.8, 153.4, 149.8, 149.1, 140.9, 135.0, 133.3, 133.1, 125.5, 125.1, 117.0, 116.7, 115.5, 115.2, 114.5, 112.1, 110.1, 109.6, 95.7, 92.1, 89.1, 84.4, 69.7, 67.8, 51.4, 37.8, 31.9, 29.7, 29.5, 29.4, 29.3, 29.2, 29.1, 28.8, 26.5, 26.1, 26.0, 25.7, 25.3, 22.7, 14.1; IR (ATR): ν 3264, 2918, 2847, 2187, 2089, 1664, 1583, 1511, 1444, 1412, 1379, 1288, 1240, 1223, 1158, 1151, 1061, 1034, 1013, 990, 902, 807, 798 cm^{-1}; HRMS (ESI^-): calcd. for $C_{88}H_{126}O_7N_9$ *m/z* 1420.9786 $[M–H]^-$, found *m/z* 1420.9748.

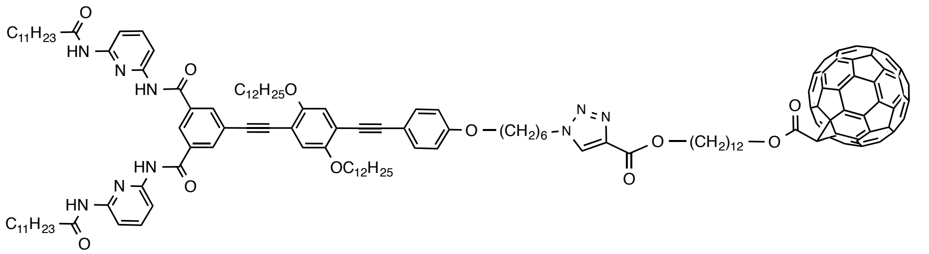

Fullereno isophthaldiamide **3**: To a solution of **21** (600 mg, 0.422 mmol) in dry dichloromethane (15 ml) and distilled water (12 ml) was added **22** (300 mg, 0.296 mmol), $CuSO_4$ (94.5 mg, 0.592 mmol) and sodium ascorbate (117 mg, 0.592 mmol). After being stirred at 20 °C for two days under argon atmosphere, the reaction mixture was diluted with dichloromethane. The organic layer was dried over Na_2SO_4, and concentrated *in vacuo*. The crude product was purified by column chromatography on silica gel (0%–100% ethyl acetate in *n*-hexane, then 20% methanol in chloroform). The mixture was further purified by column chromatography on silica gel (0%–30% ethyl acetate in benzene) to give desired product **3** (185 mg, 27%) as a blown solid. M.p.: >165 °C (dec.); ^{1}H NMR (600 MHz, THF-d_6): δ 9.64 (s, 2H), 9.04 (s, 1H), 8.42 (s, 1H), 8.37 (s, 1H), 8.24 (s, 2H), 8.03 (d, 2H, J = 7.3 Hz), 8.02 (d, 2H, J = 7.3 Hz), 7.72 (t, 2H, J = 7.3 Hz), 7.42 (d, 2H, J = 8.1 Hz), 7.10 (s, 1H), 7.08 (s, 1H), 6.90 (d, 2H, J = 8.1 Hz), 5.21 (s, 1H), 4.44 (t, 2H, J = 7.3 Hz), 4.42 (t, 2H, J = 7.3 Hz), 4.26 (t, 2H, J = 6.7 Hz), 4.07 (t, 2H, J = 6.7 Hz), 4.05 (t, 2H, J = 6.7 Hz), 3.99 (t, 2H, J = 6.4 Hz), 2.37 (t, 4H, J = 7.0 Hz), 1.96 (m, 2H), 1.20–1.90 (m, 102H), 0.82–0.92 (m, 12H); ^{13}C NMR (150 MHz, THF-d_6): δ171.8, 166.2, 164.8, 161.3, 160.2, 154.8, 154.3, 151.7, 151.1, 149.7, 147.3, 146.4, 145.9, 145.8, 145.7, 145.7, 145.6, 145.4, 145.3, 145.2, 145.2, 145.0, 144.9, 144.5, 144.3, 143.8, 143.7, 143.6, 143.5, 143.4, 143.1, 142.8, 142.7, 142.7, 141.6, 141.4, 141.2, 140.6, 140.4, 137.1, 136.5, 134.5, 134.0, 133.5, 128.5, 126.8, 125.3, 117.5, 117.3, 116.2, 115.1, 113.3, 110.2, 110.0, 95.9, 93.3, 89.0, 85.3, 72.2, 70.1, 70.0, 68.3, 65.0, 50.6, 40.3, 37.5, 32.7, 30.8, 30.5, 30.4, 30.4, 30.4, 30.3, 30.3, 30.3, 30.2, 30.2, 30.2, 30.1, 30.1, 30.1, 30.0, 29.7, 29.5, 29.5, 27.0, 26.9, 26.9, 26.8, 26.3, 26.0, 23.4, 14.3, 14.3, 14.3; IR (ATR): ν 3255, 2918, 2847, 1705, 1582, 1507, 1445, 1368, 1283, 1240, 1233, 1224, 1181, 1150, 1073, 1034, 1011, 985, 807, 795 cm^{-1}; HRMS (ESI^{+}): calcd. for $C_{165}H_{154}O_{11}N_9$ *m/z* 2437.1762 $[M+H]^+$, found *m/z* 2437.1794.

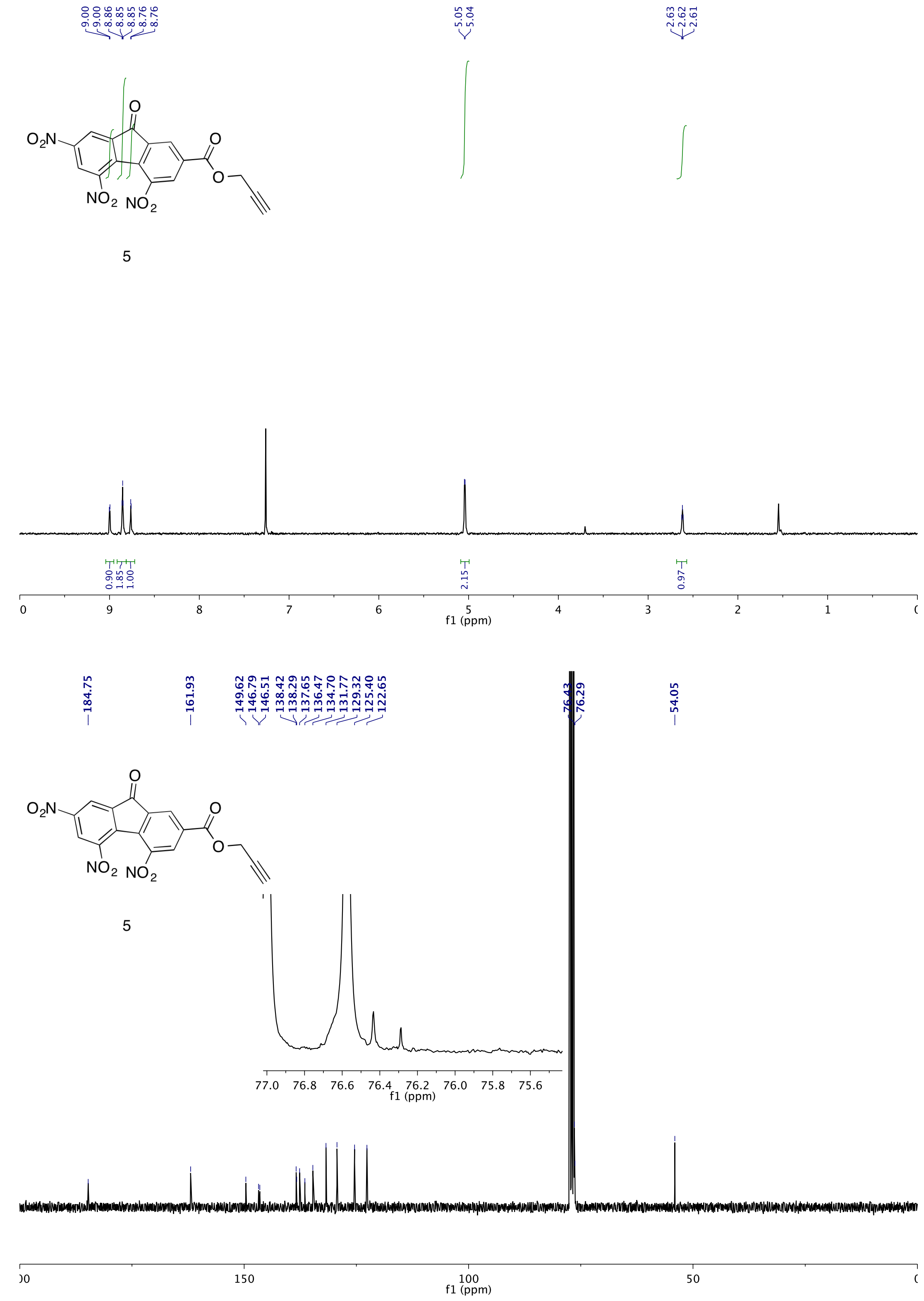

Supplementary Figure 61. ^{1}H and ^{13}C NMR spectra of **5** in chloroform-d_1.

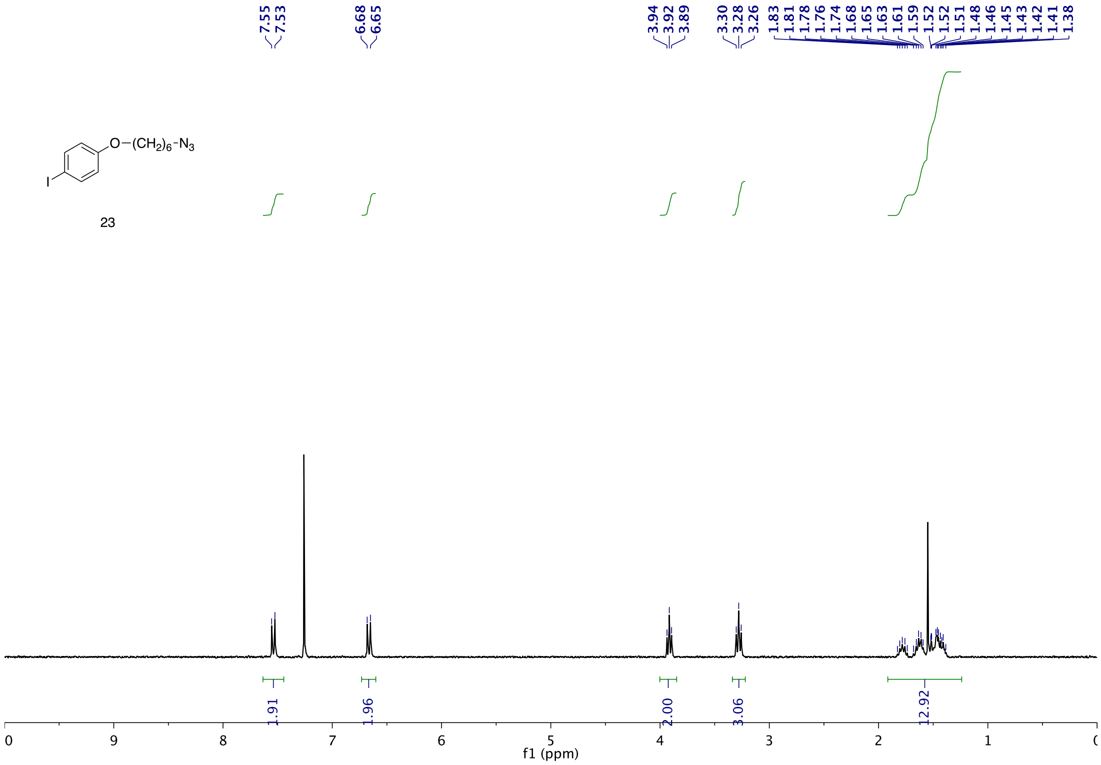

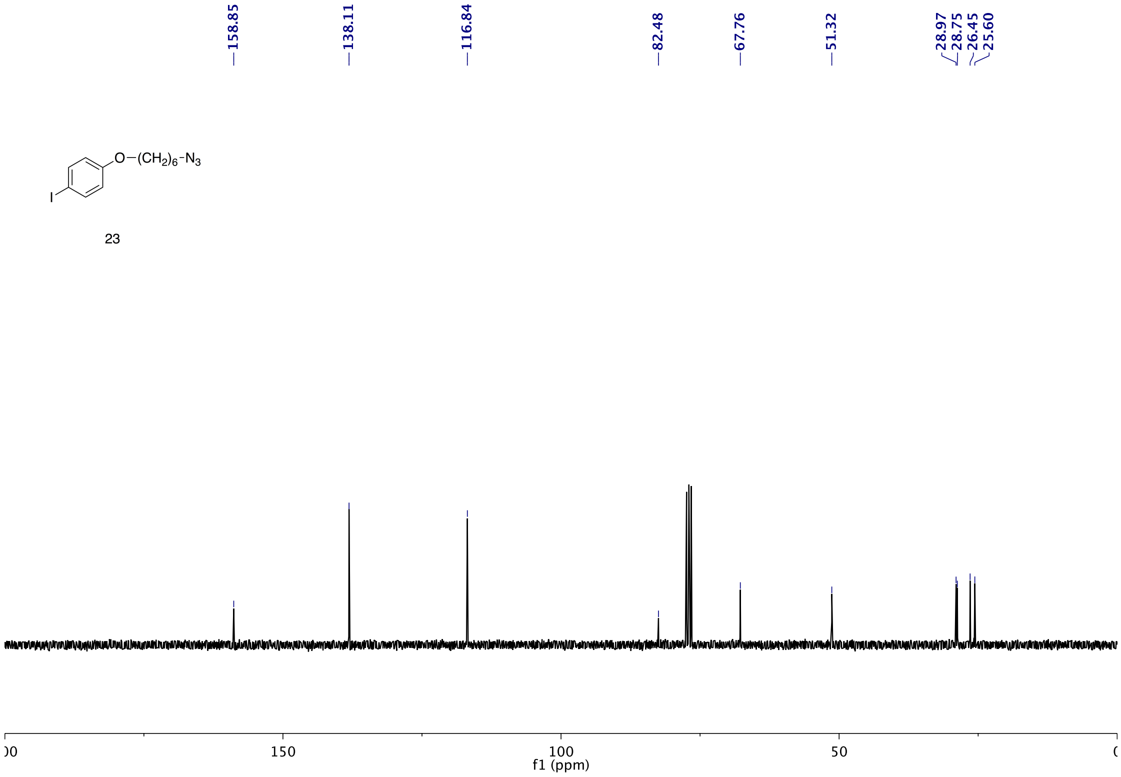

Supplementary Figure 62. ^{1}H and ^{13}C NMR spectra of **23** in chloroform-d_1.

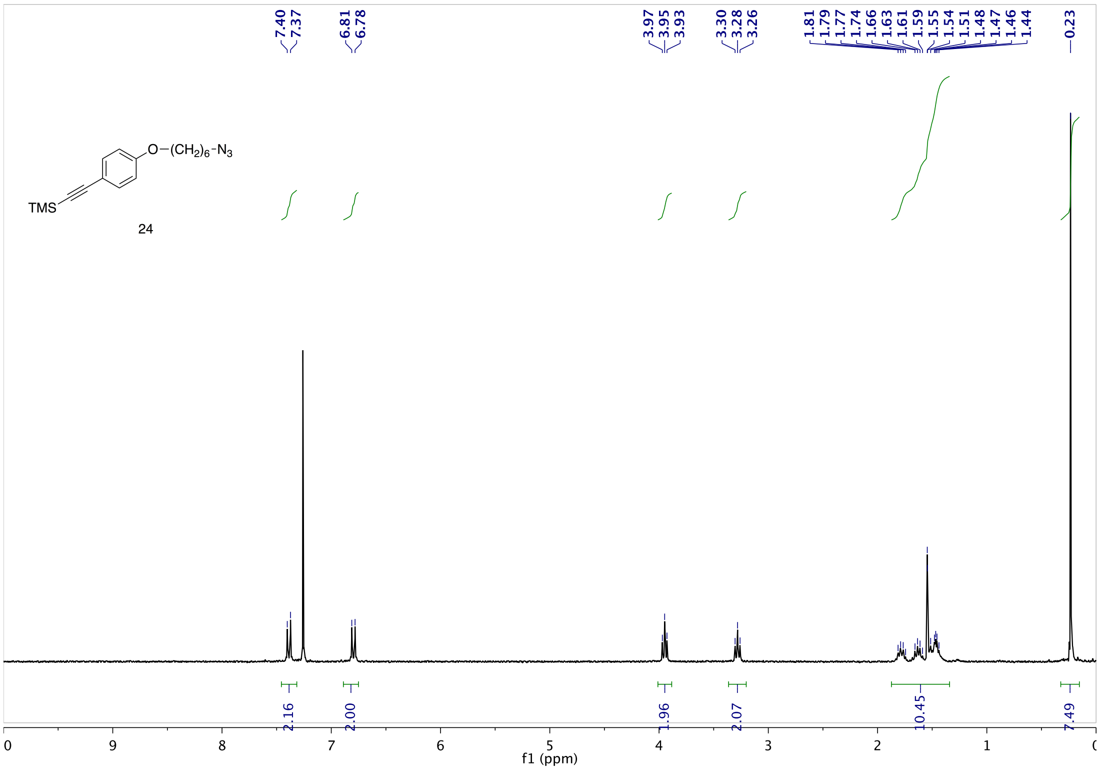

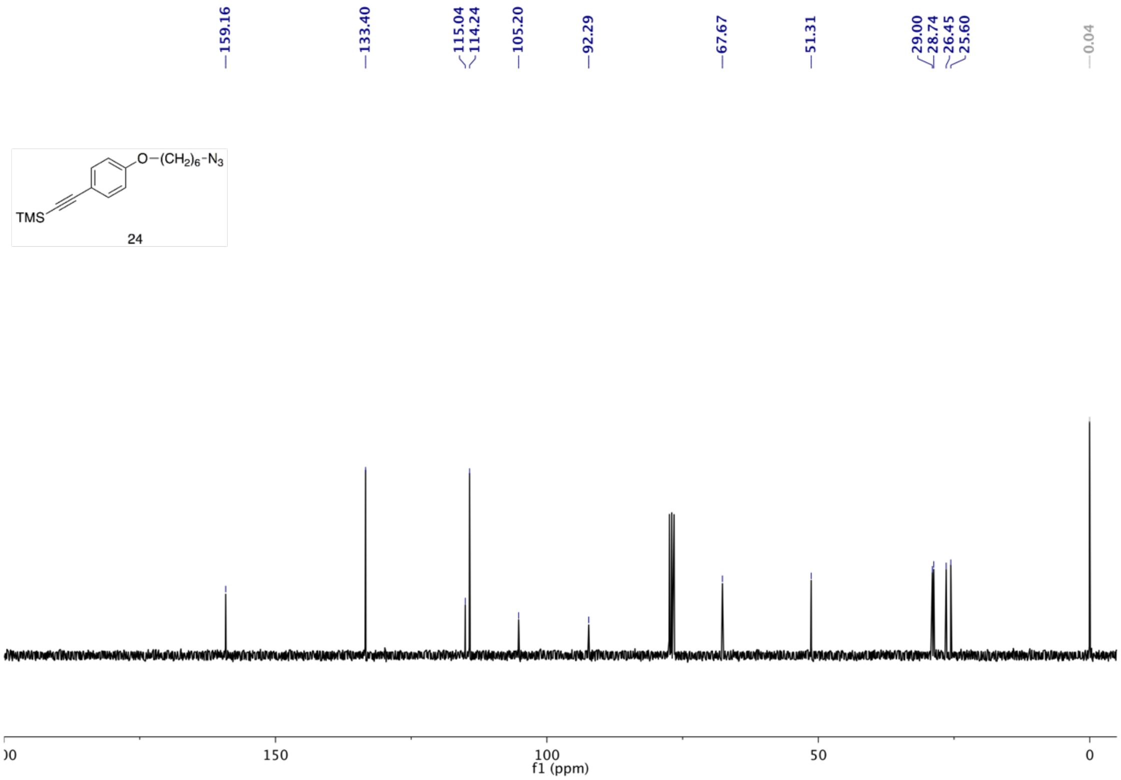

Supplementary Figure 63. ^{1}H and ^{13}C NMR spectra of **2** in chloroform-d_1.

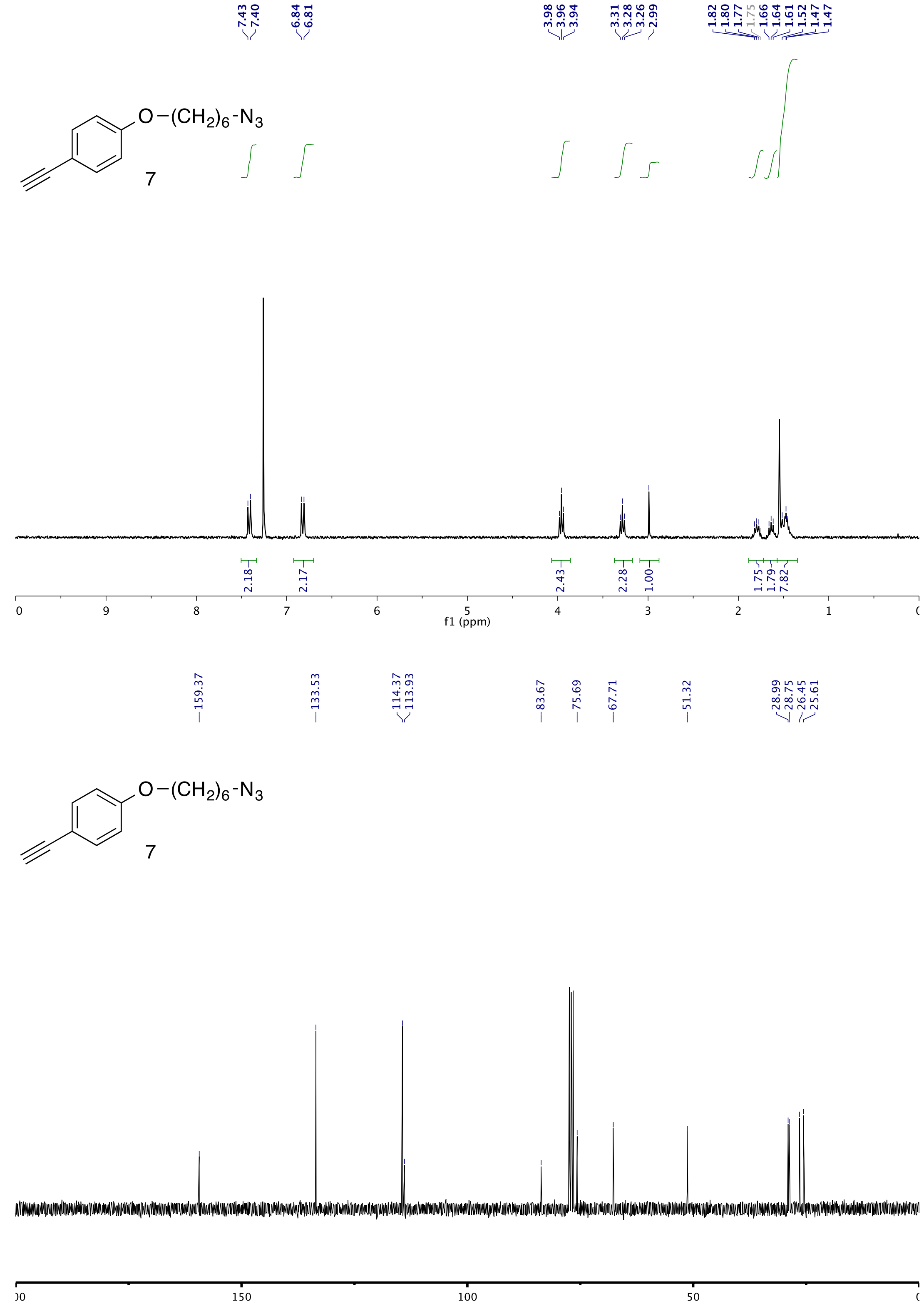

Supplementary Figure 64. ^{1}H and ^{13}C NMR spectra of **7** in chloroform-d_1.

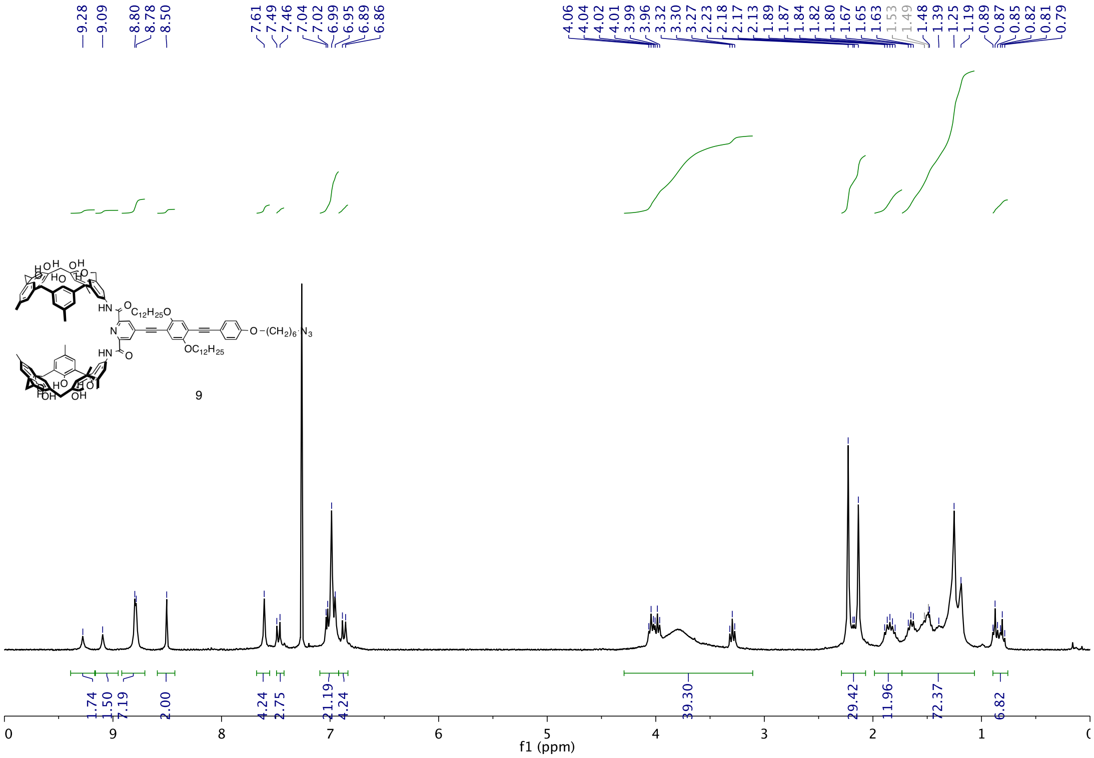

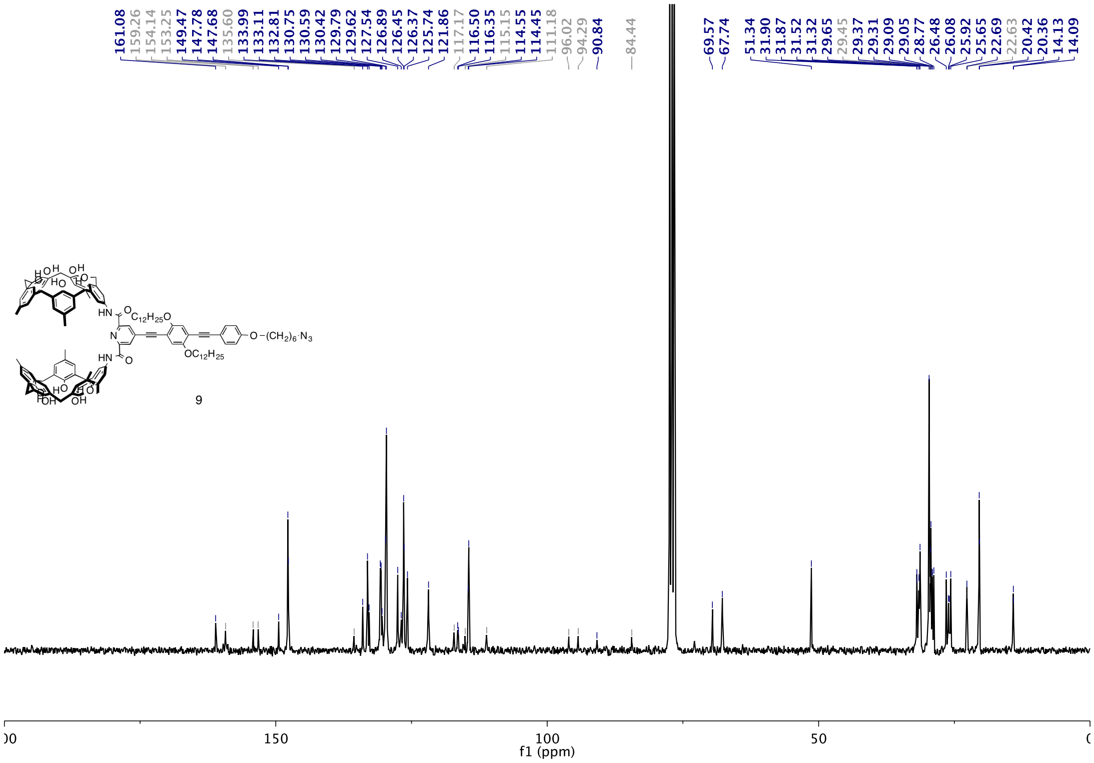

Supplementary Figure 65. ^{1}H and ^{13}C NMR spectra of **9** in chloroform-d_1.

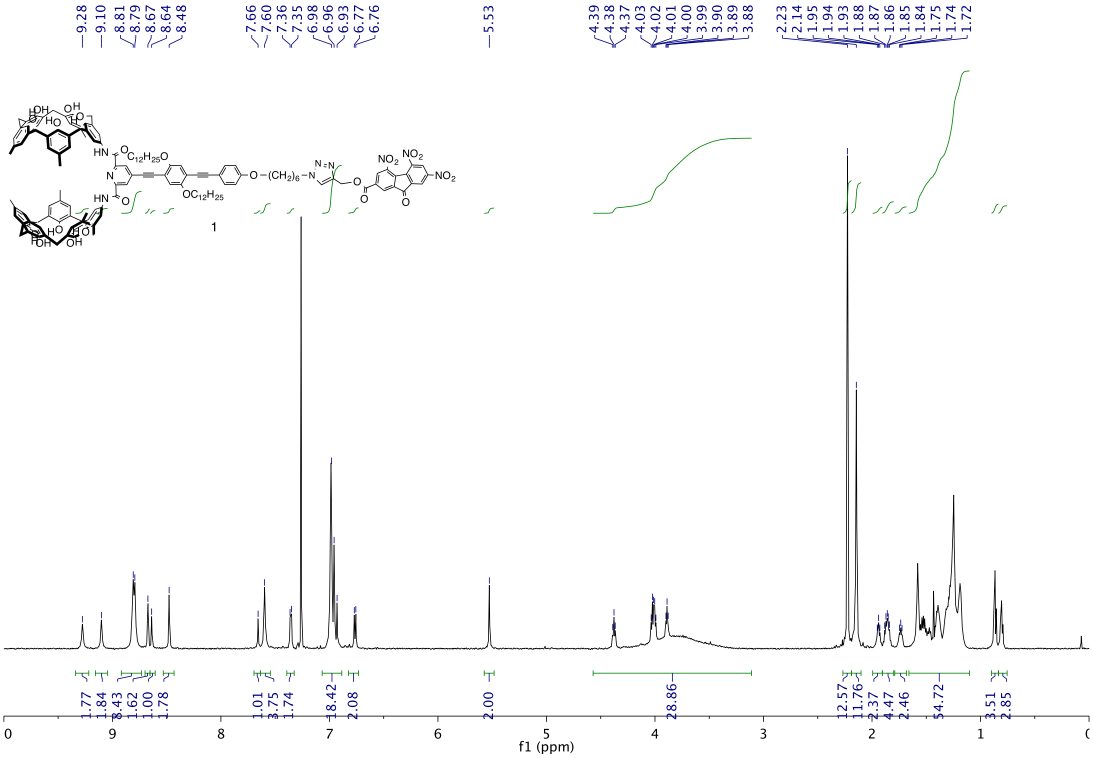

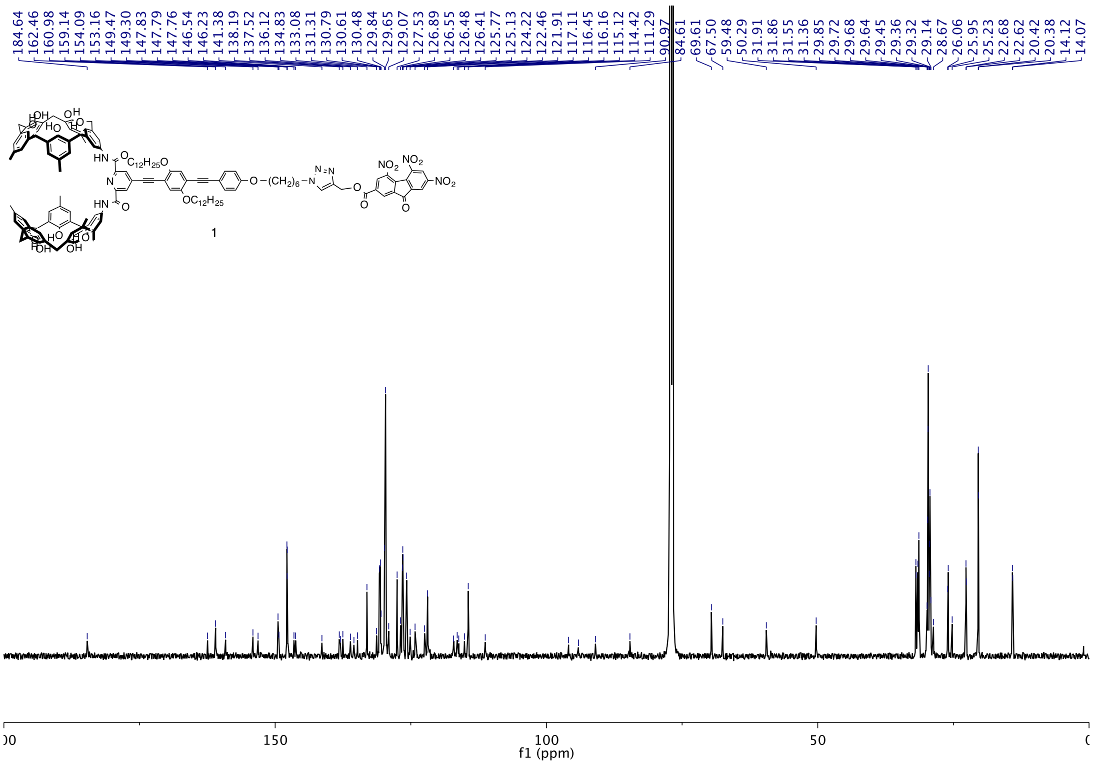

Supplementary Figure 66. ^{1}H and ^{13}C NMR spectra of **1** in chloroform-d_1.

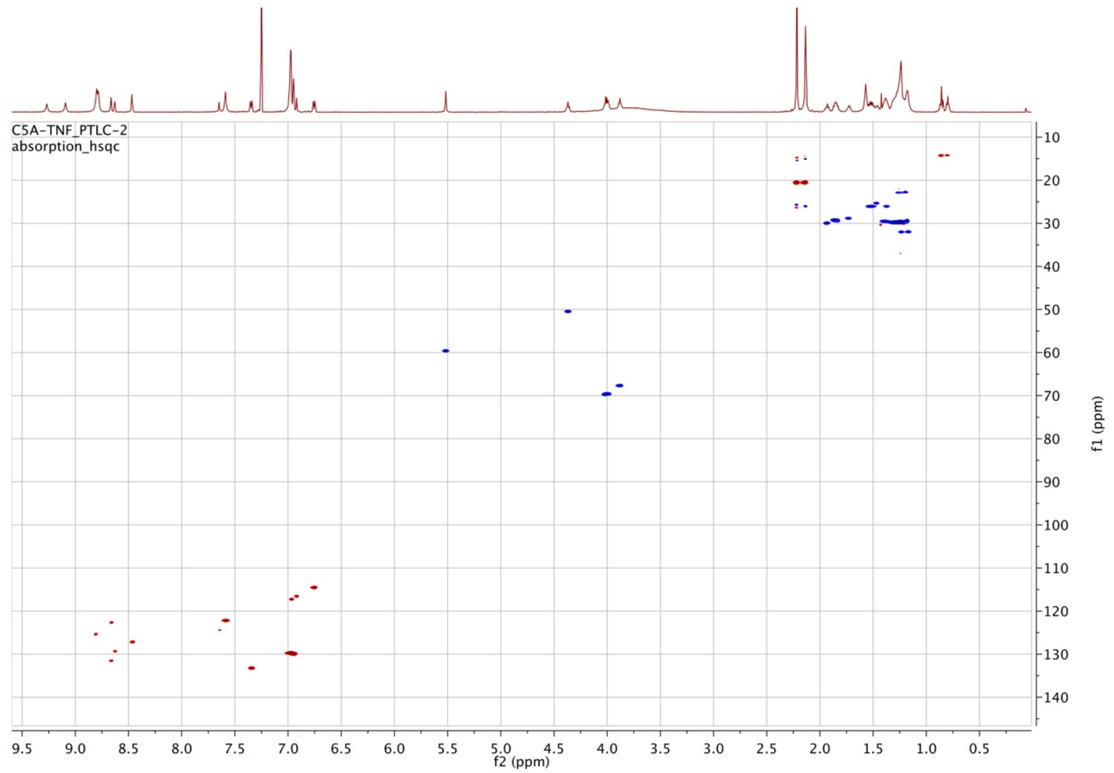

Supplementary Figure 67. HSQC spectrum of **1** in chloroform-d_1. CH_3 and CH peaks are phased up (red), and CH_2 carbons are phase down (blue).

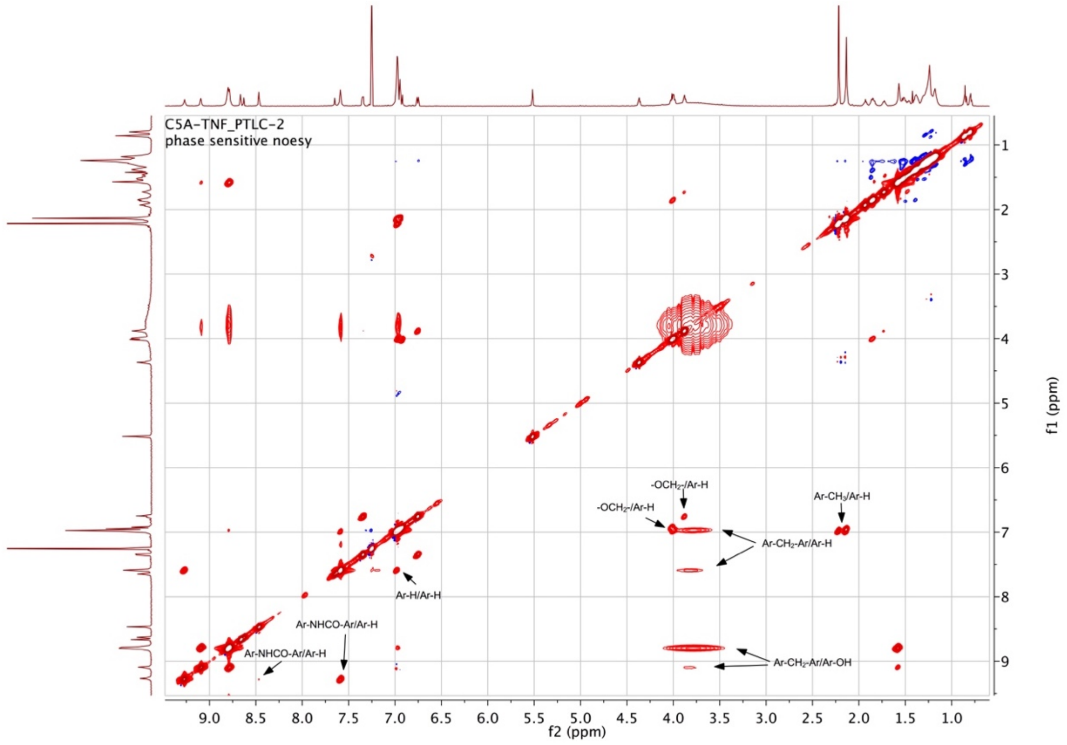

Supplementary Figure 68. NOESY spectra of **1** in chloroform-d_1.

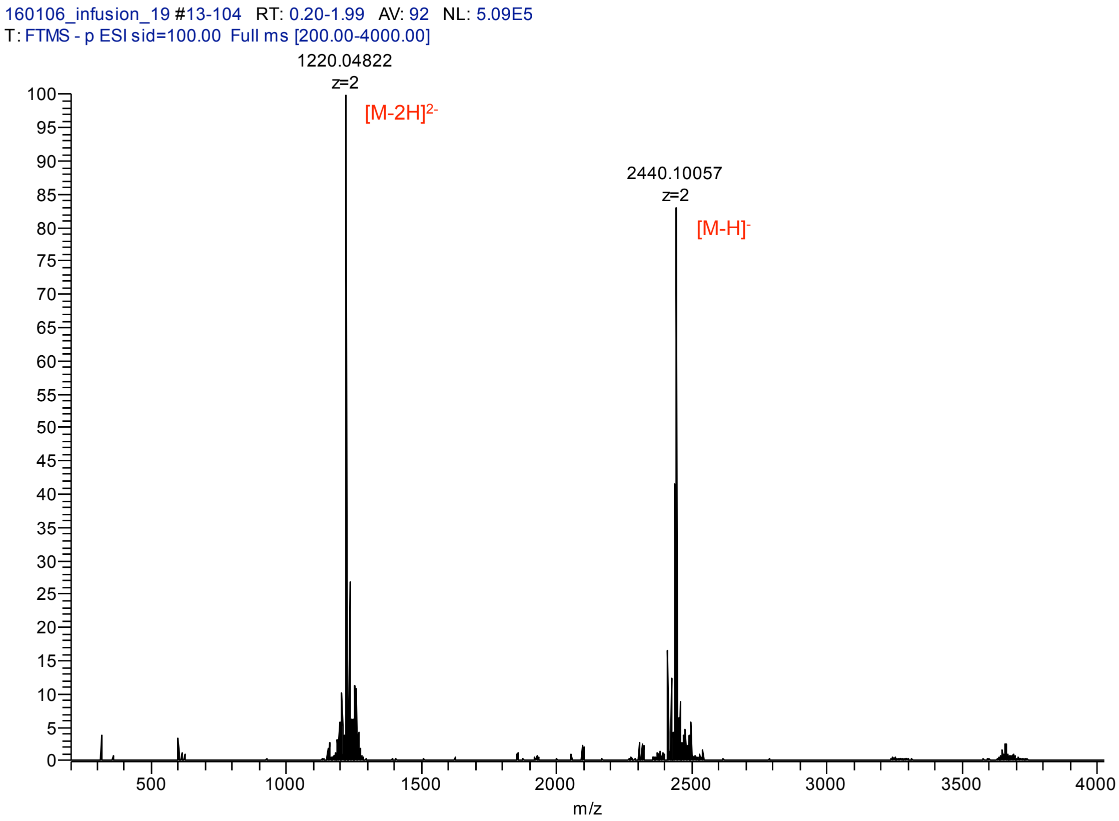

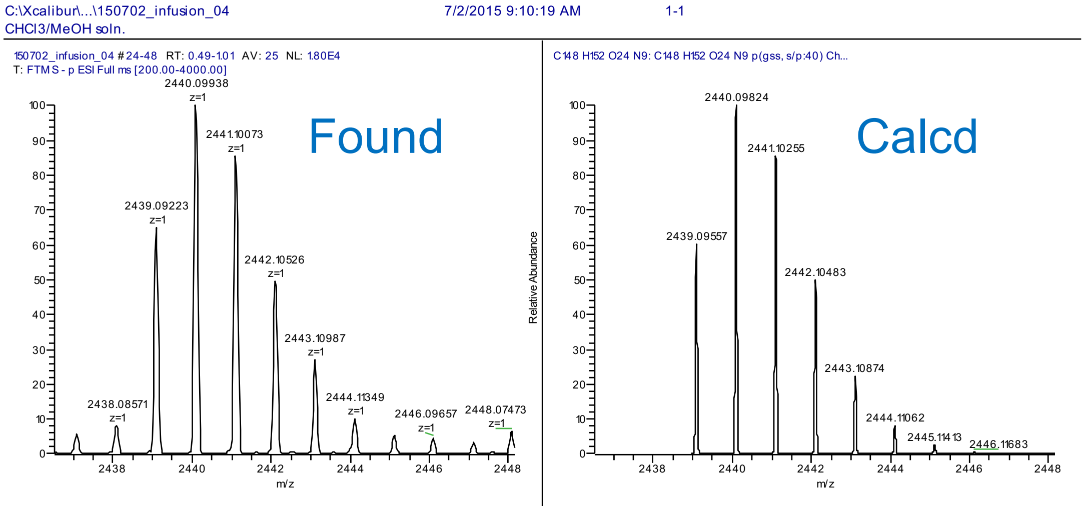

Supplementary Figure 69. MS spectra of **1**

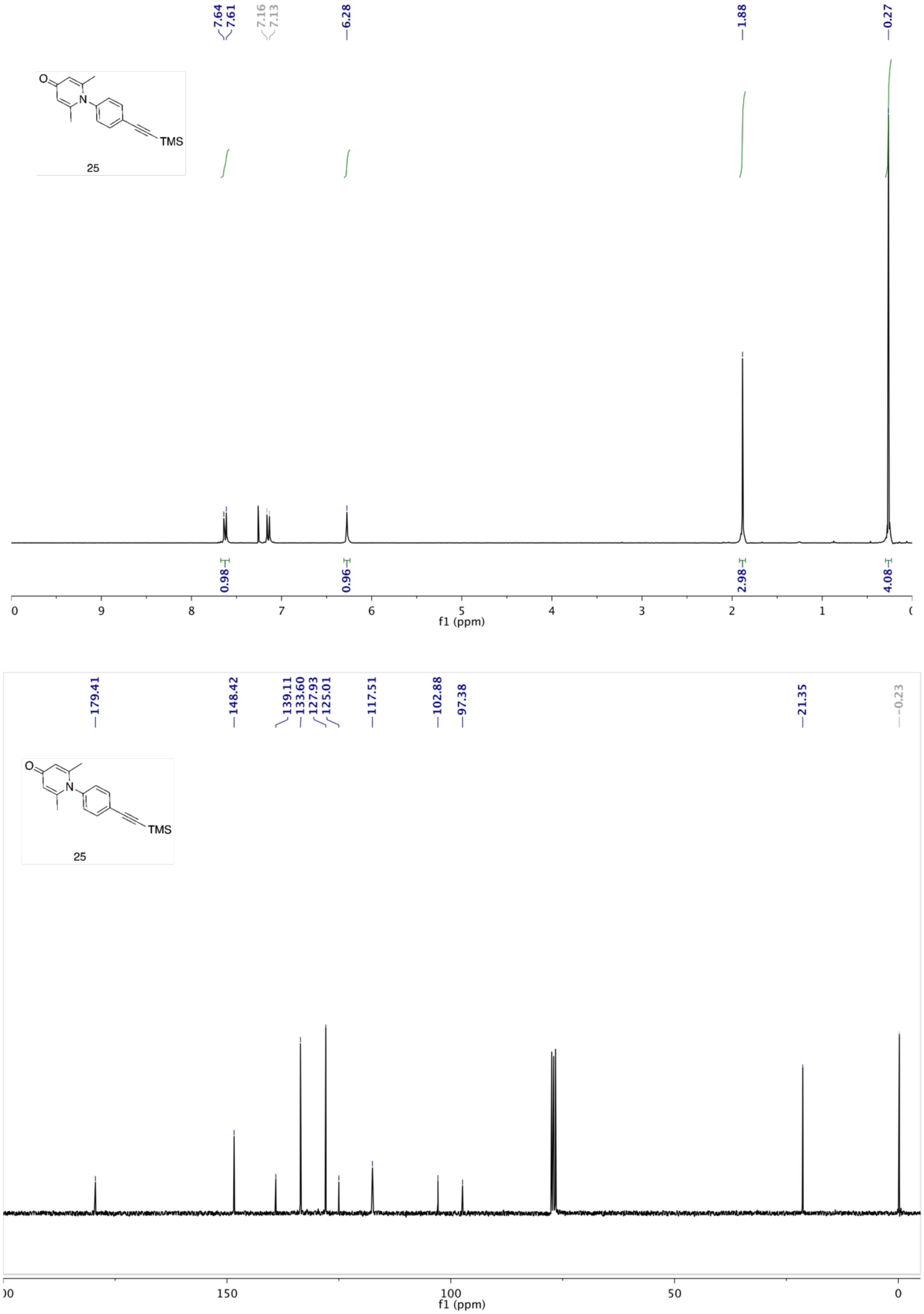

Supplementary Figure 70. ^{1}H and ^{13}C NMR spectra of **25** in chloroform-d_1.

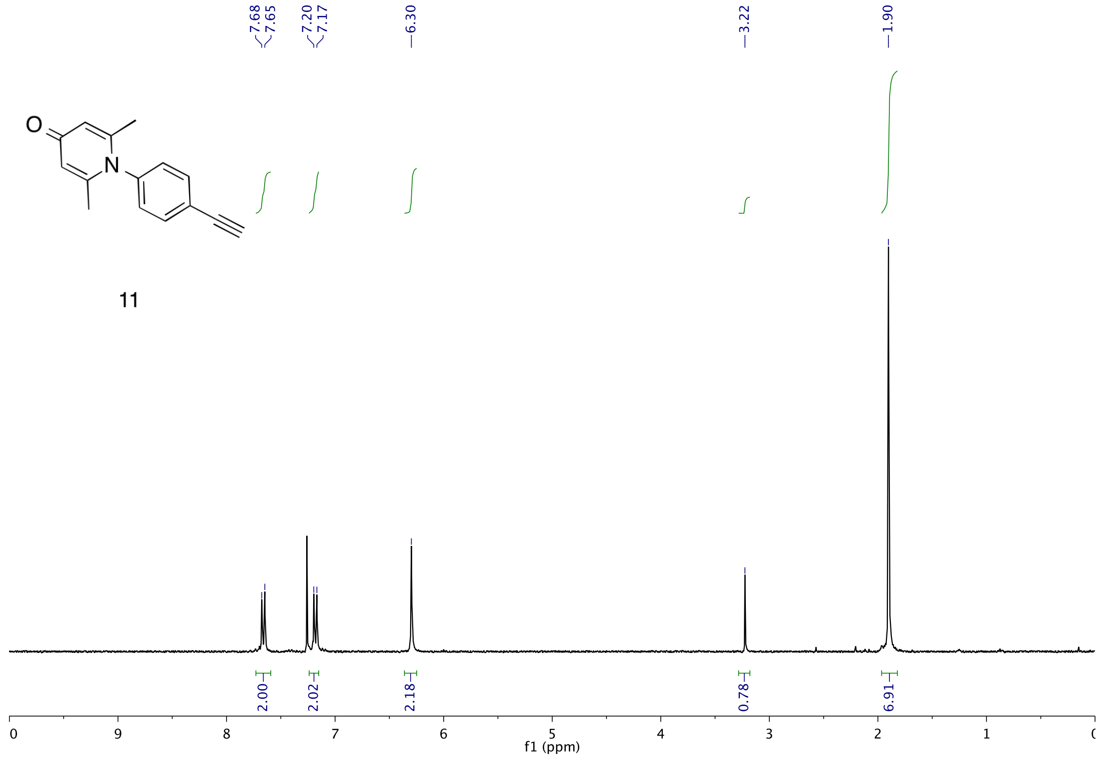

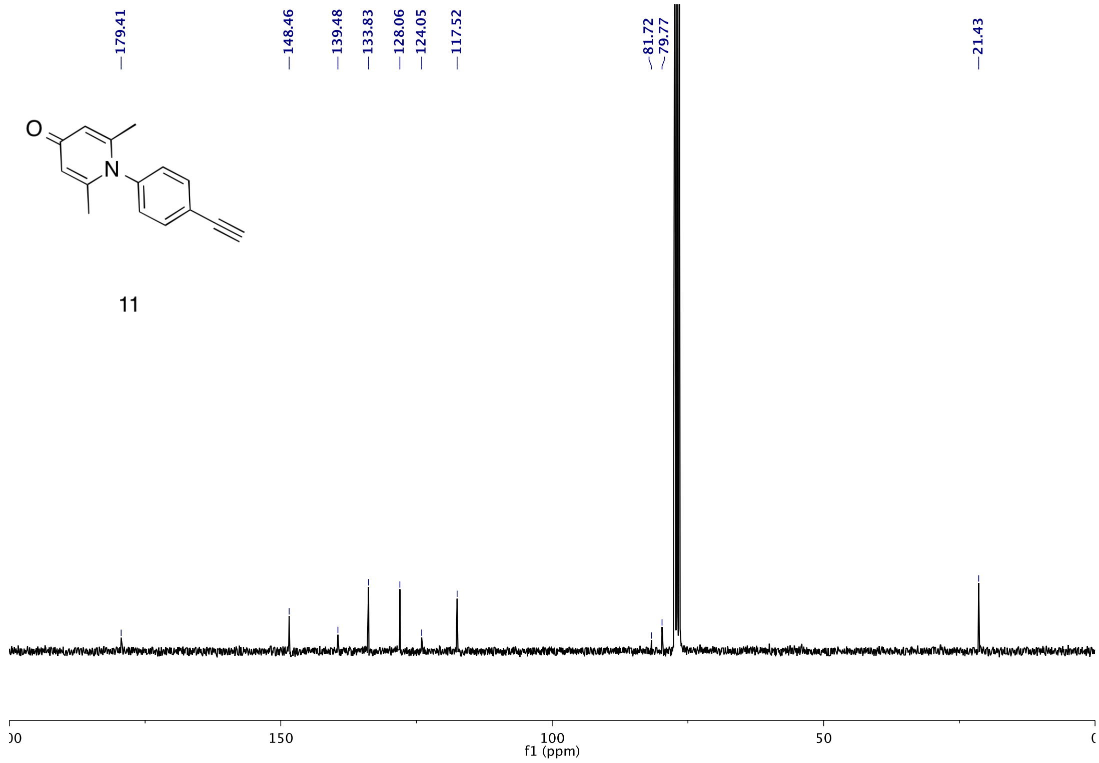

Supplementary Figure 71. ^{1}H and ^{13}C NMR spectra of **11** in chloroform-d_1.

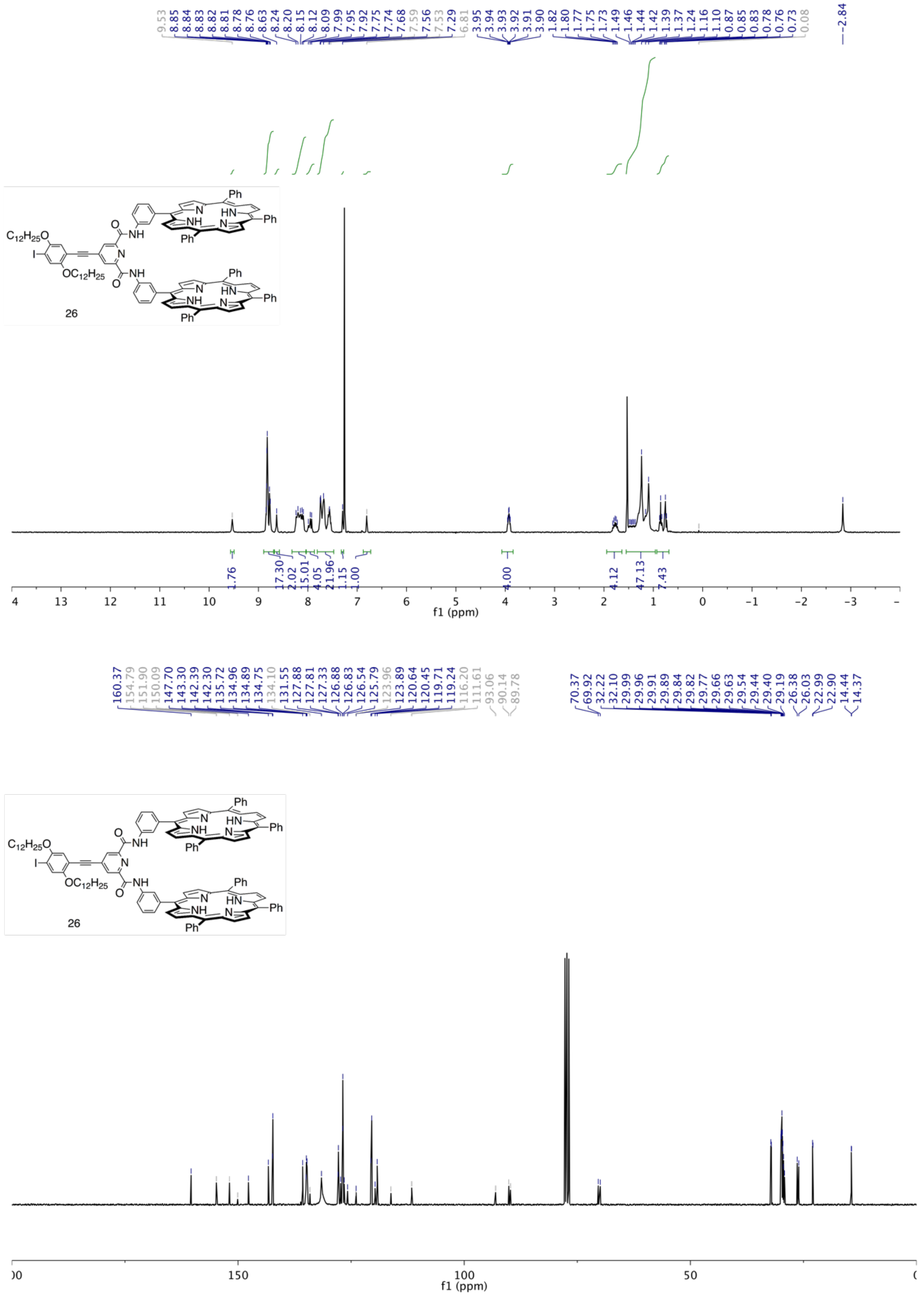

Supplementary Figure 72. ^{1}H and ^{13}C NMR spectra of **26** in chloroform-d_1.

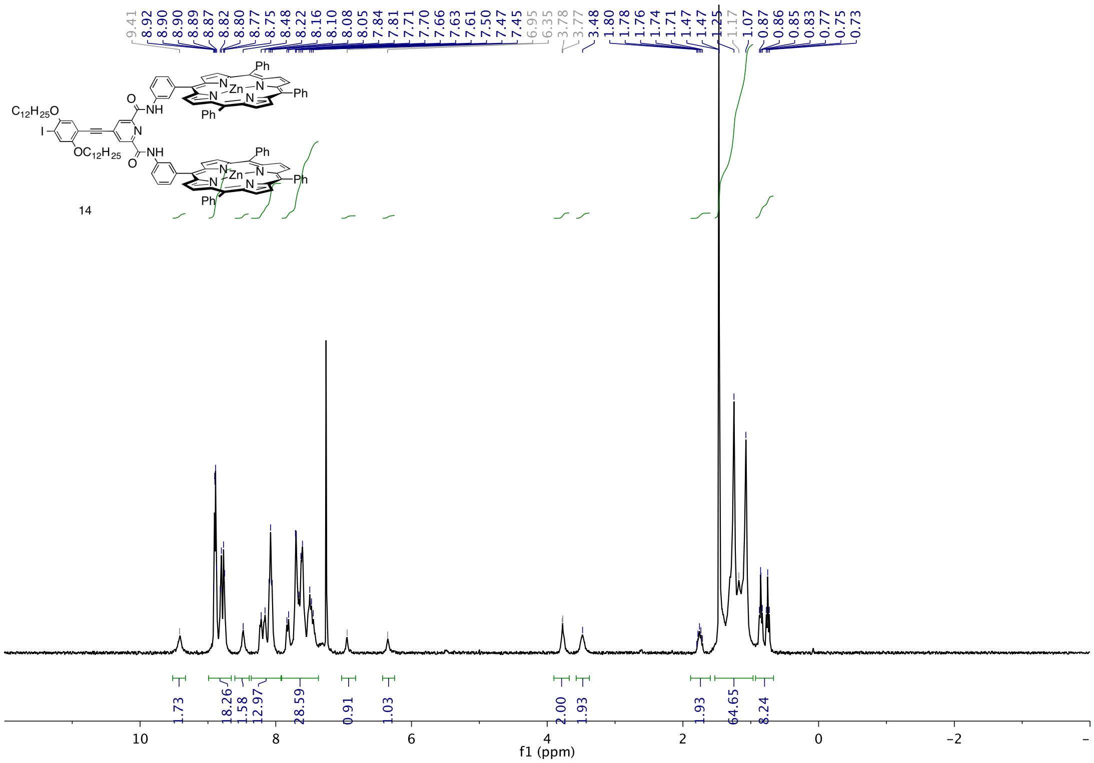

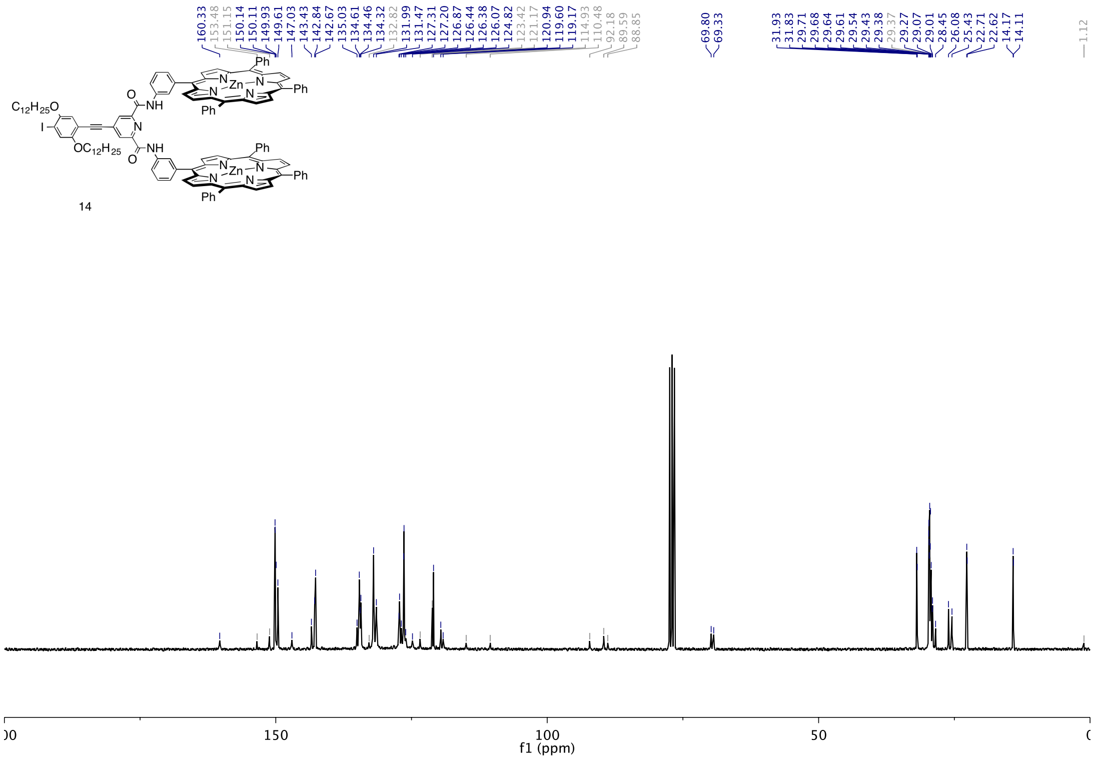

Supplementary Figure 73. ^{1}H and ^{13}C NMR spectra of **14** in chloroform-d_1.

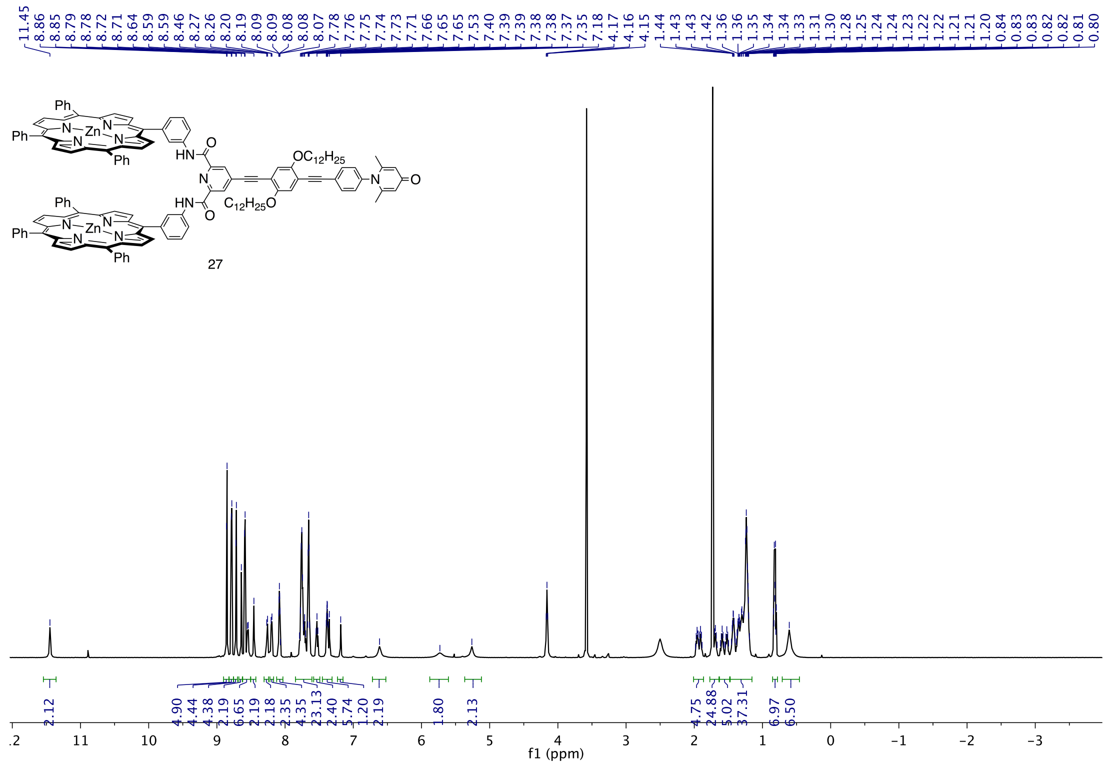

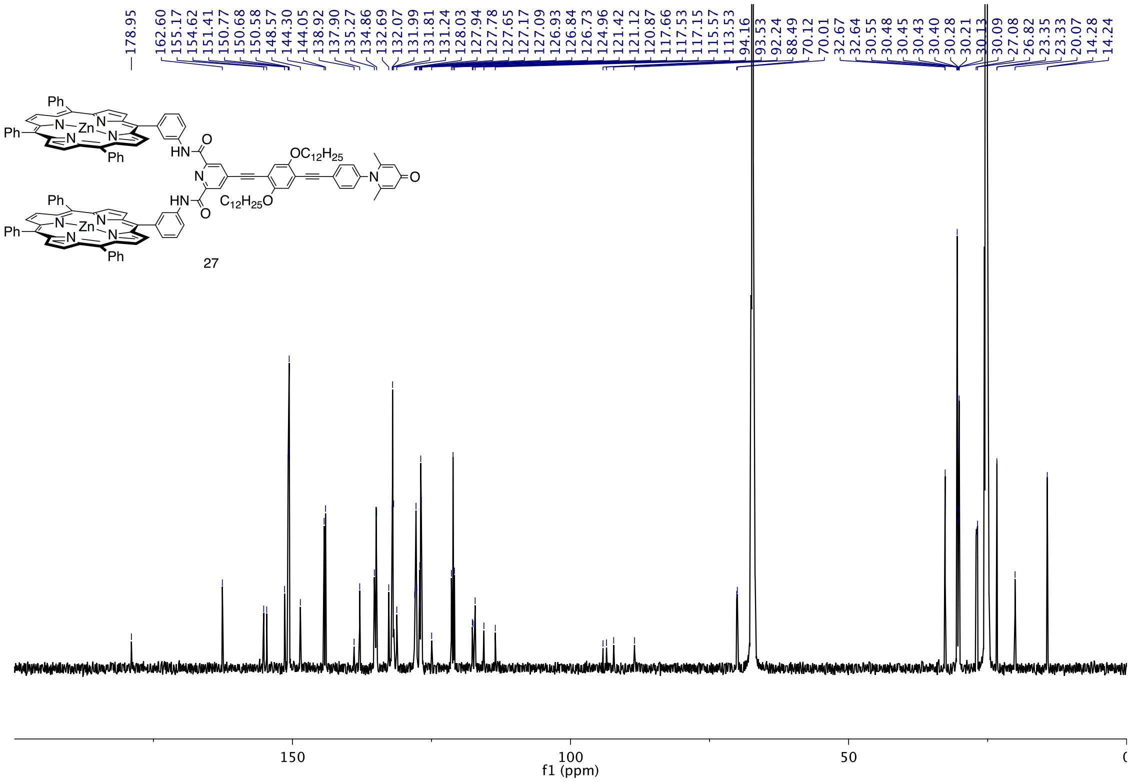

Supplementary Figure 74. ^{1}H and ^{13}C NMR spectra of **27** in THF-d_8.

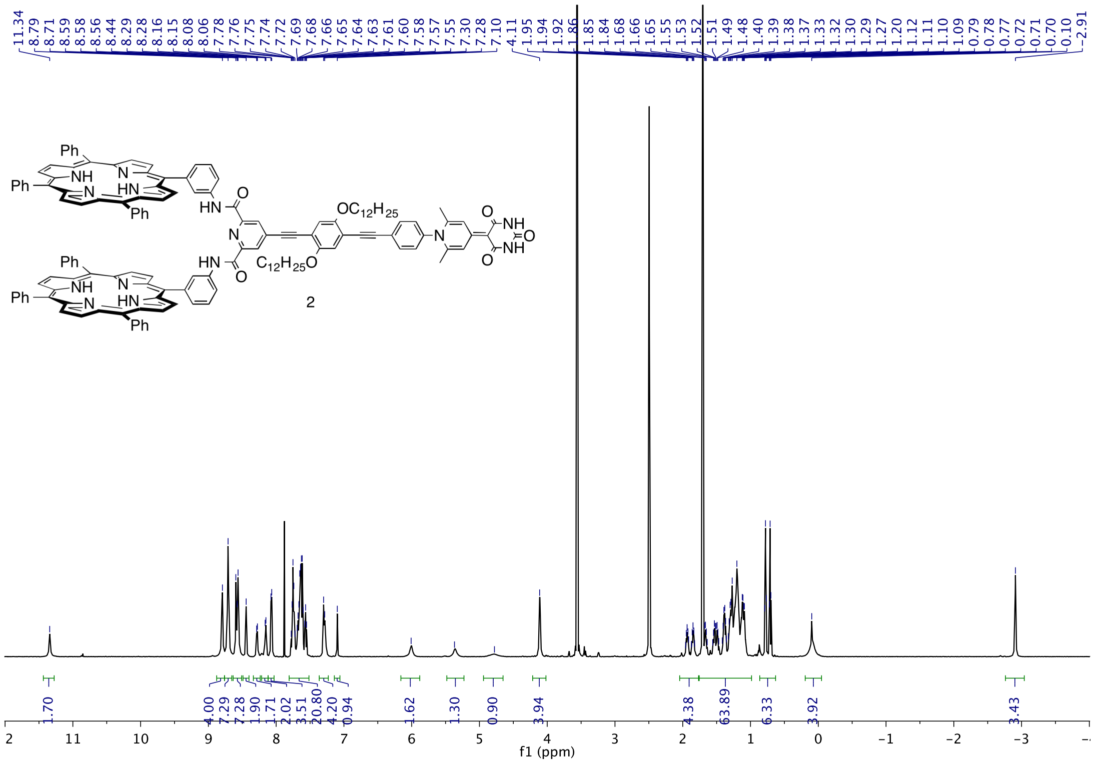

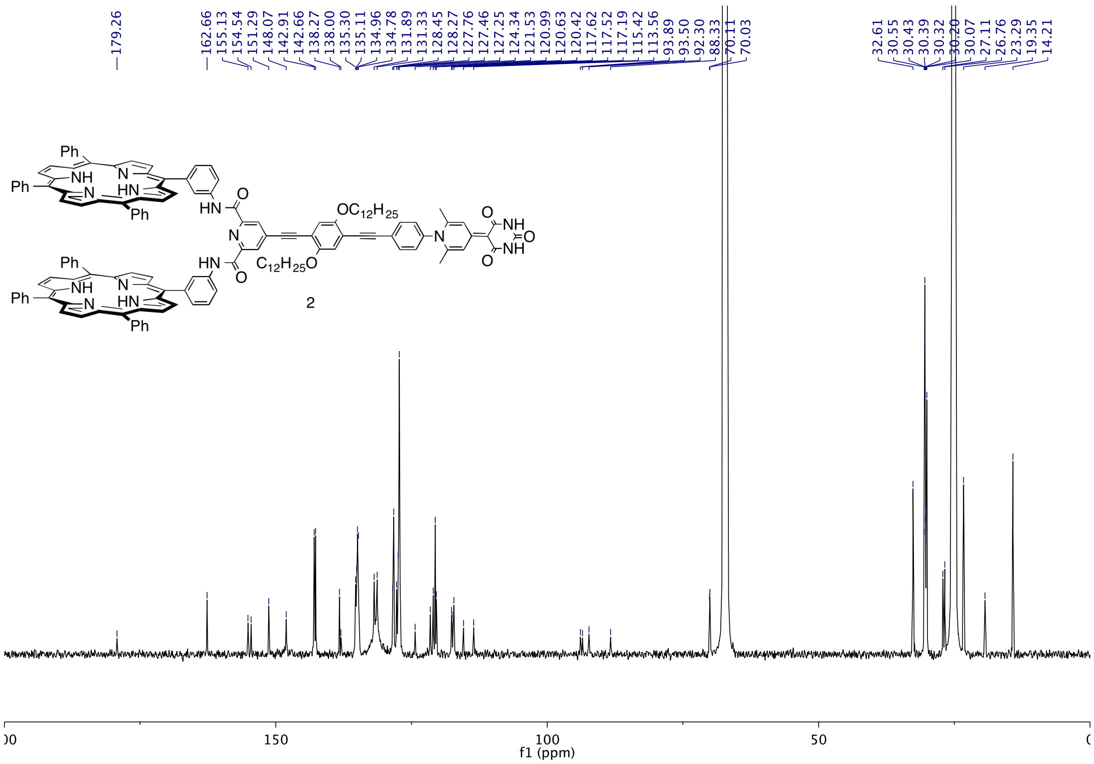

Supplementary Figure 75. ^{1}H and ^{13}C NMR spectra of **2** in THF-d_8.

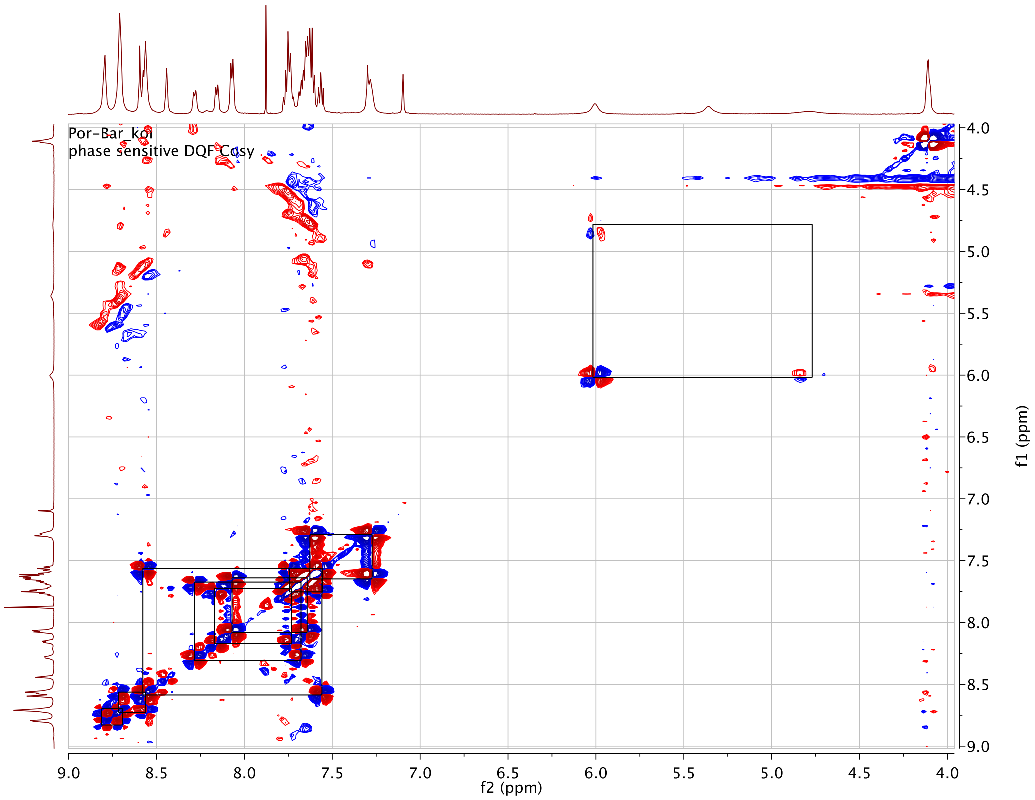

Supplementary Figure 76. DQF-COSY spectrum of **2** in THF-d_8.

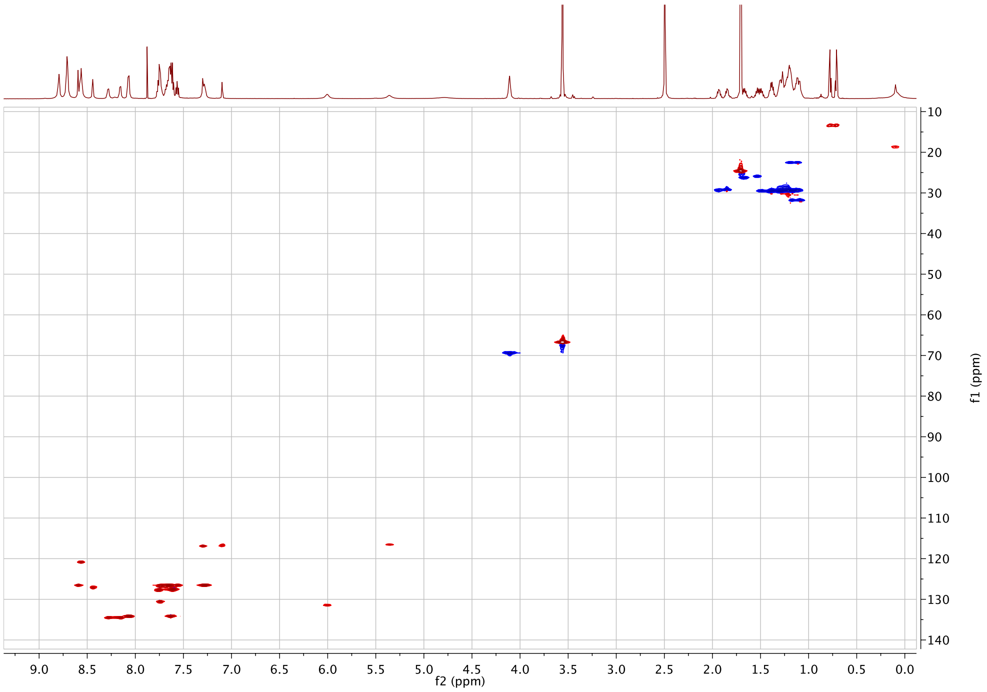

Supplementary Figure 77. HSQC spectra of **2** in THF-d_8. CH_3 and CH peaks are phased up (red), and CH_2 carbons are phase down (blue).

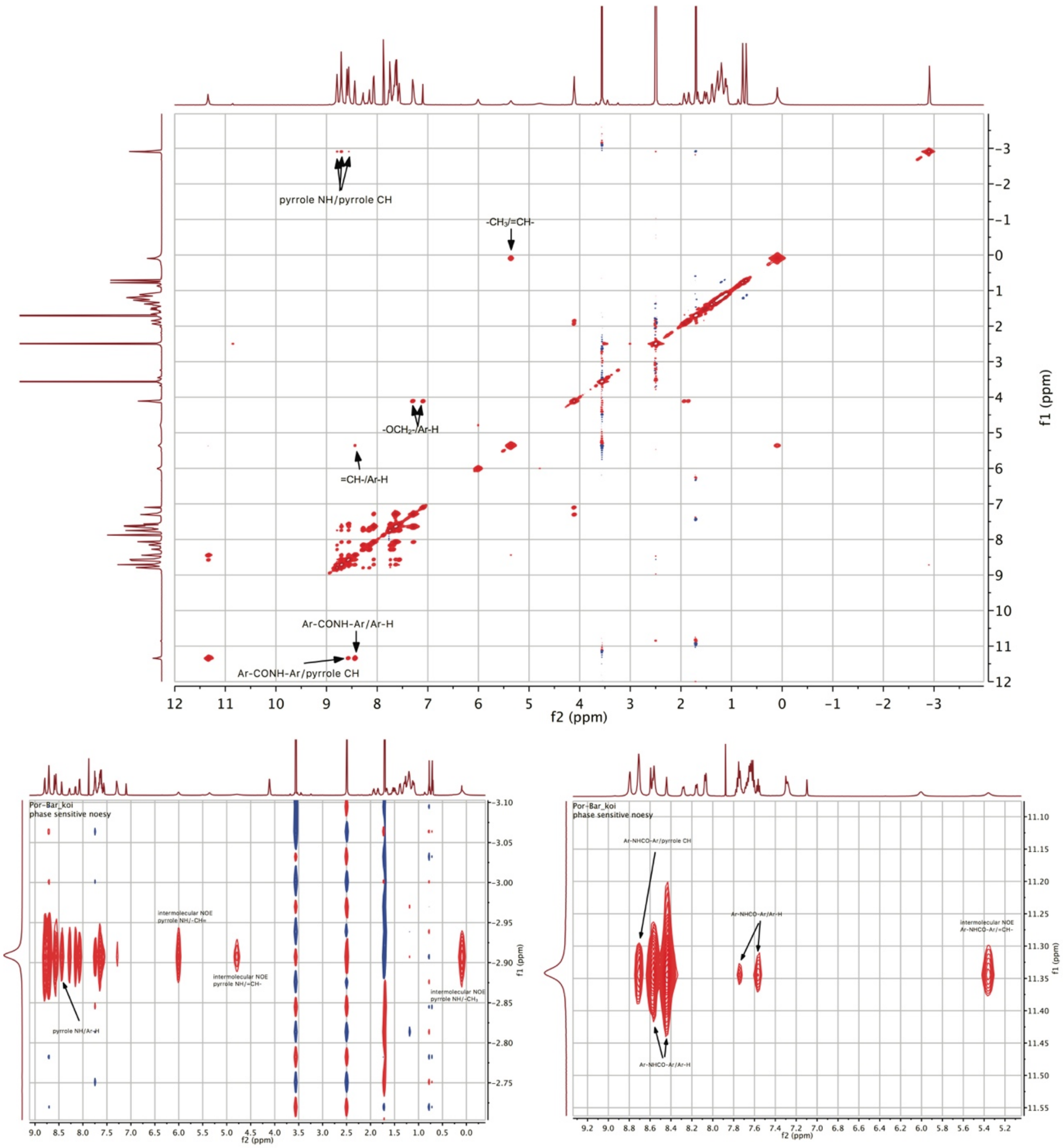

Supplementary Figure 78. NOESY spectrum of **2** in THF-d_8.

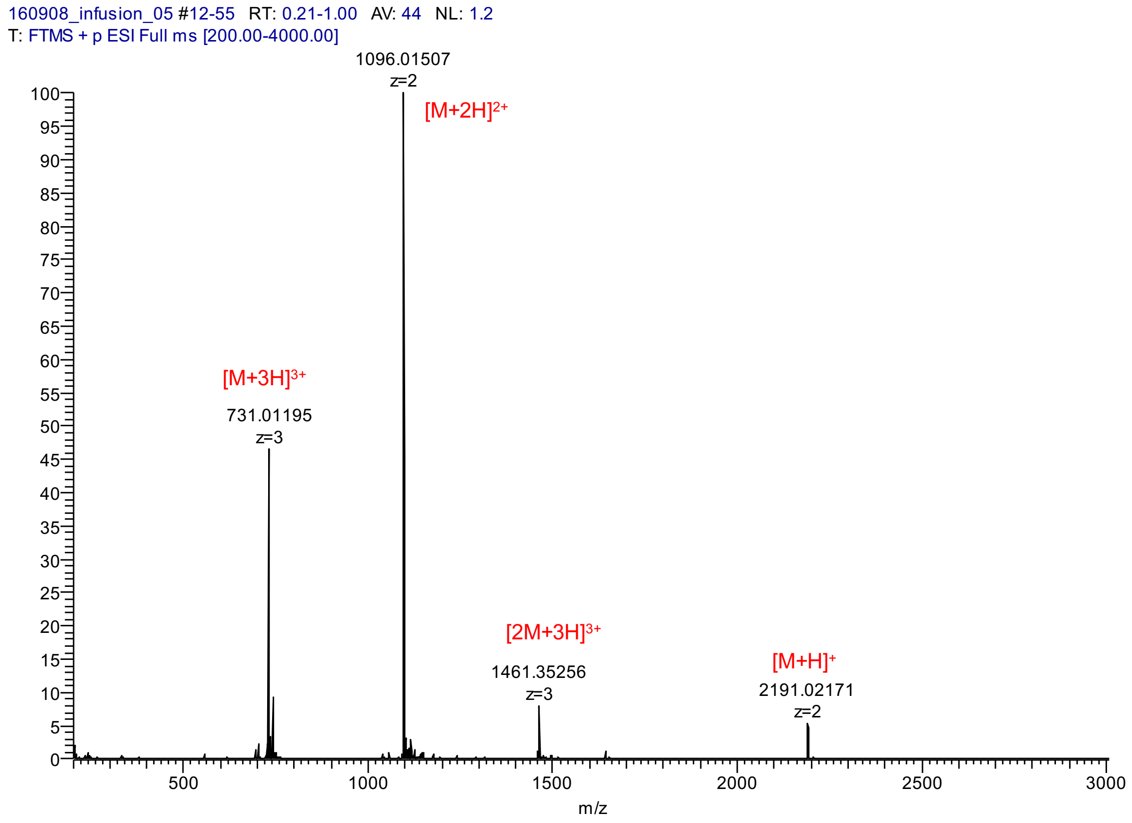

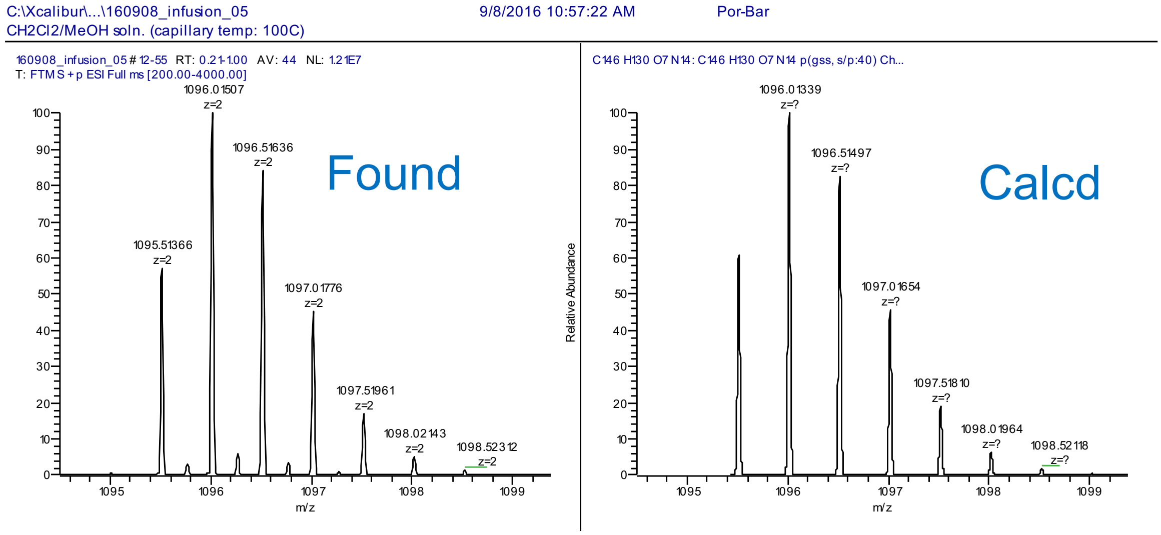

Supplementary Figure 79. MS spectra of **2**.

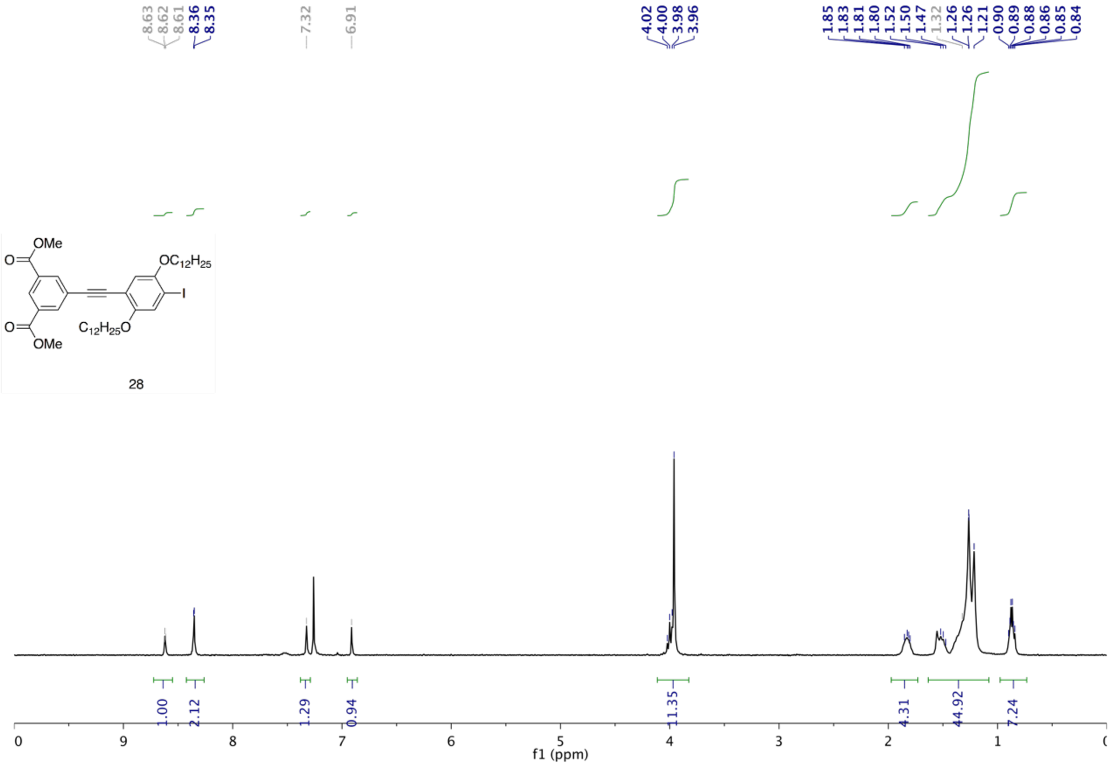

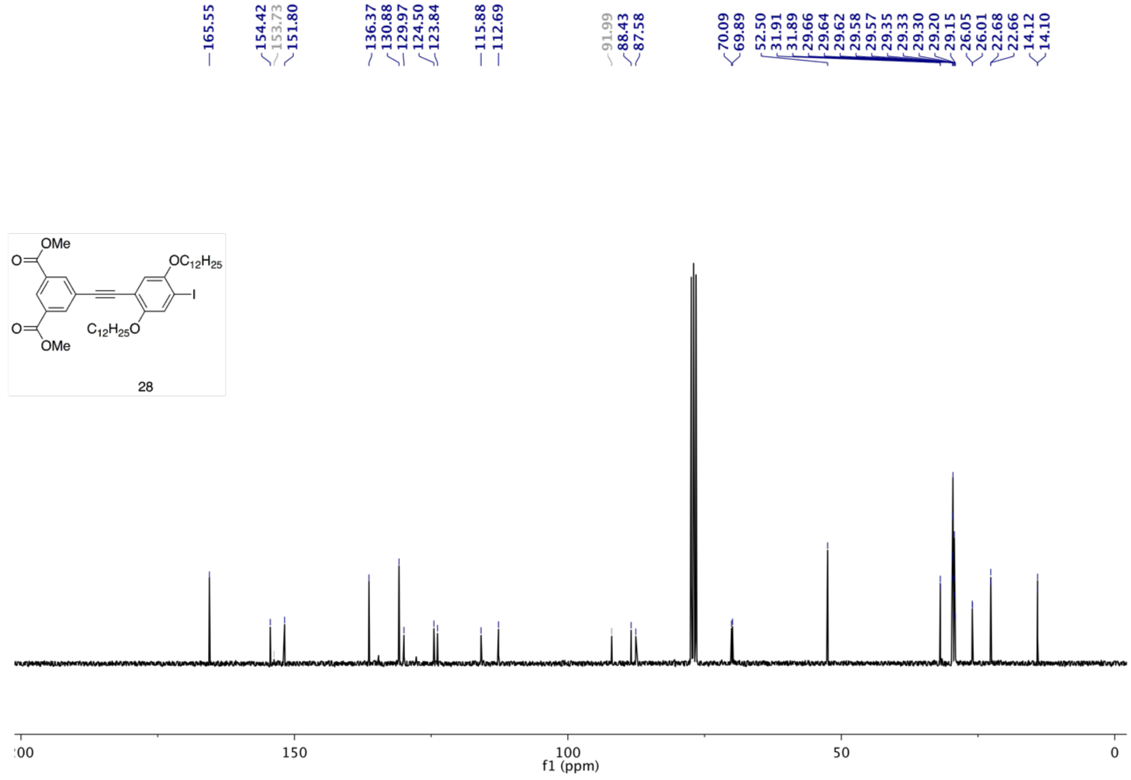

Supplementary Figure 80. ^{1}H and ^{13}C NMR spectra of **28** in chloroform-d_1.

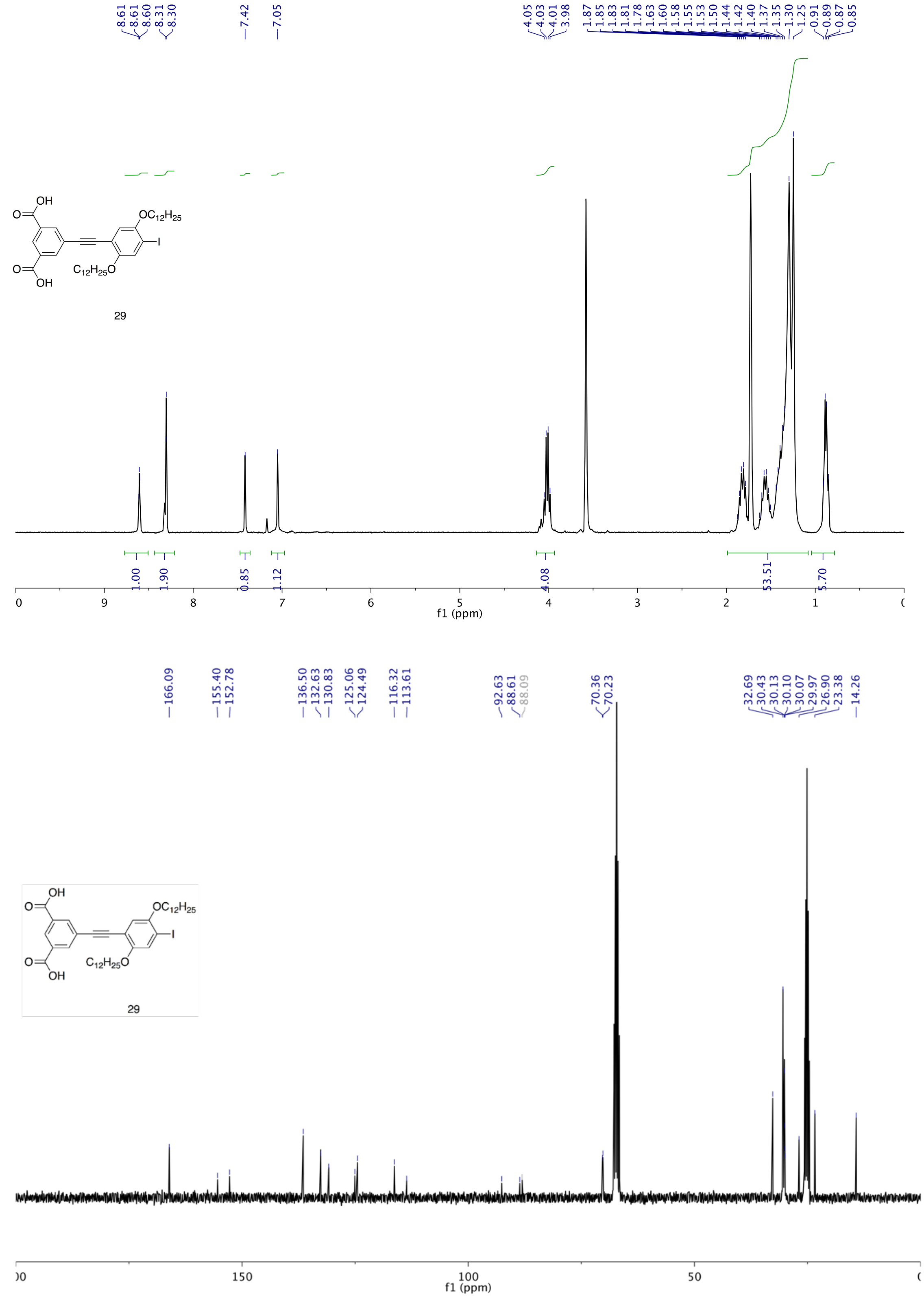

Supplementary Figure 81. ^{1}H and ^{13}C NMR spectra of **29** in THF-d_8.

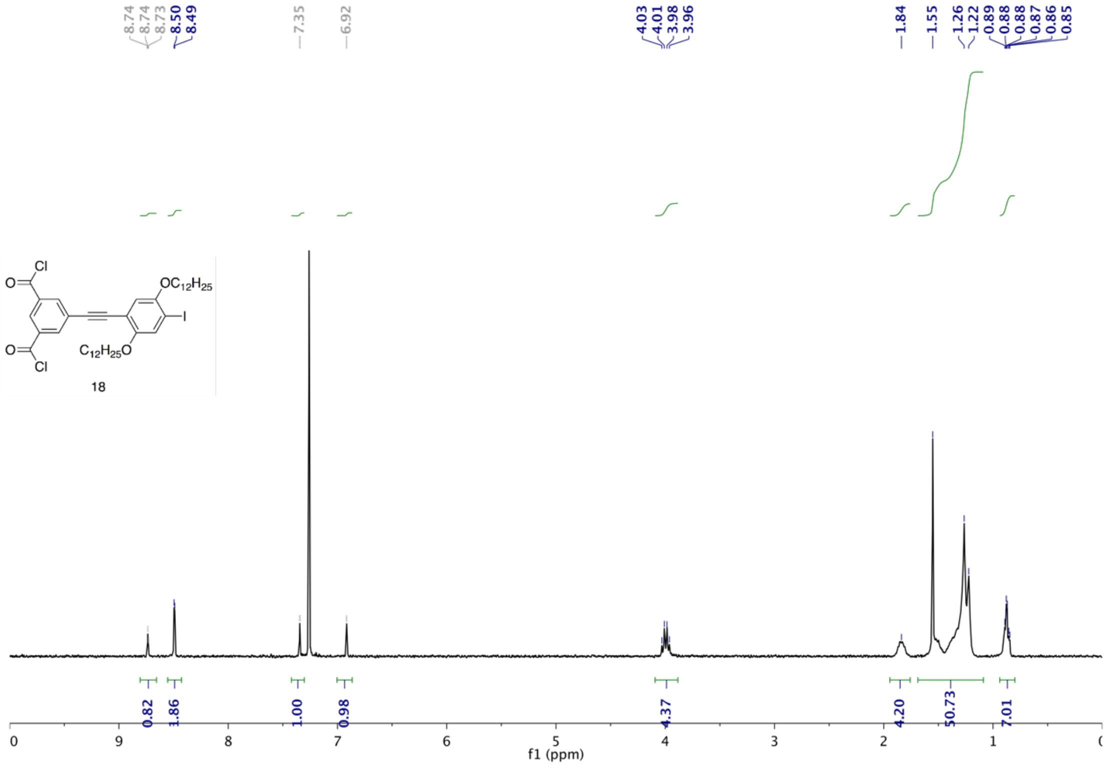

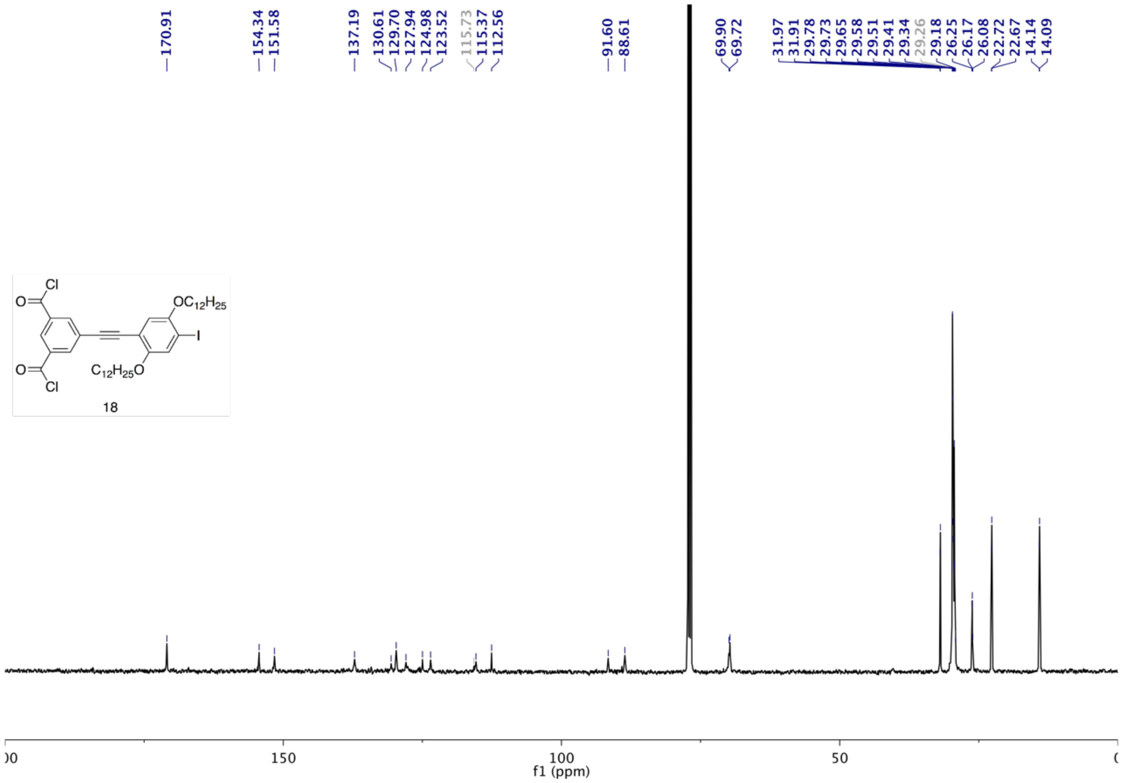

Supplementary Figure 82. ^{1}H and ^{13}C NMR spectra of **18** in chloroform-d_1.

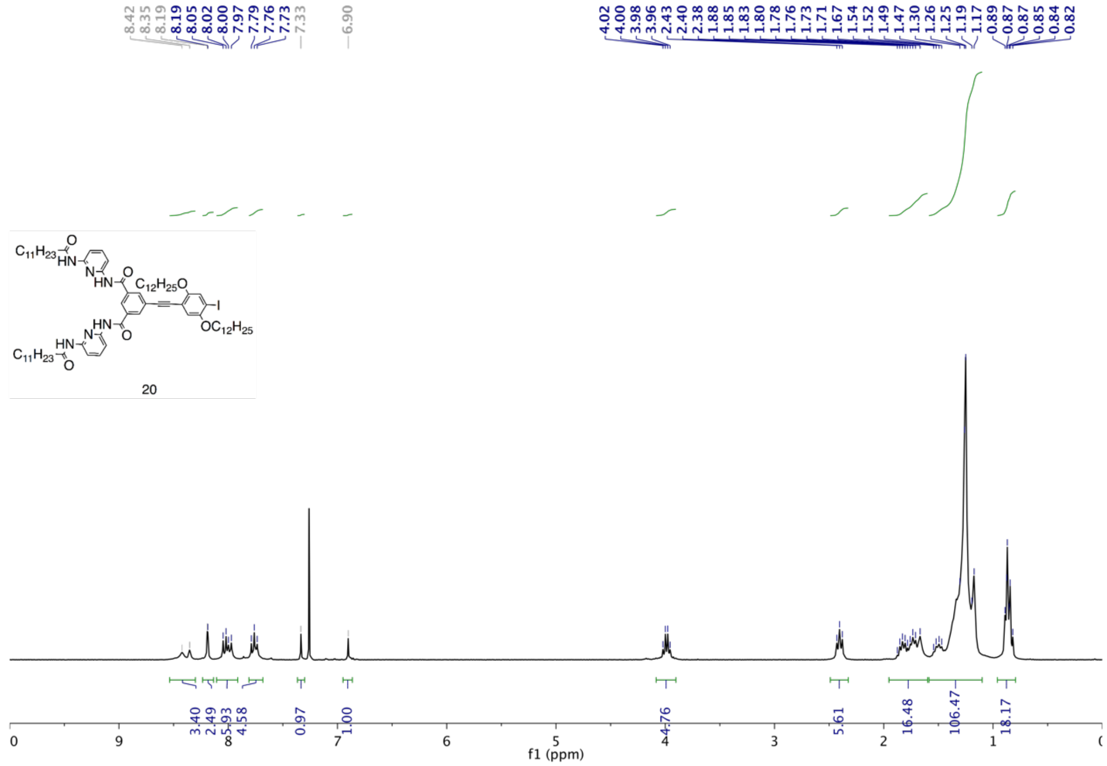

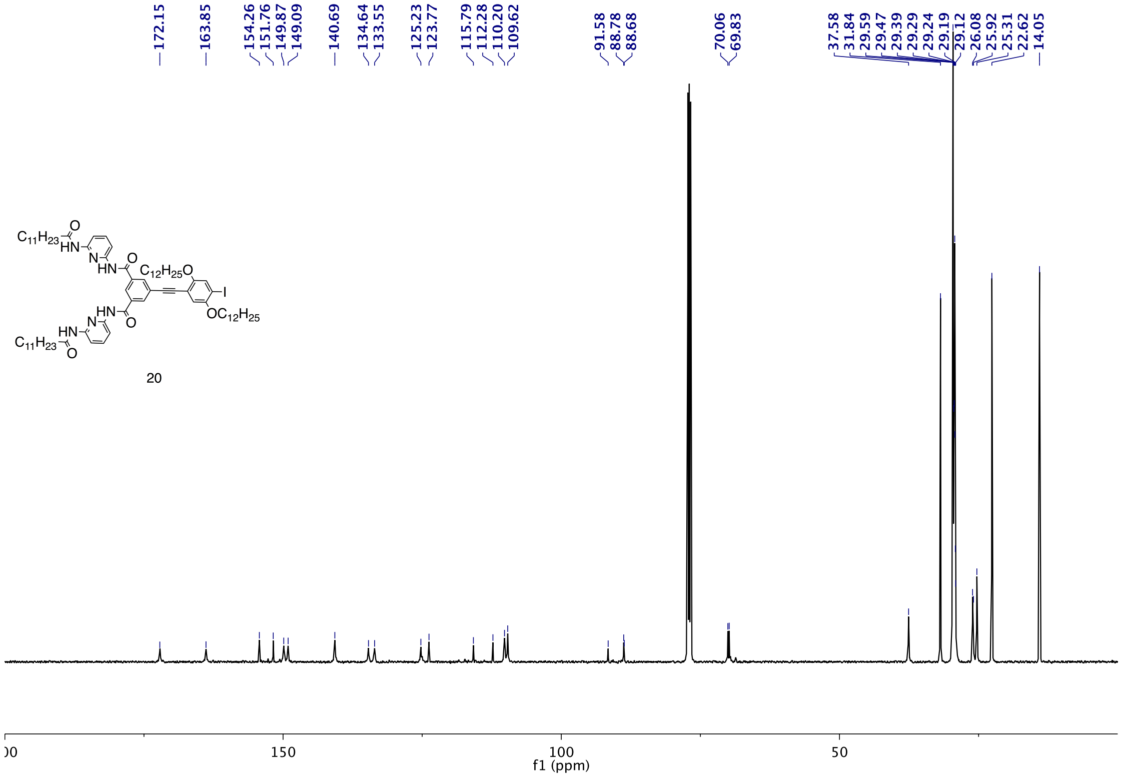

Supplementary Figure 83. ^{1}H and ^{13}C NMR spectra of **20** in chloroform-d_1.

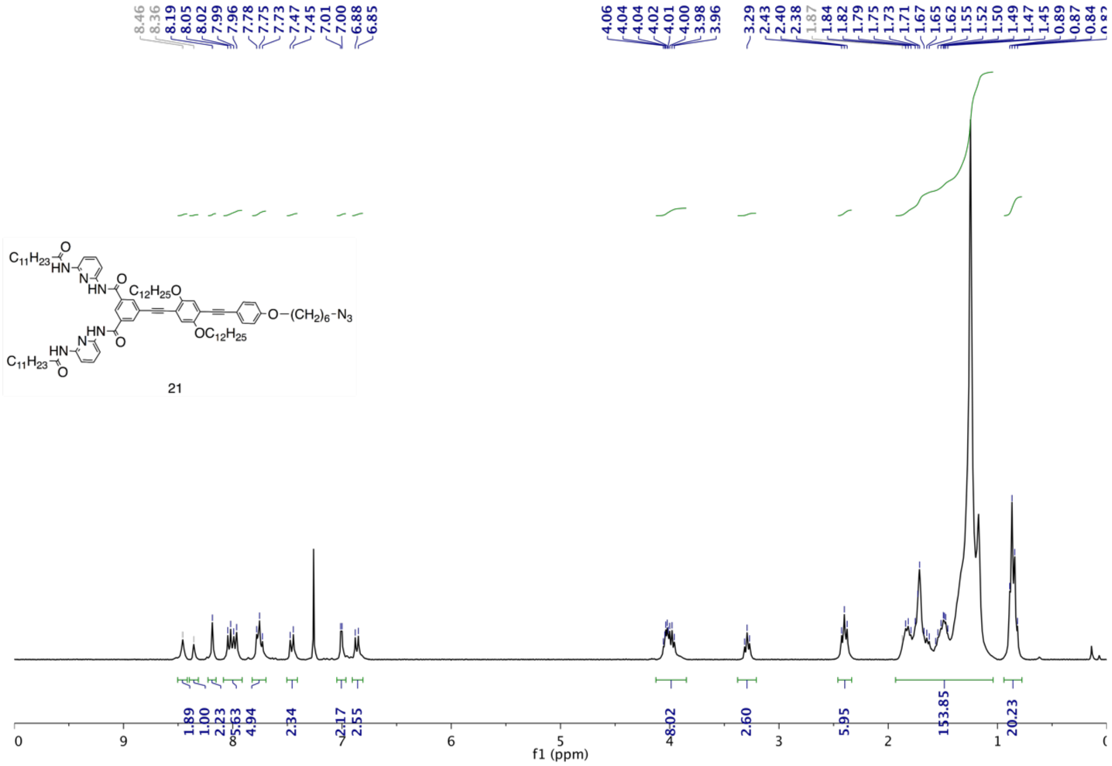

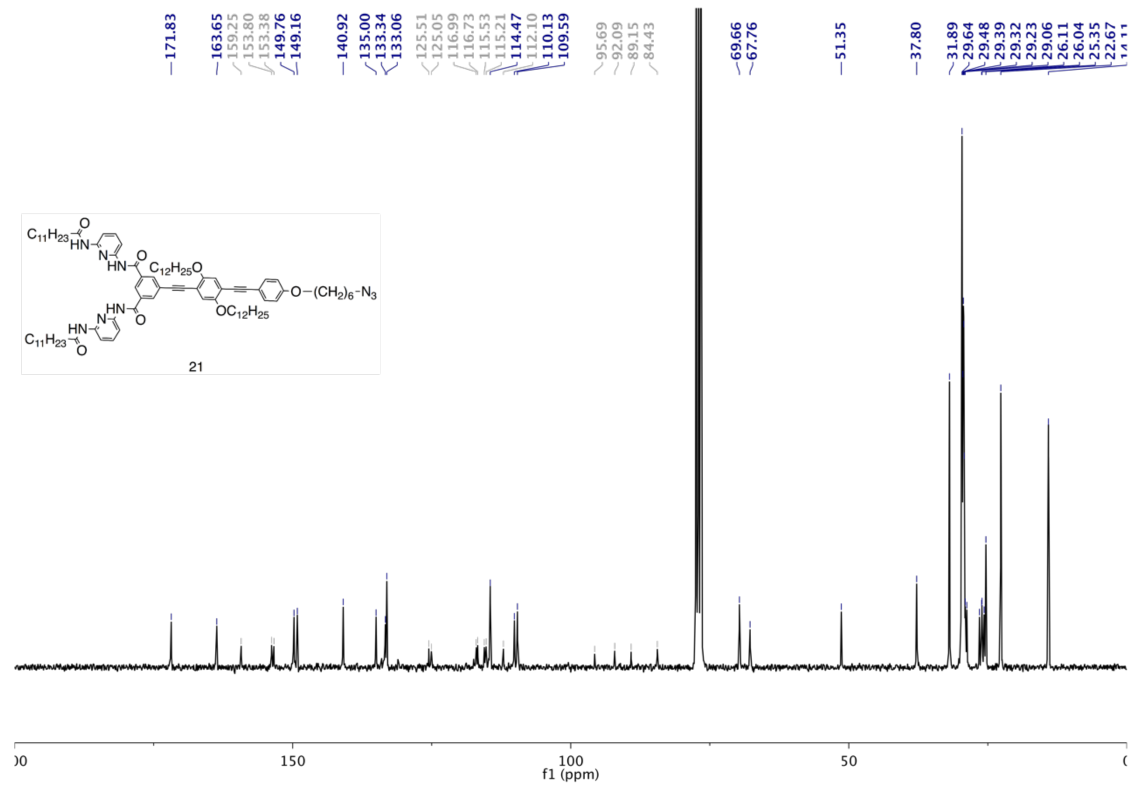

Supplementary Figure 84. ^{1}H and ^{13}C NMR spectra of **21** in chloroform-d_1.

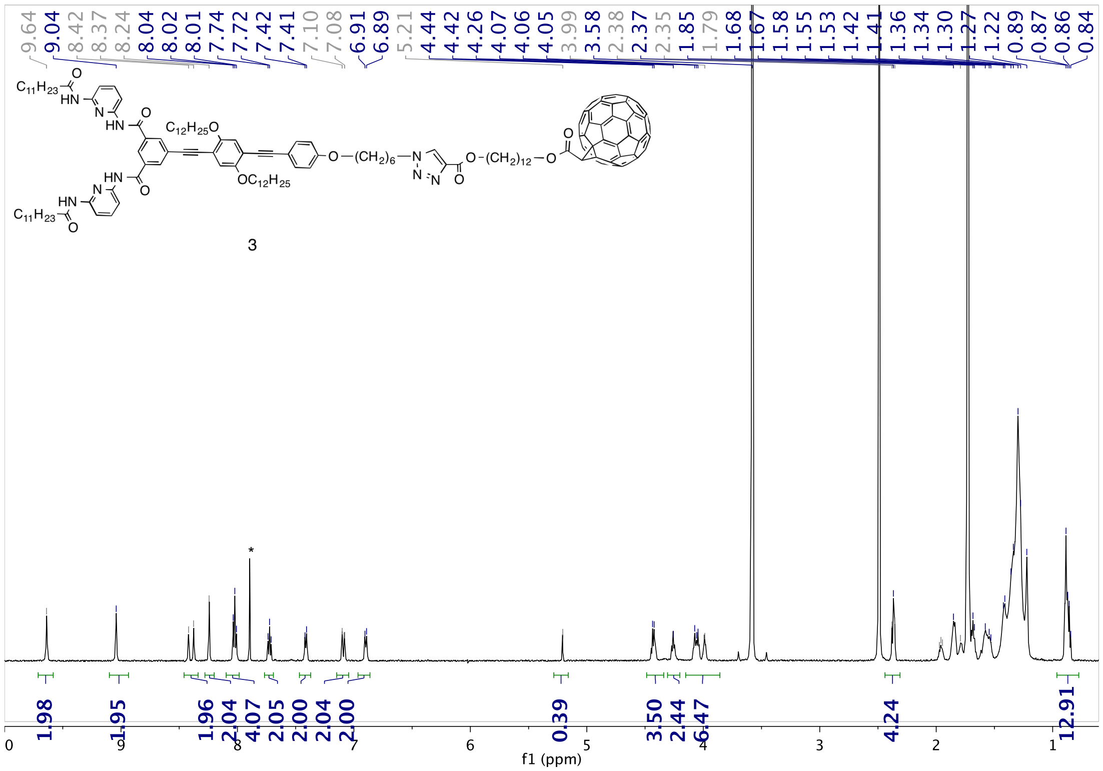

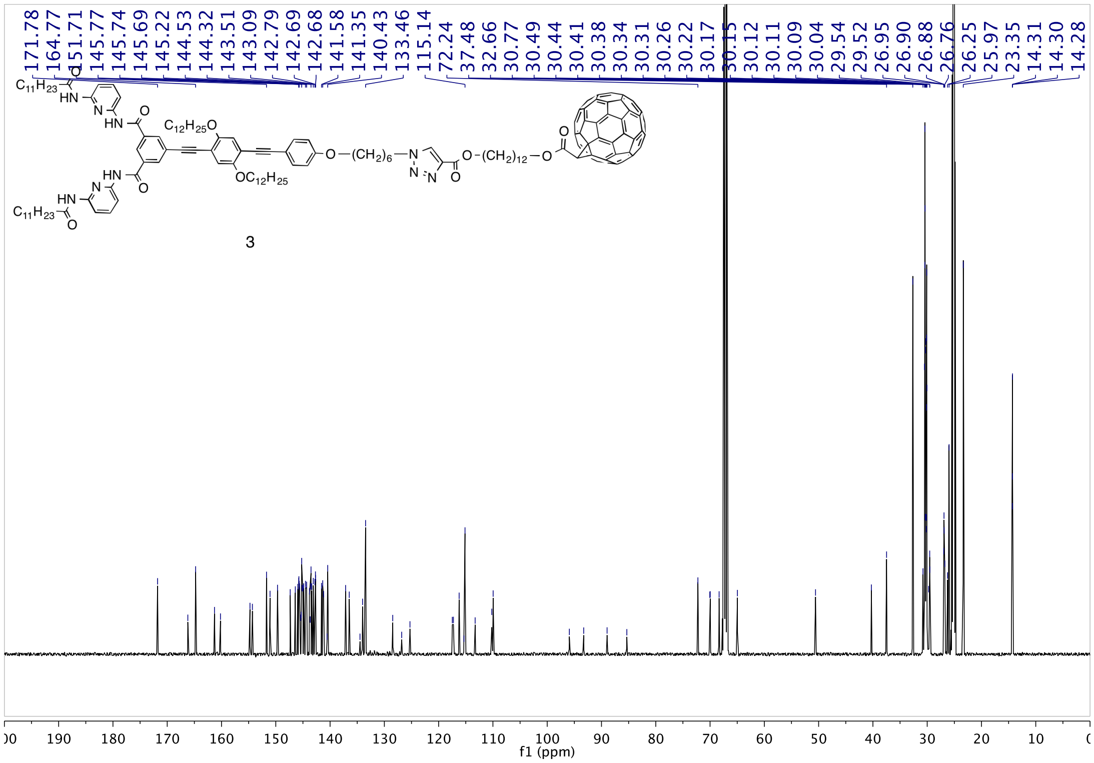

Supplementary Figure 85. ^{1}H and ^{13}C NMR spectra of **3** in THF-d_8. * denotes residual chloroform.

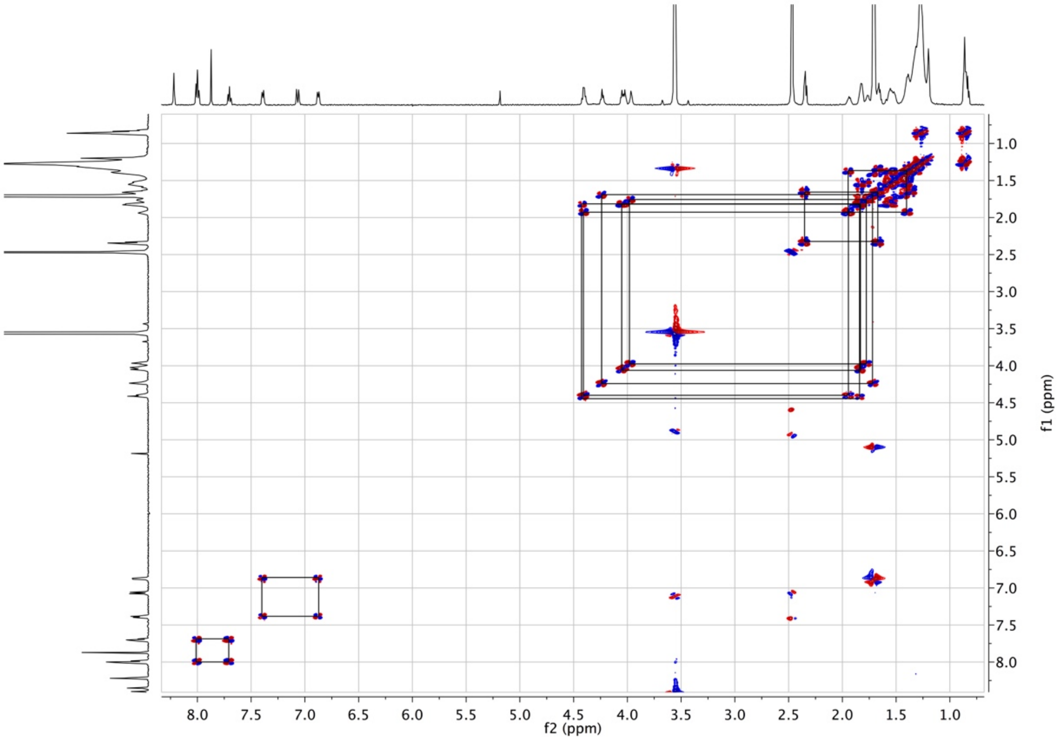

Supplementary Figure 86. DQF-COSY spectrum of **3** in THF-d_8.

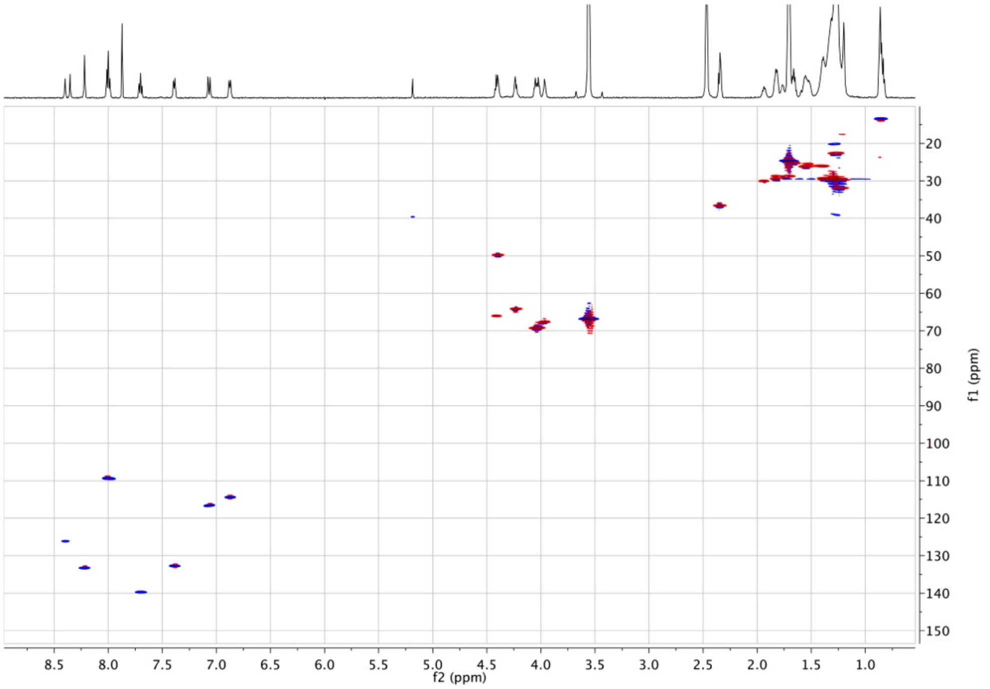

Supplementary Figure 87. HSQC spectrum of **3** in THF-d_8. CH_3 and CH peaks are phased up (blue), and CH_2 carbons are phase down (red).

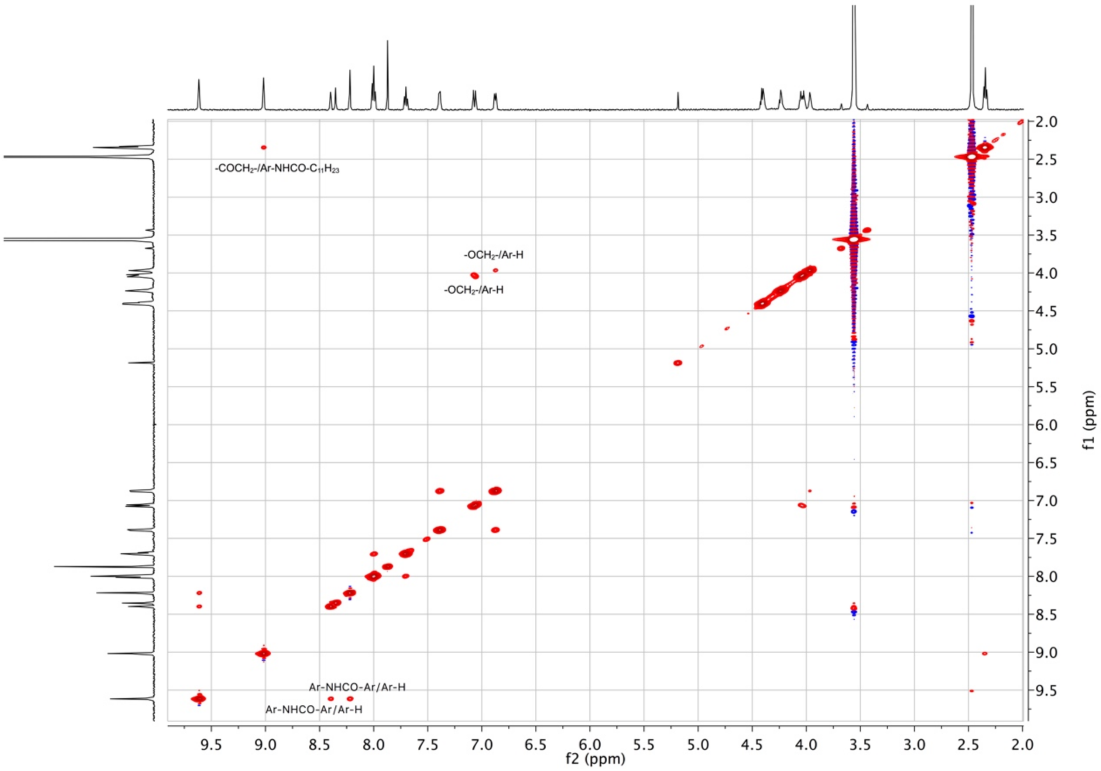

Supplementary Figure 88. NOESY spectrum of **3** in THF-d_8.

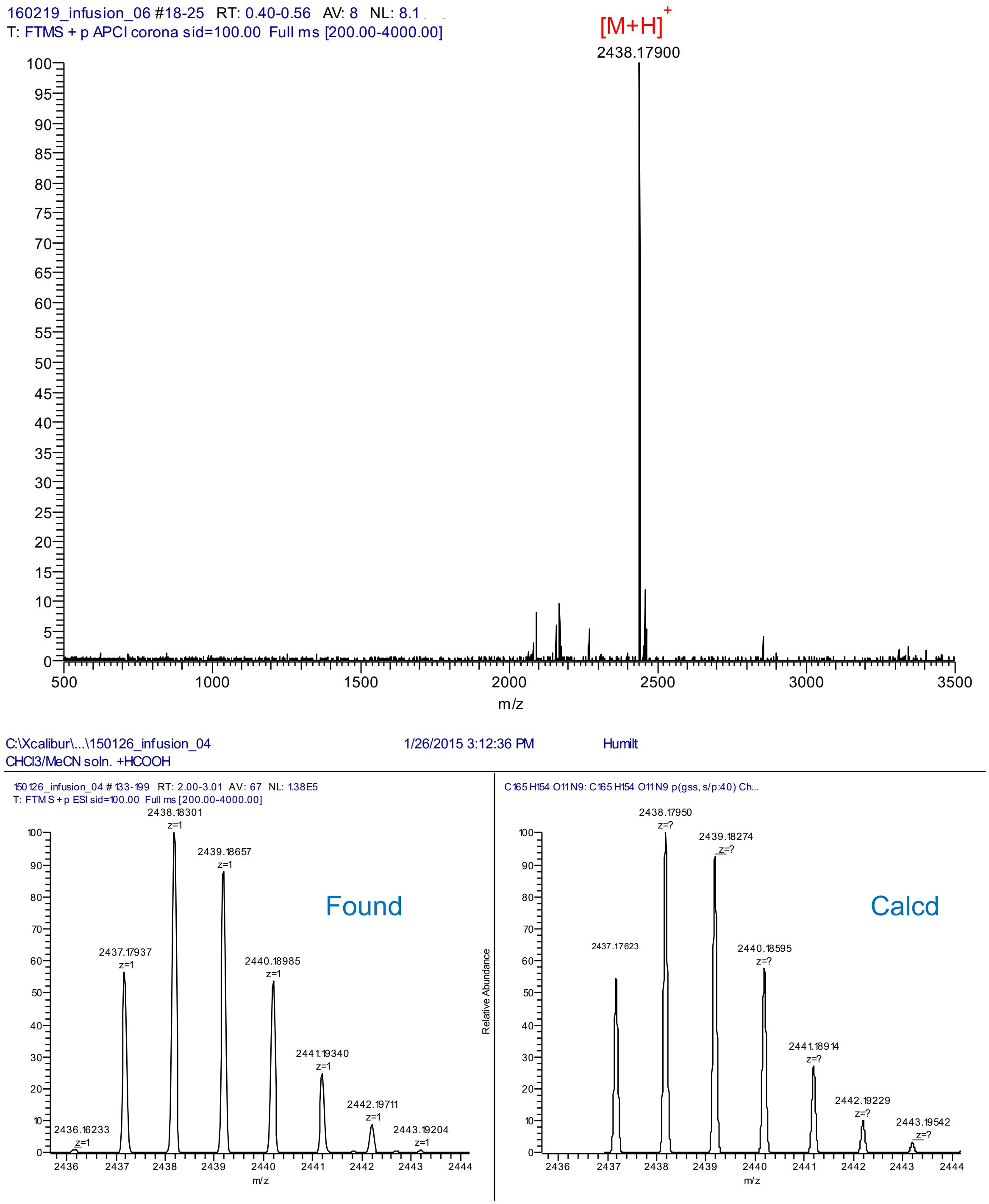

Supplementary Figure 89. MS spectra of **3**

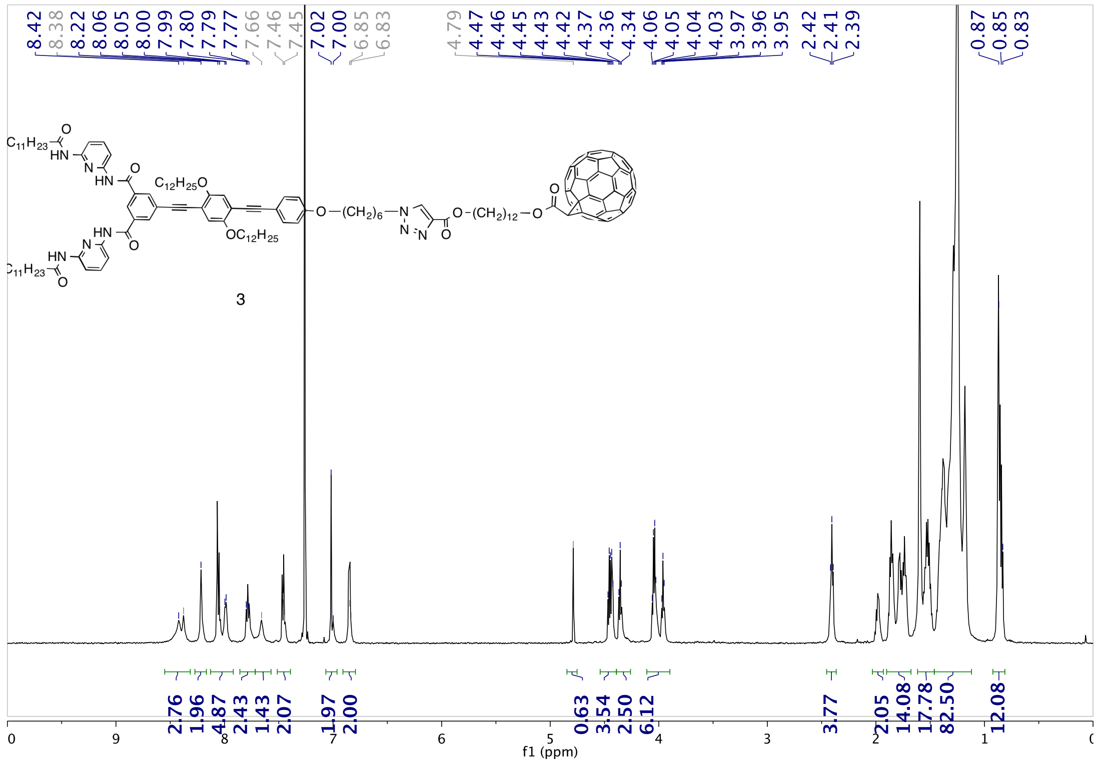

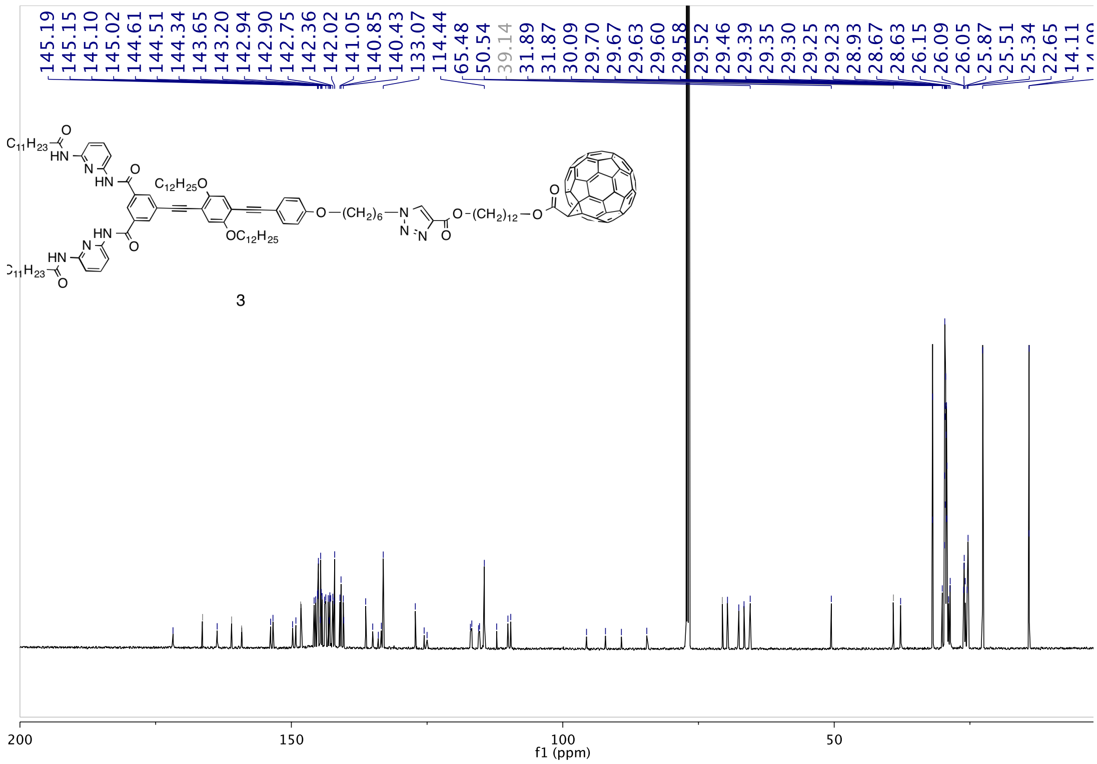

Supplementary Figure 90. ^{1}H and ^{13}C NMR spectra of **3** in chloroform-d_1.

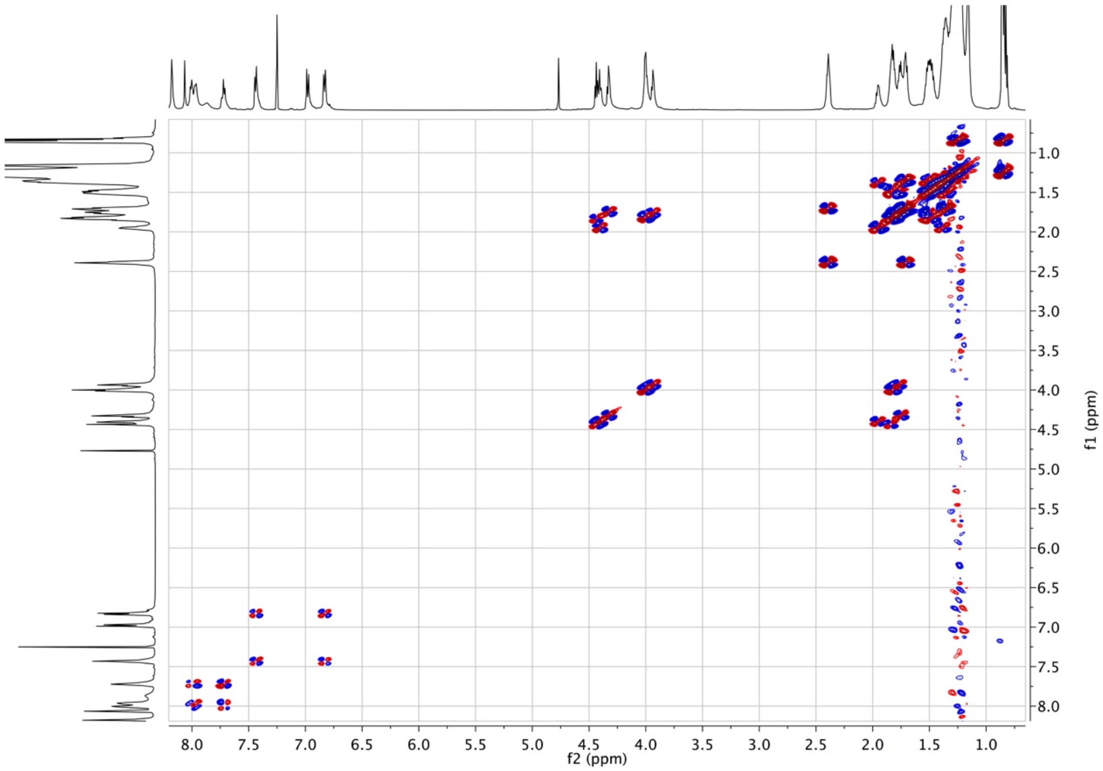

Supplementary Figure 91. DQF-COSY spectrum of **3** in chloroform-d_1.

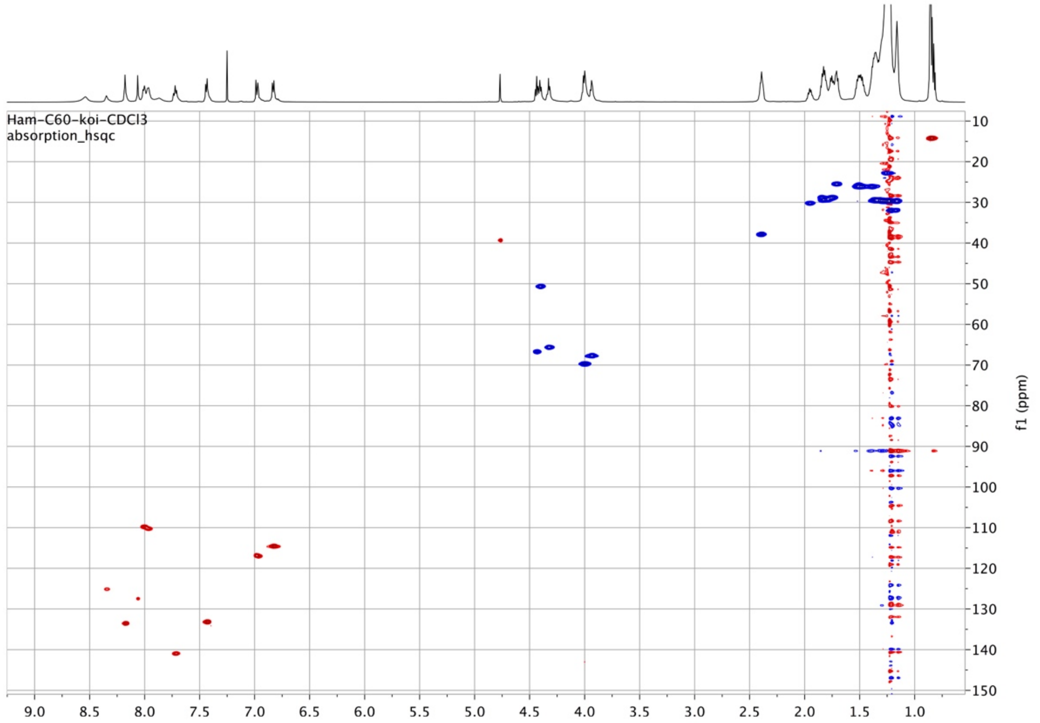

Supplementary Figure 92. HSQC spectrum of **3** in chloroform-d_1. CH_3 and CH peaks are phased up (red), and CH_2 carbons are phase down (blue).

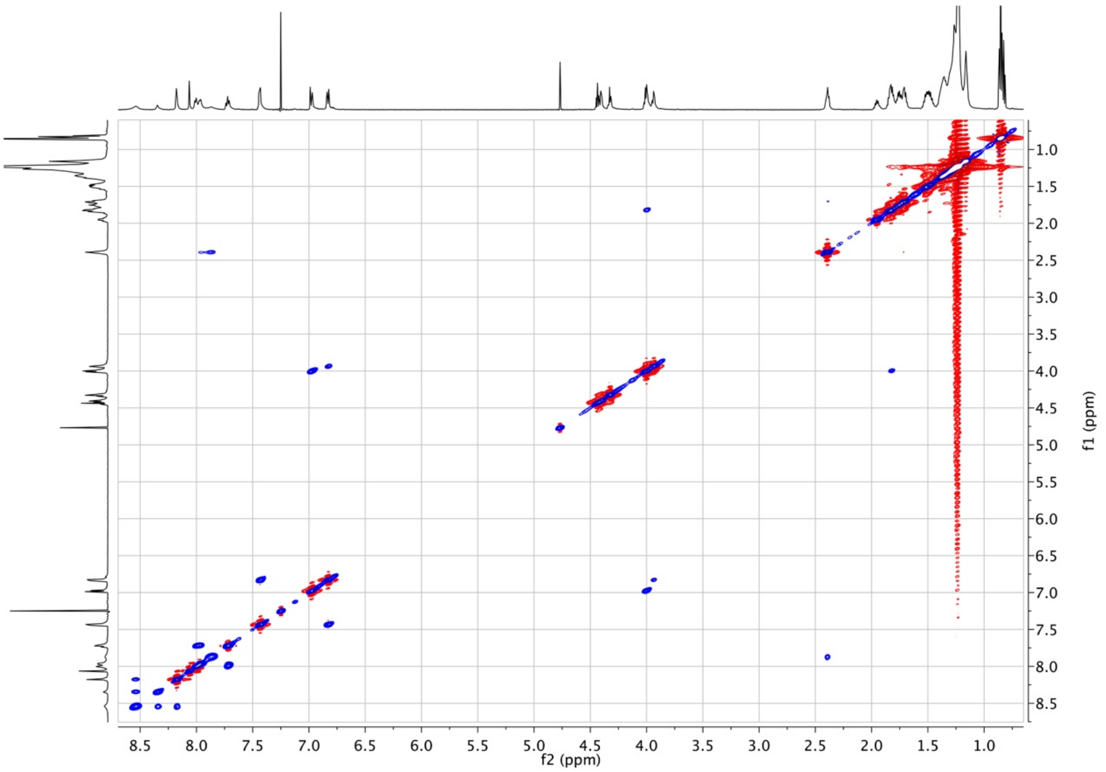

Supplementary Figure 93. NOESY spectrum of **3** in chloroform-d_1.

Supplementary References

1 Haino, T., Watanabe, A., Hirao, T. & Ikeda, T. Supramolecular Polymerization Triggered by Molecular Recognition between Bisporphyrin and Trinitrofluorenone. *Angew. Chem. Int. Ed.* **51**, 1473-1476 (2012).

2 Sivakova, S., Wu, J., Campo, C. J., Mather, P. T. & Rowan, S. J. Liquid-crystalline supramolecular polymers formed through complementary nucleobase-pair interactions. *Chem. Eur. J.* **12**, 446-456 (2006).

3 Hirao, T., Tosaka, M., Yamago, S. & Haino, T. Supramolecular Fullerene Polymers and Networks Directed by Molecular Recognition between Calix[5]arene and C_{60}. *Chem. Eur. J.* **20**, 16138-16146 (2014).

4 Holik, M., Paveska, P. & Mlynarik, V. Transfer of Substituent Effects in N-/P-Substituted Phenyl/-2,6-Dimethyl-4-Pyridones. *J. Mol. Struct.* **114**, 15-20 (1984).

5 Hamada, T., Tanaka, S., Koga, H., Sakai, Y. & Sakaki, S. Kinetic study of the photo-induced electron transfer reaction between ruthenium(II) complexes of 2,2'-bipyridine derivatives and methyl viologen. Effects of bulky substituents introduced onto 2,2'-bipyridine. *Dalton Trans.*, 692-698 (2003).

6 Lamberto, M., Pagba, C., Piotrowiak, P. & Galoppini, E. Synthesis of novel rigid-rod and tripodal azulene chromophores. *Tetrahedron Lett.* **46**, 4895-4899 (2005).

7 Daoud, W. A. & Turner, M. L. Efficient synthesis of 1,4-dialkoxy and 1,4-dialkyl substituted 2,5-divinylbenzenes via the Stille reaction. *Bull. Chem. Soc. Jpn.* **78**, 367-369 (2005).

8 Mibu, N. *et al.* N-long-chain monoacylated derivatives of 2,6-diaminopyridine with antiviral activity. *Chem. Pharm. Bull.* **55**, 111-114 (2007).

9 Yan, H. *et al.* Side-chain fullerene polyesters: a new class of high refractive index polymers. *Mater. Horiz.* **1**, 247-250 (2014).

10 Tada, T., Ishida, Y. & Saigo, K. Synthesis and reactions of 2,2-[60]fullerenoalkanoyl chlorides. *J. Org. Chem.* **71**, 1633-1639 (2006).
